# Global, regional, and national burden of diabetes in women of childbearing age, 1990–2021: a systematic analysis from the global burden of disease study 2021

**DOI:** 10.3389/fgwh.2025.1528661

**Published:** 2025-07-15

**Authors:** Zhifeng Guo, Wangquan Ji, Mengqing Yan, Xianan Zou, Teng Chen, Fanghui Bai, Yu Wu, Zhe Guo, Linlin Song

**Affiliations:** ^1^ Nanyang Maternal and Child Health Care Hospital, Nanyang Central Hospital, Nanyang, China; ^2^ College of Public Health, Zhengzhou University, Zhengzhou, China; ^3^ Research Department, Nanyang Central Hospital, Nanyang, China

**Keywords:** diabetes, global burden, women of childbearing age, prevalence, disability-adjusted life year

## Abstract

**Introduction:**

Diabetes may have long-term adverse health effects on both women of childbearing age (WCBA) and their future generations. The objective of this study is to provide up-to-date epidemiologic information on the global burden of diabetes in WCBA to inform the development of targeted public health policies.

**Methods:**

The data on the burden of diabetes among WCBA from 1990 to 2021 at the global, regional, and national levels were extracted from the Global Burden of Disease 2021 database. The estimated annual percentage change (EAPC) and Bayesian age–period–cohort models were used to assess and predict time burden trends. The slope index and concentration index were used to assess health inequalities associated with the sociodemographic index (SDI).

**Results:**

In 2021, approximately 79.04 million WCBA aged 15–49 years were living with diabetes, resulting in approximately 7.82 million disability-adjusted life years (DALYs). From 1990 to 2021, the age-standardized prevalence rate (ASPR) increased from 1960.8 to 3942.2 per 100,000 WCBA, with an EAPC of 2.25%. The ASPR and age-standardized DALY rate were highest in the low-middle SDI region, at 4,107.0 and 472.3 per 100,000, respectively. DALYs and deaths are concentrated in low SDI countries. By 2040, the global burden of diabetes in WCBA will increase further.

**Conclusion:**

The global burden of diabetes among WCBA has increased over the past three decades. This burden is concentrated in low- and middle-income countries. Diabetes care policies for WCBA urgently need to be improved and popularized.

## 1 Introduction

Diabetes is a global public health problem and a significant contributor to the global burden of disease. The Global Burden of Disease Study (GBD) 2021 estimates that in 2021, there were 529 million people living with diabetes globally, resulting in 79 million global disability-adjusted life years (DALYs) (1). By 2050, more than 1,310 million people are predicted to have diabetes (1). The International Diabetes Federation estimates that diabetes led to $966 billion in global healthcare spending in 2021 and is predicted to exceed $1,054 billion by 2045 (2). Preventing further exacerbation of the global burden of diabetes has always been an important issue in the field of global health.

Diabetes can lead to a variety of diseases or complications, and for women of childbearing age (WCBA), diabetes is also associated with an increased risk of menstrual dysfunction, polycystic ovary syndrome, and reduced fertility (3–5). In addition, pregnant women with diabetes or hyperglycemia may be at increased risk for pregnancy complications and adverse birth outcomes, such as gestational hypertension, preeclampsia, miscarriage, preterm labor, birth defects, and fetal macrosomia, which pose a serious threat to the health of the mother and her offspring (6, 7). The increasing prevalence of diabetes globally exacerbates the health burdens faced by WCBA, with adverse implications for maternal and fetal well-being. Preventing, managing, and treating diabetes in WCBA, especially those planning pregnancy, is crucial for improving perinatal outcomes and ensuring the health of both mothers and children (8–10). Therefore, it is important to assess the burden of diabetes on WCBA to implement precise intervention activities on a global scale.

Type 1 diabetes and type 2 diabetes are the most common types of diabetes. Previous studies have focused predominantly on the burden of type 1 diabetes or type 2 diabetes across all age groups (1, 11, 12), with some specifically examining the impact on adolescents or the elderly (13, 14). However, there is a notable absence of research on the global burden of diabetes in WCBA. In summary, this study, which is based on data from the GBD 2021 study, conducts a comprehensive analysis of the burden of diabetes in WCBA at the global, regional, and national levels, further exploring the regional and temporal distributions of this burden. This study provides a basis for policymakers to take targeted intervention measures for this specific population.

## 2 Materials and methods

### 2.1 Data sources

The data for this study are based on the GBD 2021, which can be obtained through the Global Health Data Exchange query tool (https://vizhub.healthdata.org/gbd-results/). GBD 2021 conducted a systematic and comparative assessment of the global burden of 288 causes of death and 371 diseases and injuries in 204 countries and territories from 1990 to 2021 (15, 16). In this study, global, regional, and national estimates of the prevalence, incidence, deaths, and DALYs for diabetes, including type 1 diabetes and type 2 diabetes, in WCBA aged 15–49 years from 1990 to 2021 were obtained using data from the GBD 2021. In addition, the GBD estimates reported in this study include 95% uncertainty intervals (UI) that reflect uncertainty due to data availability, data quality, and modeling differences. Estimates were obtained for each 5-year age group and used to calculate age-standardized rates (ASRs). ASRs are calculated based on the world standard population developed by the GBD (15, 16). In this study, we calculated the ASRs corresponding to prevalence, incidence, deaths, and DALYs, which are referred to as age-standardized prevalence rate (ASPR), age-standardized incidence rate (ASIR), age-standardized mortality rate (ASMR), and age-standardized DALY rate (ASDR), respectively.

### 2.2 SDI

The sociodemographic index (SDI) was calculated with distributional adjustments based on the fertility rate of young women, the educational attainment of women aged 15 years or older, and per capita income. The SDI was collected from GBD 2021 to measure the socioeconomic development status of a particular region (15, 16). The 204 countries and territories were then categorized into five regions based on the SDI, namely, low (<0.47), low-middle (0.47–0.62), middle (0.63–0.71), high-middle (0.72–0.81), and high (>0.81) (17).

### 2.3 Statistical analyses

The estimated annual percentage change (EAPC) was used to assess the trend in ASRs (18). When the lower 95% confidence interval (CI) of the EAPC is greater than 0, the ASR tends to increase; conversely, when the upper 95% CI of the EAPC is less than 0, the ASR tends to decrease.

Bayesian age-period-cohort (BAPC) analysis was used to predict the ASRs from 2022 to 2040. The BAPC model assumes that age, period, and cohort effects have similar impacts over adjacent time intervals. In the BAPC model, all unknown parameters are considered random with appropriate prior distributions. Bayesian inference employs a second-order random walk to smooth the prior effects of age, period, and cohort. Prior knowledge is combined with observed data to derive posterior distributions. The outstanding predictive performance of the BAPC model has been validated previously (19, 20).

The slope index and concentration index were used to analyze health inequalities based on the SDI. The slope index was calculated by regressing ASR on a table of the relative position of the SDI, defined by the midpoint of the cumulative range of population classes ranked by the SDI, and was used to quantify inequalities associated with the burden in different countries and territories (21). The concentration index for the health inequality analysis was calculated by fitting a Lorenz concentration curve to the observed cumulative relative distribution of the population ranked by the SDI and the burden and numerically integrating the area under the curve (21).

All rates were calculated per 100,000 WCBA. All the above statistical analyses and graphs were performed in R 4.3.2 with a test level of *α* = 0.05.

## 3 Results

### 3.1 Overview of the global burden

Among WCBA globally, the number of prevalent cases of diabetes have increased from 24.07 million in 1990 to 79.04 million in 2021, with the ASPR increasing from 1,960.8 to 3,942.2 per 100,000 (Table 1). In 2021, the global incidence of diabetes in WCBA was 5.42 million. Additionally, diabetes has led to 52.8 thousand deaths in WCBA globally, resulting in 7.82 million DALYs (Supplementary Tables S1, S2, and Table 2). Type 2 diabetes was the most common type of diabetes in 1990 and 2021, accounting for more than 87% and 93% of cases, respectively.

**Table 1:** Prevalence of diabetes in women of childbearing age in 1990 and 2021 for all locations, with EAPC from 1990 to 2021.

Location	Case no. in 1990 (95% UI)	ASR in 1990 (95% UI)	Case no. in 2021 (95% UI)	ASR in 2021 (95% UI)	EAPC (95% CI) (%)
Global	24,070,080 (20,32,7828–28,205,431)	1,960.8 (1,663.36–2,289.03)	79,036,087 (68,506,492–90,496,956)	3,942.2 (3,413.43–4,517.69)	2.25 (2.18–2.33)
Type					
Type 1 diabetes	2,998,484 (3,675,629–2,435,335)	228.9 (186.65–279.59)	5,177,165 (4,155,502–6,429,620)	263.6 (211.25–327.80)	0.47 (0.45–0.49)
Type 2 diabetes	21,071,595 (25,321,261–17,275,462)	1,731.9 (1,430.13–2,069.14)	73,858,922 (63,433,476–85,320,524)	3,678.6 (3,154.75–4,254.46)	2.43 (2.35–2.52)
SDI					
High SDI	3,763,061 (3,240,036–4,323,218)	1,604.2 (1,380.94–1,843.77)	10,739,817 (9,471,023–12,093,535)	3,936.9 (3,458.41–4,447.63)	2.93 (2.87–3.00)
High-middle SDI	4,897,966 (4,045,178–5,848,336)	1,854.6 (1,537.73–2,207.97)	13,769,729 (11,860,111–15,909,803)	3,974.1 (3,402.84–4,615.56)	2.54 (2.37–2.70)
Middle SDI	8,883,371 (7,434,955–10,484,940)	2,268.8 (1,911.15–2,662.10)	26,606,579 (23,120,408–30,463,481)	4,046.8 (3,508.12–4,641.85)	1.82 (1.70–1.93)
Low-middle SDI	4,827,652 (4,096,539–5,610,242)	2,020.0 (1,722.78–2,336.43)	19,818,635 (16,967,041–22,976,711)	4,107.0 (3,522.89–4,752.79)	2.25 (2.21–2.28)
Low SDI	1,674,182 (1,416,933–1,954,280)	1,736.5 (1,477.10–2,016.03)	8,033,345 (6,879,915–9,325,248)	3,360.2 (2,891.78–3,882.59)	2.09 (2.06–2.12)
Region					
Andean Latin America	105,253 (89,458–122,384)	1,302.6 (1,114.05–1,507.12)	417,717 (361,891–477,226)	2,406.2 (2,085.30–2,748.12)	2.10 (1.99–2.20)
Australasia	61,314 (53,314–70,561)	1,124.3 (978.66–1,293.07)	155,387 (133,430–179,379)	1,950.2 (1,673.56–2,252.88)	1.91 (1.86–1.96)
Caribbean	272,822 (237,244–312,517)	3,281.1 (2,864.92–3,745.42)	776,984 (670,140–893,068)	6,321.7 (5,447.78–7,269.85)	2.08 (2.05–2.12)
Central Asia	223,870 (191,927–257,448)	1,555.6 (1,344.49–1,778.22)	884,894 (778,149–1,006,774)	3,512.2 (3,087.13–3,996.52)	2.75 (2.66–2.84)
Central Europe	396,852 (336,556–461,676)	1,229.5 (1,043.87–1,430.01)	600,198 (508,899–703,926)	1,812.4 (1,529.19–2,132.99)	1.20 (1.17–1.22)
Central Latin America	1,358,128 (1,159,231–1,568,201)	3,845.1 (3,301.75–4,413.46)	4,057,038 (3,493,808–4,662,167)	5,848.5 (5,033.22–6,724.26)	1.32 (1.22–1.43)
Central Sub-Saharan Africa	170,080 (142,986–200,999)	1,598.9 (1,347.85–1,879.34)	953,754 (805,628–1,121,443)	3,319.8 (2,818.95–3,879.64)	2.31 (2.28–2.34)
East Asia	7,058,865 (5,714,738–8,629,238)	2,342.5 (1,908.42–2,849.58)	18,236,784 (15,600,348–21,224,510)	4,925.8 (4,181.83–5,767.59)	2.53 (2.25–2.82)
Eastern Europe	655,047 (548,078–767,378)	1,149.6 (962.76–1,347.32)	1,243,692 (1,045,113–1,460,386)	2,066.4 (1,729.77–2,433.63)	1.74 (1.66–1.82)
Eastern Sub-Saharan Africa	432,413 (366,201–504,181)	1,168.3 (996.21–1,353.09)	1,712,894 (1,454,866–2,000,257)	1,830.0 (1,561.90–2,125.15)	1.29 (1.22–1.35)
High-income Asia Pacific	825,163 (700,218–957,693)	1,716.6 (1,453.67–1,995.85)	1,837,477 (1,591,579–2,099,372)	4,088.6 (3,515.72–4,702.37)	2.90 (2.77–3.03)
High-income North America	1,231,383 (1,059,061–1,418,556)	1,606.2 (1,381.98–1,850.54)	3,711,241 (3,303,531–4,160,553)	4,071.8 (3,619.94–4,570.68)	3.08 (3.01–3.14)
North Africa and Middle East	1,286,375 (1,099,035–1,487,432)	1,966.4 (1,690.65–2,262.69)	8,369,093 (7,238,507–9,597,075)	5,228.2 (4,523.88–5,994.53)	3.17 (3.12–3.23)
Oceania	55,467 (47,507–64,349)	4,134.0 (3,561.23–4,774.30)	294,718 (253,122–340,912)	8,991.4 (7,740.54–10,383.00)	2.46 (2.43–2.49)
South Asia	5,103,367 (4,280,583–5,994,753)	2,242.0 (1,887.56–2,623.24)	21,093,035 (17,887,751–24,636,915)	4,418.1 (3,752.96–5,152.77)	2.16 (2.14–2.19)
Southeast Asia	1,653,213 (1,412,575–1,917,029)	1,584.4 (1,359.75–1,831.69)	4,982,110 (4,280,086–5,730,242)	2,606.8 (2,236.74–3,001.23)	0.85 (0.58–1.12)
Southern Latin America	137,229 (117,075–160,469)	1,149.3 (981.25–1,343.05)	462,073 (388,496–537,685)	2,491.7 (2,092.83–2,902.66)	2.66 (2.61–2.70)
Southern Sub-Saharan Africa	213,316 (178,841–251,974)	1,862.9 (1,569.05–2,189.29)	604,669 (508,907–709,584)	2,842.3 (2,393.75–3,334.43)	1.26 (1.17–1.34)
Tropical Latin America	707,003 (596,420–828,553)	2,025.5 (1,714.28–2,370.53)	1,793,481 (1,509,844–2,115,174)	2,744.9 (2,306.6–3,241.32)	0.96 (0.89–1.02)
Western Europe	1,541,049 (1,310,724–1,780,185)	1,557.8 (1,324.12–1,800.94)	3,811,261 (3,271,007–4,388,769)	3,675.5 (3,137.42–4,253.70)	2.79 (2.72–2.85)
Western Sub-Saharan Africa	581,870 (493,279–680,427)	1,566.5 (1,336.91–1,819.14)	3,037,585 (2,587,288–3,535,722)	2,885.0 (2,472.25–3,339.26)	2.02 (1.97–2.06)

Note: ASR is per 100,000 women of childbearing age.

EAPC, estimated annual percentage change; ASR, age-standardized rate; UI, uncertainty interval; CI, confidence interval; SDI, sociodemographic index.

**Table 2:** Disability-adjusted life years of diabetes in women of childbearing age in 1990 and 2021 for all locations, with EAPC from 1990 to 2021.

Location	Case no. in 1990 (95% UI)	ASR in 1990 (95% UI)	Case no. in 2021 (95% UI)	ASR in 2021 (95% UI)	EAPC (95% CI) (%)
Global	3,149,985 (2,613,450–3,838,272)	260.8 (216.66–317.01)	7,819,152 (6,057,024–9,986,942)	388.4 (300.74–496.26)	1.16 (1.09–1.24)
Type					
Type 1 diabetes	692,471 (577,343–880,064)	53.2 (44.31–67.67)	923,570 (758,476–1,112,643)	47.1 (38.70–56.71)	−0.59 (−0.67–0.5)
Type 2 diabetes	2,457,514 (1,974,006–3,065,109)	207.6 (167.68–257.59)	6,895,581 (5,237,271–8,947,854)	341.3 (258.87–443.27)	1.51 (1.44–1.58)
SDI					
High SDI	410,724 (328,824–509,197)	175.0 (140.07–216.96)	821,882 (593,750–1,100,330)	296.4 (213.84–397.85)	1.62 (1.52–1.71)
High-middle SDI	505,325 (395,939–648,833)	193.9 (152.19–248.55)	1,050,038 (748,251–1,433,226)	297.1 (211.00–406.75)	1.20 (1.07–1.33)
Middle SDI	1,121,150 (917,579–1,379,026)	294.8 (241.90–361.22)	2,664,574 (2,065,063–3,395,923)	400.7 (309.98–511.44)	0.82 (0.73–0.91)
Low-middle SDI	775,464 (648,475–922,082)	330.2 (276.46–391.48)	2,254,635 (1,787,895–2,828,751)	472.3 (374.87–592.10)	1.08 (1.03–1.12)
Low SDI	333,000 (279,352–397,146)	357.8 (301.58–425.70)	1,019,186 (821,521–1,276,044)	438.4 (354.48–548.76)	0.47 (0.40–0.55)
Region					
Andean Latin America	17,562 (14,299–21,544)	224.8 (183.24–275.63)	50,299 (39,392–64,853)	291.0 (227.94–375.16)	0.72 (0.63–0.80)
Australasia	6,365 (4,944–8,124)	116.9 (90.90–149.27)	12,743 (9,065–17,493)	157.8 (112.21–216.51)	0.95 (0.87–1.03)
Caribbean	52,953 (43,883–65,249)	642.1 (532.85–791.37)	108,635 (82,912–142,338)	883.0 (673.71–1,156.64)	0.96 (0.86–1.06)
Central Asia	29,533 (24,015–36,658)	207.1 (167.13–258.73)	92,245 (69,502–122,026)	369.4 (278.76–488.16)	1.48 (1.24–1.72)
Central Europe	55,552 (44,632–69,180)	174.0 (140.14–216.35)	59,415 (42,991–80,256)	179.4 (130.84–240.92)	0.1 (−0.03–0.23)
Central Latin America	199,705 (165,114–241,900)	592.8 (491.29–715.62)	521,329 (409,569–660,162)	747.9 (587.37–947.33)	0.6 (0.45–0.75)
Central Sub-Saharan Africa	36,700 (27,319–48,317)	366.9 (273.84–484.29)	133,436 (100,454–176,241)	485.3 (367.05–641.25)	0.85 (0.81–0.90)
East Asia	612,545 (455,963–813,509)	207.8 (155.41–274.78)	1,202,958 (817,107–1,679,524)	317.9 (214.19–446.70)	1.28 (1.05–1.51)
Eastern Europe	80,548 (65,046–100,089)	144.0 (116.63–178.57)	127,616 (97,262–166,183)	216.4 (166.54–279.77)	0.48 (0.18–0.78)
Eastern Sub-Saharan Africa	137,334 (113,592–165,456)	400.7 (333.02–482.29)	311,237 (254,166–379,518)	349.2 (286.14–424.92)	−0.78 (−0.92–0.65)
High-income Asia Pacific	81,624 (62,227–104,778)	169.2 (128.99–217.25)	131,519 (89,290–187,184)	282.4 (191.22–402.28)	1.77 (1.57–1.98)
High-income North America	159,821 (134,691–191,663)	207.7 (174.93–249.25)	312,738 (238,639–407,230)	340.6 (259.86–443.78)	1.49 (1.39–1.59)
North Africa and Middle East	175,771 (142,896–217,865)	274.1 (222.69–339.84)	777,302 (576,737–1,035,756)	487.0 (361.45–649.10)	1.99 (1.88–2.09)
Oceania	12,362 (9,294–16,228)	995.7 (753.88–1,304.03)	44,321 (34,494–56,414)	1,385.6 (1,080.96–1,760.14)	1.01 (0.96–1.07)
South Asia	689,606 (565,319–833,659)	307.8 (252.66–370.66)	2,149,267 (1,664,271–2,738,356)	454.9 (352.83–578.88)	1.18 (1.10–1.26)
Southeast Asia	371,392 (311,930–445,678)	365.2 (307.16–437.62)	769,820 (636,365–938,210)	400.8 (331.15–488.51)	−0.17 (−0.32–0.03)
Southern Latin America	20,435 (16,960–24,725)	170.9 (141.79–206.95)	41,261 (30,359–55,324)	221.8 (163.33–297.28)	0.92 (0.82–1.03)
Southern Sub-Saharan Africa	42,554 (36,275–50,571)	395.6 (338.28–468.40)	98,606 (81,077–119,923)	477.3 (393.23–579.13)	1.21 (0.83–1.60)
Tropical Latin America	130,001 (111,857–151,800)	376.7 (323.38–440.36)	244,008 (195,855–303,819)	372.4 (299.31–463.34)	−0.2 (−0.35–0.04)
Western Europe	137,444 (103,751–178,915)	138.4 (104.49–180.34)	254,897 (171,196–359,075)	240.3 (160.71–339.60)	1.81 (1.71–1.90)
Western Sub-Saharan Africa	100,177 (79,788–122,574)	285.2 (227.92–348.34)	375,500 (293,103–481,939)	370.9 (290.49–475.00)	0.79 (0.71–0.88)

Note: ASR is per 100,000 women of childbearing age.

EAPC, estimated annual percentage change; ASR, age-standardized rate; UI, uncertainty interval; CI, confidence interval; SDI, sociodemographic index.

The burden of diabetes varies among WCBA in different regions. In 2021, among the five SDI regions, the low-middle SDI region had the highest ASPR and ASDR, at 4,107.0 and 472.3 per 100,000, respectively. In comparison, the low SDI region had the lowest ASPR at 3,360.2 per 100,000 and high SDI region had the lowest ASDR at 296.4 per 100,000. Notably, in 2021, although the low SDI region had the lowest ASPR and ASIR, its ASDR and ASMR were ranked second and first, at 438.4 and 4.1 per 100,000, respectively. Oceania, Caribbean, and Central Latin America had the highest ASPR of diabetes in WCBA, with rates of 8,991.4, 6,321.7 and 5,848.5 per 100,000, respectively. In contrast, Central Europe, Eastern Sub-Saharan Africa, and Australasia had the lowest ASPR of diabetes in WCBA, with rates of 1,812.4, 1,830.0 and 1,950.2 per 100,000, respectively. More detailed information is provided in Tables 1, 2, Supplementary Tables S1, S2.

**Figure 1:**
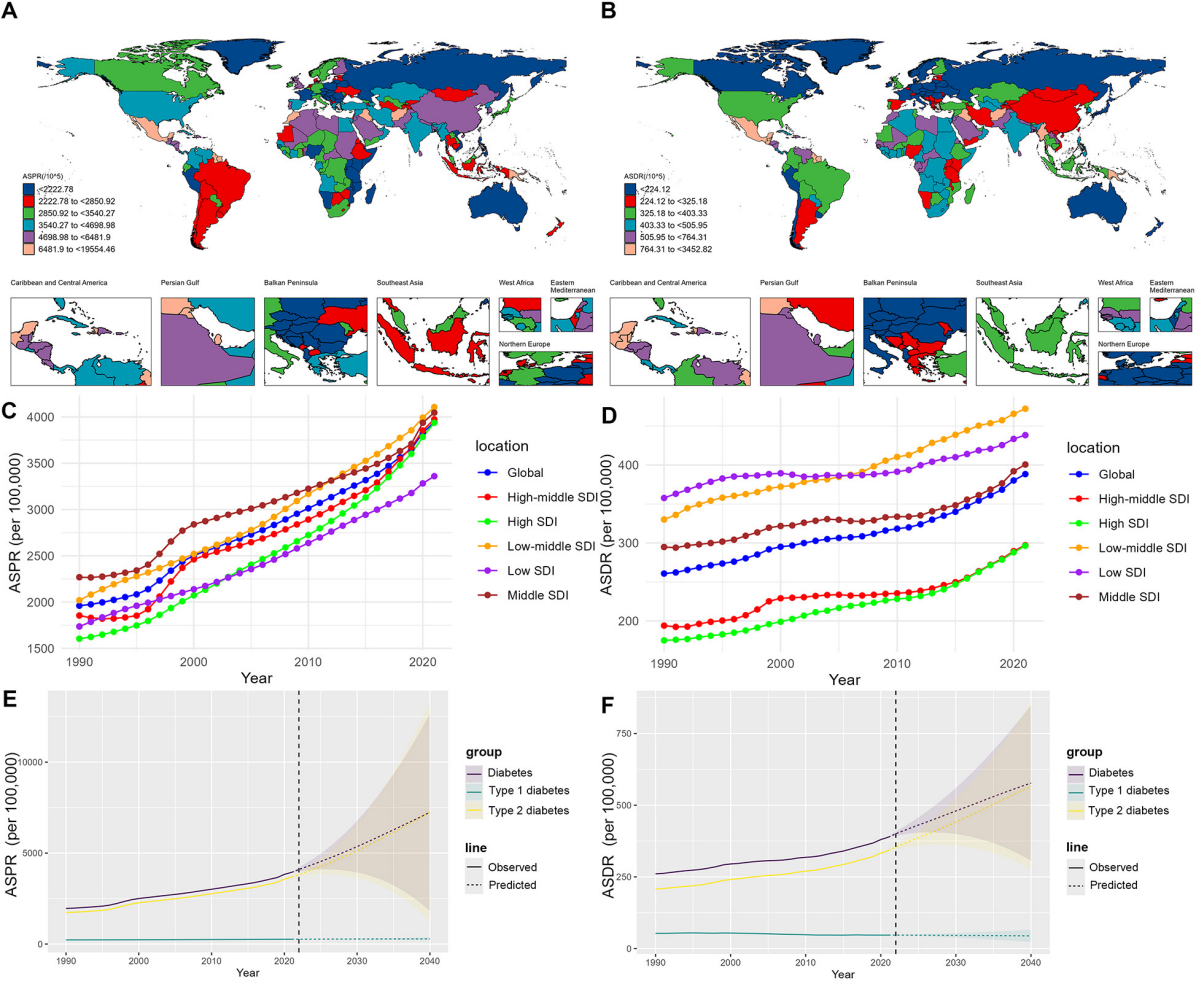
**(A,B)** ASPRs and ASDRs of diabetes in women of childbearing age in 204 countries or territories in 2021. **(C,D)** Global and regional trends in ASPRs and ASDRs of diabetes in women of childbearing age, 1990–2021. **(E,F)** Prediction of the global ASPRs and ASDRs of diabetes in women of childbearing age, 2022–2040. ASPR, age-standardized prevalence rate; ASDR, age-standardized DALY rate; DALY, disability-adjusted life year.

Figure 1, Supplementary Figure S1, and Tables S3–S6 show the burden of diabetes in WCBA at the national level. In 2021, American Samoa had the highest ASPR at 17,264.6 per 100,000, and Marshall Islands had the highest ASDR, ASIR, and ASMR at 3,452.8, 1,377.9, and 44.7 per 100,000, respectively.

### 3.2 Trend analysis and prediction

From 1990 to 2021, the global ASPR of diabetes among WCBA showed an increasing trend, with an EAPC of 2.25%. Among them, the ASPR of type 2 diabetes cases increased more significantly, with an EAPC of 2.43%. In contrast, the ASDR for type 1 diabetes cases tended to decrease, with an EAPC of −0.59%, whereas the ASDR for type 2 diabetes cases tended to increase, with an EAPC of 1.51%. In the high SDI region, the ASPR and ASDR increased the most, with EAPCs of 2.93% and 1.62%, respectively (Tables 1, 2, and Figure 1). The BAPC prediction analysis indicated that from 2022 to 2040, the ASPR and ASDR for diabetes among WCBA globally will continue to rise, with a notable upward trend for type 2 diabetes and a tendency toward stabilization for type 1 diabetes (Figure 1). In 2040, the ASPR of diabetes among WCBA globally may exceed 7,000 per 100,000 (Figure 1). From 1990 to 2021, the global ASIR of diabetes among WCBA showed an increasing trend, with an EAPC of 1.92% (Supplementary Table S1 and Figure S2). This increasing trend is predicted to continue until 2040 (Supplementary Figure S3). In contrast, the ASMR tends to decrease and stabilize (Supplementary Table S2, and Figures S2, S3).

### 3.3 Health inequality analysis

This study revealed that the burden of diabetes varies in WCBA in different SDI regions and in different countries. To further validate the relationship between the burden of diabetes and socioeconomic level in WCBA, this study conducted an SDI-related health inequality analysis.

**Figure 2:**
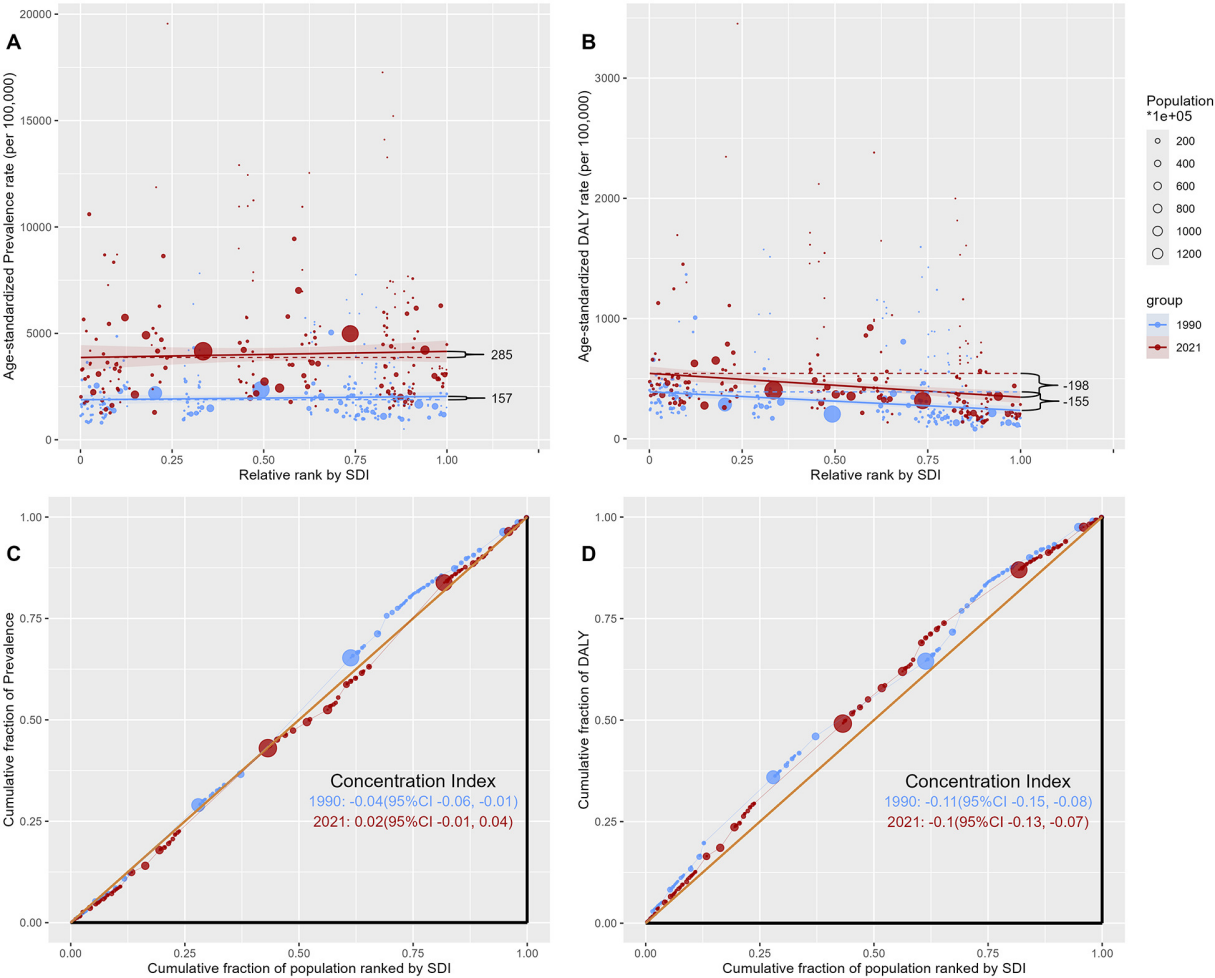
Health inequalities analysis of diabetes in women of childbearing age in 204 countries or territories, 1990 and 2021. **(A)** Slope index of age-standardized prevalence rates. **(B)** Slope index of age-standardized DALY rates. **(C)** Concentration index of age-standardized prevalence rates. **(D)** Concentration index of age-standardized DALY rates. DALY, disability-adjusted life year.

The slope index revealed that the SDI was significantly positively associated with the ASPR of diabetes in WCBA in 1990, and this positive association increased in 2021 (ASPR in 1990: 157 to ASPR in 2021: 285) (Figure 2A). In contrast, the SDI was significantly negatively associated with the ASDR of diabetes in WCBA in 1990, and this negative association increased in 2021 (ASDR in 1990: −155 to ASDR in 2021: −198) (Figure 2B). In addition, the SDI was positively associated with ASIR of diabetes in WCBA and the SDI was negatively associated with ASMR from diabetes in WCBA in 1990 and 2021 (ASIR in 1990: 30 to ASIR in 2021: 55; ASMR in 1990: −3 to ASMR in 2021: −4) (Supplementary Figure S4).

The concentration index revealed that the prevalence of diabetes in WCBA in 1990 was concentrated in low SDI countries, whereas the prevalence of diabetes in WCBA in 2021 was concentrated in high SDI countries (prevalence in 1990: −0.04 to prevalence in 2021: 0.02) (Figure 2C). The DALY of diabetes in WCBA in 1990 and 2021 was concentrated in low SDI countries (DALY in 1990: −0.11 to DALY in 2021: 0.10) (Figure 2D). In addition, the incidence of diabetes in WCBA in 1990 was concentrated in low SDI countries, whereas the incidence of diabetes in WCBA in 2021 was concentrated in high SDI countries (incidence in 1990: −0.11 to incidence in 2021: 0.10) (Supplementary Figure S5). The deaths associated with diabetes in WCBA in 1990 and 2021 were concentrated in low SDI countries (deaths in 1990: −0.19 to deaths in 2021: −0.26) (Supplementary Figure S5).

## 4 Discussion

Diabetes has long been a global public health concern, and for WCBA, it can have long-term adverse effects on both individuals and their offspring. However, there is currently a lack of systematic analyses on the burden of diabetes among WCBA. This study, which is based on GBD 2021 data, provides a comprehensive analysis of the burden of diabetes among WCBA globally since 1990. This study indicates that over the past three decades, the global burden of diabetes among WCBA has been on an overall upward trend, and it is predicted to continue to increase through 2040. In 2021, 79.04 million WCBA were living with diabetes globally, resulting in 7.82 million DALYs. Prevalent cases of diabetes in WCBA are concentrated in high SDI countries or regions, and DALYs are concentrated in low SDI countries or regions. Policymakers should emphasize the health management of WCBA, especially those planning to become pregnant, to reduce the burden of diabetes on the health of this population.

Representing almost a quarter of the global population, the health of WCBA is intertwined with the well-being of their offspring. Diabetes is a serious threat to the health of WCBA (5, 6), and having the most up-to-date epidemiological information on diabetes is beneficial to the State and the Government in formulating public health policies to prevent and reduce the burden caused by diabetes on WCBA. This study revealed that the ASPR and ASDR for diabetes in WCBA increased globally from 1990 to 2021 and will continue to increase through 2040. Obesity or high body mass index is the most important risk factor for type 2 diabetes. The present study found that obesity-related DALYs rose 0.48% annually from 2000 to 2019 and are predicted to increase by 39.8% from 2020 to 2030 (22). Compared with men, women experience a lower rate of DALYs due to obesity, and the rate of increase is lower (22). Controlling and reducing the proportion of the population that is obese remains an effective measure to reduce the burden of diabetes in WCBA.

Exposure to ambient particulate matter is associated with an increased risk of type 2 diabetes. This association is attributed to elevated oxidative stress, systemic inflammation, impaired endothelial function, and mitochondrial dysfunction caused by particulate matter exposure (23, 24). A study from China found that long-term exposure to fine particulate matter increases the risk of type 2 diabetes in WCBA, with an 18% increase in the risk of diabetes for every quartile increase in 3-year average fine particulate matter concentrations (25). Another study showed that the global burden of type 2 diabetes due to fine particulate matter exposure has increased over the past three decades, especially in less economically developed regions (26). Particulate matter pollution can be reduced by reducing coal use, installing abatement exhaust filters, and strengthening motor vehicle emission standards (27); however, the global spread of these measures remains a significant challenge.

Smoking is an identified risk factor for type 2 diabetes (28), and global smoking rates have been effectively controlled in recent years, with a 34.4% decline in women's smoking rates globally from 1990 to 2015 (29). Nonetheless, a significant amount of type 2 diabetes is still attributable to smoking (11); therefore, the continuation of smoking prevention programs globally is beneficial for reducing the incidence of type 2 diabetes in WCBA. Moreover, engaging in regular physical activity and adopting a healthy dietary regimen can help reduce the disease burden associated with both type 1 and type 2 diabetes (30–32). In the future, the development of effective intervention programs and popular science education may alleviate this increasing burden.

This study revealed that the ASPR and the ASIR of type 2 diabetes cases have increased more dramatically than those of type 1 diabetes cases. A previous study found that the disease burden of type 2 diabetes in adolescents and young adults has increased globally over the last three decades (33). In other words, individuals with type 2 diabetes are becoming younger, and the increasing number of prevalent cases of early-onset type 2 diabetes will change the global epidemiological pattern of diabetes. For WCBA, there are more controllable risk factors for type 2 diabetes, and changing poor lifestyle habits increases the likelihood of reducing the burden of type 2 diabetes (34, 35). In fact, however, the continuing increase in the burden of type 2 diabetes in WCBA appears to be unstoppable. Notably, despite the increase in the ASPR and the ASIR of type 1 diabetes cases, both the ASDR and the ASMR have decreased. The efforts of healthcare and public health management in this area have yielded positive results in improving the condition of individuals with type 1 diabetes (36). An increasing number of WCBA with type 1 diabetes are experiencing unprecedented improvements in survival and quality of life. This means that there is growing global pressure to manage type 1 diabetes in WCBA. Traditionally, type 1 diabetes has been thought of as a disease that occurs in childhood and adolescence and can have a significant impact on life expectancy (37). Almost all existing clinical practices and guidelines need to be refined to target the management of type 1 diabetes in WCBA.

The International Diabetes Federation's estimates for the global prevalence of diabetes in 2021 indicate that the rate of diabetes is higher in high-income countries than in low-income countries (2). The findings of this study reveal that the ASPR of diabetes among WCBA in high SDI countries or regions exceeds that of low SDI countries or regions. The prevalent cases of diabetes among WCBA are more concentrated in high SDI countries or regions, which may be associated with dietary habits that vary across nations. Western diets, characterized by increased caloric intake and the consumption of processed foods, are linked to weight gain and insulin resistance (38). Furthermore, sedentary lifestyles, which are common in urbanized societies, lead to reduced physical activity, which is another critical factor in the development of diabetes (39). In addition, healthcare systems in high-income countries are more likely to diagnose diabetes because they have better access to healthcare services and diagnostic tools. This increased diagnostic capacity can lead to higher reported rates of diabetes, as opposed to underdiagnosis in low-income countries where healthcare resources may be limited (40, 41).

This study revealed that WCBA with type 1 diabetes are concentrated in countries with higher SDI, while type 1 diabetes—related disability and mortality are concentrated in countries with lower SDI. For WCBA with type 2 diabetes, no concentration related to SDI is observed, but type 2 diabetes—related disability and mortality are also concentrated in countries with lower SDI. Moreover, this health inequality is worsening. Pronounced disparities persist between low- and high-income countries in diabetes awareness, prevention, diagnosis, and treatment, driven significantly by inequitable access to and underutilization of modern management technologies, such as insulin delivery systems and continuous glucose monitoring (42, 43). The total global spending on diabetes among people aged 20–79 years was reported to be $966 billion in 2021, with North America and Caribbean accounting for 42.9% of the total global spending on diabetes, and Southeast Asia accounting for only 1.0%, even though the number of people living with diabetes in Southeast Asia is almost twice as high as that in North America and Caribbean (2). This means that in high-income regions such as North America, WCBA with diabetes have far greater access to health care and treatment than do those in low-income regions, which can significantly reduce the likelihood of adverse outcomes in these populations. In the future, low-income countries and regions should consider increasing funding for diabetes care for WCBA to reduce the growing burden of diabetes in this population.

Pregnant women with diabetes or hyperglycemia may be at increased risk for pregnancy complications and adverse birth outcomes, such as gestational hypertension, preeclampsia, miscarriage, preterm labor, birth defects, and fetal macrosomia (6, 7). Therefore, for those WCBA with diabetes, professional preconception counseling is essential before they plan to become pregnant. The American Diabetes Association recommends that preconception counseling should emphasize the importance of keeping blood glucose levels close to normal as safe, ideally with an A1C<6.5% (<48 mmol/mol), to reduce the risk of adverse outcomes occurring in mothers and children (8). Previous studies have shown that prenatal care interferes with perinatal outcomes and is able to reduce the risks of complications associated with this comorbidity through early intervention, especially when nutritional therapy is an integral part of this assistance (10). However, another survey found that WCBA are poorly informed about diabetes prevention services. Moreover, the low clinical response to prediabetes among WCBA results in missed opportunities for diabetes prevention in this vulnerable population (7). The health of WCBA is critical to the achievement of sustainable development goals, and in the context of the growing global burden of diabetes among WCBA, it is urgent to achieve universal access to targeted health services and health education.

This study provides the latest epidemiological information on diabetes among WCBA. To our knowledge, this is the first study to predict future trends in the global diabetes burden among WCBA and the first to assess health inequalities in the diabetes burden among WCBA related to the SDI. The findings of this study will raise public awareness of diabetes among WCBA, offer targeted recommendations for public health policies and interventions, and guide future research.

There are several limitations to this study. First, although the GBD 2021 has made every effort to gather all available data sources and has applied optimized statistical models to reduce the impact of data heterogeneity on estimates to the greatest extent possible, data heterogeneity is still a factor that cannot be overlooked. Second, due to data limitations, this study was unable to obtain provincial-level data from large countries such as the United States and China. For these nations, future research on provincial-level burden of diabetes in WCBA will be instrumental in enhancing the epidemiological information available. Third, gestational diabetes is a significant type of diabetes that poses a threat to pregnant women and their offspring. However, in the GBD, the disease burden attributable to gestational diabetes is included under “Other direct maternal disorders”, and this study was unable to obtain data related to gestational diabetes or its reproductive outcomes for analysis.

## 5 Conclusion

The global burden of diabetes among WCBA has increased over the past three decades. This burden is concentrated in low and middle SDI countries or regions. Until further effective measures are taken, this burden will continue to increase in the future. Therefore, diabetes care policies for WCBA urgently need to be improved and popularized.

## Data Availability

Publicly available datasets were analyzed in this study. This data can be found here: https://vizhub.healthdata.org/gbd-results/.
