# Supplementary material for: Global, regional, and national burden of diabetes in women of childbearing age, 1990–2021: a systematic analysis from the global burden of disease study 2021
Source: Front Glob Womens Health. 2025 Jul 15;6:1528661. doi: 10.3389/fgwh.2025.1528661 (PMC12303903; doi:10.3389/fgwh.2025.1528661)
Supplement: Supplementary file 1 [file Table1.docx]

**Supplementary Table 1.** Incidence of diabetes in women of childbearing age in 1990 and 2021 for all locations, with EAPC from 1990 to 2021.

| Location | Case no. in 1990 (95% UI) | ASR in 1990 (95% UI) | Case no. in 2021 (95% UI) | ASR in 2021 (95% UI) | EAPC (95% CI) (%) |
| --- | --- | --- | --- | --- | --- |
| **Global** | 1827157 (1322100-2449984) | 146.5 (106.38-196.23) | 5424222 (4059309-7017752) | 273.4 (204.5-353.53) | 1.92 (1.88-1.96) |
| **Type** |  |  |  |  |  |
| Type 1 diabetes | 59580 (28486-106731) | 4.3 (2.07-7.76) | 106455 (48430-195362) | 5.5 (2.51-10.13) | 0.79 (0.73-0.84) |
| Type 2 diabetes | 1767577 (1256520-2392378) | 142.1 (101.61-192.05) | 5317767 (3951143-6923346) | 267.8 (198.9-348.61) | 1.95 (1.91-1.99) |
| **SDI** |  |  |  |  |  |
| High SDI | 295142 (220745-384762) | 127.2 (95-166.02) | 738986 (566788-940128) | 279.2 (213.33-355.42) | 2.56 (2.49-2.63) |
| High-middle SDI | 370224 (256066-512235) | 140 (97.14-193.26) | 815274 (593584-1080554) | 255.5 (186.45-337.64) | 1.87 (1.8-1.94) |
| Middle SDI | 686143 (486463-929881) | 170.1 (121.15-230.56) | 1832487 (1364010-2378620) | 285.8 (212.77-370.38) | 1.47 (1.41-1.53) |
| Low-middle SDI | 356678 (262032-468101) | 144.2 (106.55-188.91) | 1469872 (1092799-1899985) | 300.4 (223.94-387.99) | 2.31 (2.27-2.34) |
| Low SDI | 116931 (84357-154467) | 116.7 (84.62-153.72) | 562047 (411461-734491) | 223.3 (163.63-291.77) | 2.05 (2.02-2.09) |
| **Region** |  |  |  |  |  |
| Andean Latin America | 8676 (6503-11162) | 106.6 (80.73-135.81) | 35381 (26794-44619) | 204.2 (154.68-257.38) | 2.19 (2.1-2.29) |
| Australasia | 4664 (3420-6070) | 85.8 (62.84-111.86) | 11275 (8109-14910) | 143.6 (103.4-190.09) | 1.77 (1.73-1.81) |
| Caribbean | 22583 (17290-28099) | 270.3 (208.77-334.02) | 62048 (46606-78669) | 506.9 (380.59-642.52) | 2.01 (1.97-2.04) |
| Central Asia | 16866 (12440-21818) | 114.1 (84.86-147.05) | 68633 (52353-86700) | 275.2 (209.78-347.69) | 2.98 (2.86-3.1) |
| Central Europe | 40564 (30938-50956) | 126 (95.99-158.51) | 61470 (47144-78043) | 191 (145.56-243.28) | 1.33 (1.29-1.37) |
| Central Latin America | 107962 (80150-140560) | 295.2 (221.14-383) | 308017 (231112-394355) | 445.8 (334.2-571) | 1.17 (1.07-1.26) |
| Central Sub-Saharan Africa | 11539 (8230-15429) | 103.1 (73.69-137.4) | 63681 (45592-83852) | 208.6 (149.58-274.06) | 2.27 (2.24-2.3) |
| East Asia | 525579 (344793-749187) | 170.2 (111.34-242.88) | 889938 (612406-1221173) | 280 (197.19-376.99) | 1.44 (1.31-1.57) |
| Eastern Europe | 48742 (34859-65202) | 85.9 (61.43-115.18) | 100119 (72016-132953) | 176.2 (126.67-233.95) | 2.19 (2.11-2.27) |
| Eastern Sub-Saharan Africa | 26996 (19270-35967) | 68.9 (49.32-91.73) | 107662 (77170-143099) | 107.7 (77.2-143.12) | 1.27 (1.19-1.35) |
| High-income Asia Pacific | 64795 (48066-84862) | 134.9 (99.61-177.11) | 114029 (83218-152074) | 266.9 (193.61-354.64) | 2.17 (2.05-2.29) |
| High-income North America | 111240 (82615-145539) | 146.1 (108.42-191.38) | 299692 (231052-379081) | 329.7 (253.72-417.44) | 2.71 (2.67-2.75) |
| North Africa and Middle East | 110809 (83491-142538) | 167.7 (127.57-214.54) | 746756 (573845-932735) | 468.3 (359.99-584.93) | 3.34 (3.29-3.4) |
| Oceania | 4243 (3168-5410) | 302.8 (227.49-384.07) | 22145 (16552-28008) | 662 (495.62-835.83) | 2.55 (2.52-2.58) |
| South Asia | 360699 (258764-486078) | 152.5 (109.69-205.57) | 1463444 (1056941-1935032) | 302.2 (218.47-399.57) | 2.15 (2.09-2.21) |
| Southeast Asia | 131837 (96593-171778) | 123.7 (91.02-160.94) | 419256 (319120-531343) | 221 (167.99-280.31) | 1.16 (0.91-1.41) |
| Southern Latin America | 14098 (10624-18324) | 117.9 (88.98-153.24) | 42989 (31753-55184) | 233.5 (172.14-300.09) | 2.37 (2.29-2.44) |
| Southern Sub-Saharan Africa | 15126 (10705-20403) | 127.2 (90.12-171.92) | 42715 (30114-57685) | 202.6 (142.85-273.78) | 1.41 (1.35-1.47) |
| Tropical Latin America | 64596 (47044-85992) | 186.3 (136.24-247.98) | 156825 (113480-208826) | 241.5 (174.39-322.06) | 0.81 (0.71-0.91) |
| Western Europe | 96391 (69477-128729) | 99.2 (71.39-132.51) | 208552 (150139-276801) | 219.1 (157.77-289.64) | 2.58 (2.51-2.65) |
| Western Sub-Saharan Africa | 39149 (28081-52413) | 102.1 (73.61-136.77) | 199597 (143693-265850) | 180.9 (130.46-241.14) | 1.85 (1.81-1.88) |

Note: ASR is per 100,000 women of childbearing age. EAPC, estimated annual percentage change; ASR, age-standardized rate; UI, uncertainty interval; CI, confidence interval; SDI, sociodemographic index.

**Supplementary Table 2.** Deaths of diabetes in women of childbearing age in 1990 and 2021 for all locations, with EAPC from 1990 to 2021.

| Location | Case no. in 1990 (95% UI) | ASR in 1990 (95% UI) | Case no. in 2021 (95% UI) | ASR in 2021 (95% UI) | EAPC (95% CI) (%) |
| --- | --- | --- | --- | --- | --- |
| **Global** | 31111 (28895-34516) | 2.6 (2.45-2.92) | 52768 (48236-57666) | 2.6 (2.38-2.85) | -0.26 (-0.37--0.15) |
| **Type** |  |  |  |  |  |
| Type 1 diabetes | 8951 (7428-12190) | 0.7 (0.58-0.95) | 10662 (8953-12649) | 0.5 (0.46-0.64) | -1.09 (-1.21--0.96) |
| Type 2 diabetes | 22161 (20048-24298) | 1.9 (1.76-2.12) | 42106 (37840-46438) | 2.1 (1.85-2.28) | 0.01 (-0.1-0.12) |
| **SDI** |  |  |  |  |  |
| High SDI | 3463 (3348-3591) | 1.5 (1.42-1.53) | 3020 (2831-3258) | 1.1 (1-1.15) | -1.3 (-1.47--1.14) |
| High-middle SDI | 3843 (3469-4289) | 1.5 (1.36-1.68) | 3607 (3243-4053) | 1.0 (0.89-1.11) | -1.84 (-2.06--1.63) |
| Middle SDI | 10707 (9893-11685) | 2.9 (2.7-3.19) | 18334 (16879-19923) | 2.7 (2.5-2.95) | -0.52 (-0.68--0.36) |
| Low-middle SDI | 8838 (7778-10352) | 3.9 (3.41-4.52) | 18644 (16124-21387) | 4.0 (3.42-4.54) | 0.03 (-0.02-0.08) |
| Low SDI | 4211 (3542-5027) | 4.7 (3.98-5.6) | 9088 (7649-10642) | 4.1 (3.42-4.74) | -0.74 (-0.84--0.65) |
| **Region** |  |  |  |  |  |
| Andean Latin America | 201 (167-244) | 2.7 (2.22-3.23) | 421 (322-544) | 2.4 (1.87-3.16) | -0.5 (-0.7--0.31) |
| Australasia | 46 (41-51) | 0.8 (0.75-0.94) | 51 (44-57) | 0.6 (0.54-0.7) | -1.44 (-1.7--1.19) |
| Caribbean | 594 (504-717) | 7.3 (6.23-8.8) | 870 (644-1175) | 7.1 (5.22-9.54) | -0.26 (-0.44--0.08) |
| Central Asia | 262 (244-283) | 1.9 (1.72-2) | 559 (469-658) | 2.3 (1.9-2.67) | -0.15 (-0.62-0.33) |
| Central Europe | 494 (470-521) | 1.6 (1.48-1.65) | 252 (224-281) | 0.8 (0.71-0.89) | -2.09 (-2.36--1.82) |
| Central Latin America | 1947 (1877-2019) | 6 (5.82-6.26) | 4274 (3655-4891) | 6.1 (5.22-6.98) | -0.3 (-0.76-0.16) |
| Central Sub-Saharan Africa | 478 (327-679) | 5 (3.43-7.12) | 1265 (866-1797) | 4.8 (3.29-6.83) | -0.2 (-0.26--0.14) |
| East Asia | 4040 (3238-4917) | 1.4 (1.15-1.74) | 2895 (2232-3709) | 0.7 (0.55-0.91) | -2.65 (-2.93--2.37) |
| Eastern Europe | 633 (604-664) | 1.1 (1.09-1.2) | 706 (621-798) | 1.3 (1.12-1.43) | -1.39 (-2.09--0.69) |
| Eastern Sub-Saharan Africa | 1998 (1613-2474) | 6.2 (4.98-7.61) | 3439 (2811-4166) | 4 (3.31-4.89) | -1.76 (-1.92--1.61) |
| High-income Asia Pacific | 538 (470-619) | 1.1 (0.97-1.28) | 158 (134-188) | 0.3 (0.28-0.39) | -4.02 (-4.25--3.8) |
| High-income North America | 1682 (1626-1741) | 2.2 (2.11-2.26) | 1691 (1614-1771) | 1.8 (1.74-1.91) | -0.85 (-1.11--0.6) |
| North Africa and Middle East | 1743 (1486-2103) | 2.8 (2.4-3.4) | 4235 (3463-5188) | 2.7 (2.19-3.27) | 0.06 (-0.07-0.19) |
| Oceania | 177 (124-248) | 14.9 (10.61-20.77) | 503 (376-668) | 16.1 (12.03-21.31) | 0.21 (0.11-0.32) |
| South Asia | 7067 (6147-8079) | 3.2 (2.82-3.69) | 16054 (13615-18846) | 3.4 (2.93-4.05) | 0.14 (0.06-0.22) |
| Southeast Asia | 4971 (4184-6287) | 5 (4.25-6.38) | 8312 (7016-9948) | 4.3 (3.63-5.15) | -0.76 (-0.9--0.61) |
| Southern Latin America | 227 (204-253) | 1.9 (1.71-2.12) | 226 (198-255) | 1.2 (1.06-1.37) | -1.41 (-1.6--1.21) |
| Southern Sub-Saharan Africa | 532 (458-617) | 5.2 (4.47-5.99) | 1121 (940-1321) | 5.6 (4.66-6.54) | 1.4 (0.76-2.04) |
| Tropical Latin America | 1475 (1400-1550) | 4.3 (4.12-4.57) | 2062 (1942-2185) | 3.1 (2.96-3.33) | -1.4 (-1.63--1.16) |
| Western Europe | 833 (799-871) | 0.8 (0.8-0.87) | 381 (364-398) | 0.3 (0.32-0.36) | -2.87 (-2.97--2.77) |
| Western Sub-Saharan Africa | 1172 (914-1442) | 3.5 (2.77-4.33) | 3294 (2478-4197) | 3.4 (2.57-4.34) | -0.2 (-0.35--0.06) |

Note: ASR is per 100,000 women of childbearing age. EAPC, estimated annual percentage change; ASR, age-standardized rate; UI, uncertainty interval; CI, confidence interval; SDI, sociodemographic index.

**Supplementary Table 3.** Prevalence of diabetes in women of childbearing age in 1990 and 2021 for 204 countries or territories, with EAPC from 1990 to 2021.

| Location | Case no. in 1990 (95% UI) | ASR in 1990 (95% UI) | Case no. in 2021 (95% UI) | ASR in 2021 (95% UI) | EAPC (95% CI) (%) |
| --- | --- | --- | --- | --- | --- |
| Afghanistan | 69963 (58627-82603) | 3639.1 (3062.25-4290.72) | 643399 (544858-761364) | 10601.7 (8993.41-12503.9) | 3.64 (3.53-3.76) |
| Albania | 6669 (5483-7981) | 975.6 (805.18-1165.03) | 10528 (8704-12630) | 1581.4 (1303.92-1900.32) | 1.56 (1.52-1.6) |
| Algeria | 101222 (84358-120526) | 2202.6 (1853.26-2603.16) | 685426 (583506-795988) | 5791.9 (4923.79-6734.61) | 3.06 (2.99-3.13) |
| American Samoa | 706 (593-844) | 6842.1 (5792.23-8129.88) | 2065 (1737-2449) | 17264.6 (14455.99-20559.39) | 2.92 (2.74-3.1) |
| Andorra | 241 (203-283) | 1575.9 (1327.89-1845.18) | 802 (683-930) | 3353.5 (2830.8-3925.57) | 2.49 (2.46-2.52) |
| Angola | 34884 (28939-41697) | 1752.4 (1460.92-2084.94) | 239537 (201007-285553) | 3541.7 (2987.61-4203.5) | 2.39 (2.31-2.47) |
| Antigua and Barbuda | 529 (441-624) | 3606 (3006.2-4247.77) | 1576 (1333-1861) | 5901.3 (4979.02-6981.79) | 1.53 (1.5-1.57) |
| Argentina | 92450 (78744-108383) | 1168.6 (995.36-1369.68) | 317182 (262609-373341) | 2531.4 (2095.01-2981.78) | 2.68 (2.64-2.72) |
| Armenia | 13591 (11201-16243) | 1746.7 (1441.45-2082.52) | 23652 (20000-28140) | 2837.3 (2394.13-3378.81) | 1.5 (1.33-1.66) |
| Australia | 47927 (41692-55199) | 1052.4 (916.65-1210.97) | 121712 (102104-143873) | 1818.9 (1525.41-2151.64) | 1.82 (1.71-1.93) |
| Austria | 19201 (16484-22249) | 935.6 (803.05-1084.88) | 47932 (40159-55912) | 2167.6 (1809.84-2537.81) | 2.71 (2.64-2.77) |
| Azerbaijan | 24726 (20900-28961) | 1573.5 (1340.96-1831.82) | 108954 (92255-127984) | 3709.6 (3136.62-4361.4) | 2.87 (2.78-2.96) |
| Bahamas | 2180 (1828-2559) | 3413.7 (2872.08-3994.54) | 7083 (5897-8436) | 6230.9 (5182.49-7424.44) | 1.93 (1.87-1.99) |
| Bahrain | 2388 (1966-2844) | 2614.6 (2155.86-3103.42) | 20139 (16624-23936) | 5917.5 (4881.31-7034.08) | 2.6 (2.53-2.67) |
| Bangladesh | 523134 (442690-609908) | 2547.4 (2171.83-2949.01) | 2564138 (2199154-2996148) | 5745.5 (4933.53-6708.29) | 2.66 (2.55-2.76) |
| Barbados | 2050 (1751-2381) | 3179.9 (2716.65-3690.55) | 4637 (3910-5510) | 5741.2 (4833.49-6829.24) | 1.76 (1.68-1.83) |
| Belarus | 25131 (21114-29424) | 980.7 (824.53-1146.91) | 42287 (35469-49373) | 1602.1 (1339.16-1878.99) | 1.32 (1.21-1.43) |
| Belgium | 41679 (34758-48815) | 1651.5 (1375.82-1936.33) | 95365 (80004-111463) | 3497.2 (2922.27-4103.07) | 2.38 (2.34-2.42) |
| Belize | 998 (838-1180) | 3027.8 (2548.45-3572.26) | 6509 (5497-7692) | 5791 (4896.49-6839.34) | 2.11 (2-2.21) |
| Benin | 18089 (15277-21512) | 1893.3 (1605.88-2237.43) | 135215 (114793-159128) | 4724.3 (4040.35-5540.15) | 2.92 (2.78-3.07) |
| Bermuda | 368 (311-432) | 2055.6 (1736.97-2416.76) | 622 (526-727) | 3891.7 (3281.87-4561.32) | 2 (1.93-2.06) |
| Bhutan | 2373 (1969-2816) | 2072 (1733.02-2440.48) | 7193 (6095-8448) | 3615.4 (3069.3-4241.04) | 1.77 (1.66-1.87) |
| Bolivia (Plurinational State of) | 19851 (16634-23440) | 1511.8 (1271.73-1780.68) | 81430 (68326-97121) | 2730 (2292.42-3254.41) | 1.86 (1.81-1.91) |
| Bosnia and Herzegovina | 12561 (10320-15249) | 1124.8 (925.58-1364.59) | 19356 (15612-23528) | 2125 (1710.42-2589.27) | 2.14 (2.07-2.22) |
| Botswana | 3343 (2776-3986) | 1258.2 (1049-1494.23) | 16601 (14112-19421) | 2482.6 (2111.82-2902.91) | 2.23 (2.21-2.26) |
| Brazil | 692219 (583480-811591) | 2029.7 (1716.27-2376.47) | 1738484 (1460720-2053373) | 2736.2 (2294.62-3235.94) | 0.94 (0.87-1.01) |
| Brunei Darussalam | 1459 (1232-1717) | 2424.2 (2050.23-2846.37) | 9197 (7703-11000) | 6983 (5842.3-8356.11) | 3.57 (3.39-3.74) |
| Bulgaria | 30743 (25365-36390) | 1333.2 (1097.48-1580.47) | 40607 (34172-47894) | 2146 (1794.82-2548.1) | 1.58 (1.53-1.62) |
| Burkina Faso | 22231 (18826-26134) | 1203.5 (1022.65-1408.98) | 137334 (116277-163249) | 2862.3 (2439.59-3374.71) | 2.85 (2.75-2.94) |
| Burundi | 12785 (10737-15248) | 1165.2 (981.39-1381.7) | 49729 (41504-59006) | 1845.7 (1548.03-2181.37) | 1.36 (1.31-1.42) |
| Cabo Verde | 1362 (1147-1610) | 2007.2 (1700.62-2353) | 6075 (5128-7223) | 4156.4 (3513.25-4930.42) | 2.45 (2.28-2.62) |
| Cambodia | 25408 (21431-29807) | 1182.2 (1000.52-1381.99) | 104173 (89273-121676) | 2410.1 (2068.87-2813.06) | 2.17 (2.11-2.22) |
| Cameroon | 29687 (24955-35285) | 1442.1 (1220.55-1706.52) | 224538 (189735-265896) | 3228.4 (2744.11-3804.36) | 2.66 (2.55-2.76) |
| Canada | 78258 (70728-87269) | 1044.2 (943.86-1163.64) | 263909 (224086-309194) | 2852.2 (2429.66-3331.04) | 2.97 (2.87-3.08) |
| Central African Republic | 12968 (10742-15652) | 2332.9 (1940.84-2800.52) | 62404 (51968-74172) | 5108.5 (4276.44-6034.37) | 2.64 (2.53-2.76) |
| Chad | 18105 (15137-21459) | 1550.8 (1301.37-1827.54) | 109551 (91910-130110) | 3330.4 (2813.43-3926.21) | 2.49 (2.36-2.61) |
| Chile | 37685 (30798-46005) | 1151.9 (944.07-1402.79) | 124011 (101599-148493) | 2425.4 (1983.86-2907.69) | 2.49 (2.38-2.6) |
| China | 6844264 (5528841-8389922) | 2354.8 (1914.14-2872.46) | 17773904 (15175564-20699419) | 4986.1 (4224.96-5841.6) | 2.56 (2.27-2.86) |
| Colombia | 199528 (168801-235005) | 2645.2 (2244.19-3095.46) | 519995 (439245-611160) | 3913.6 (3304.98-4600.99) | 0.95 (0.77-1.14) |
| Comoros | 1291 (1077-1542) | 1417.5 (1190.19-1680.58) | 5288 (4459-6262) | 2848.3 (2407.41-3366.48) | 2.37 (2.29-2.45) |
| Congo | 7990 (6597-9627) | 1704.9 (1413.85-2042.32) | 46724 (38686-55602) | 3453.9 (2864.68-4100.62) | 2.27 (2.19-2.36) |
| Cook Islands | 318 (269-375) | 7758.5 (6580.58-9106.91) | 678 (574-798) | 15212.9 (12857.85-17975.92) | 2.12 (1.97-2.27) |
| Costa Rica | 17928 (14933-21088) | 2618 (2186.65-3065.22) | 68233 (57371-80684) | 5086.2 (4273.63-6019.79) | 2.03 (1.96-2.09) |
| Coted'Ivoire | 39213 (33090-46598) | 1688.9 (1432.33-1995.38) | 219908 (183913-258605) | 3629.8 (3047.04-4252.16) | 2.5 (2.43-2.57) |
| Croatia | 14352 (11835-17152) | 1111.3 (917.31-1327.66) | 20067 (16358-24140) | 1816.7 (1477.18-2186.46) | 1.58 (1.55-1.62) |
| Cuba | 76505 (65017-90031) | 2671.1 (2273.32-3139.61) | 126432 (107528-147779) | 4490.6 (3807.35-5263.25) | 1.54 (1.45-1.62) |
| Cyprus | 3282 (2717-3891) | 1628.1 (1347.4-1929.96) | 13078 (10839-15422) | 3170.6 (2615.61-3755.73) | 2.11 (2.07-2.14) |
| Czechia | 34520 (28770-41159) | 1191.9 (993.04-1420.86) | 59580 (48978-72155) | 1893.3 (1548.18-2294.83) | 1.48 (1.46-1.51) |
| Democratic People's Republic of Korea | 109292 (91836-128889) | 1997.9 (1682.56-2351.22) | 271258 (227207-317044) | 3853.9 (3219.2-4517.64) | 2.06 (2.03-2.08) |
| Democratic Republic of the Congo | 109444 (91298-130181) | 1488.1 (1244.67-1761.93) | 576571 (480977-687641) | 3092.4 (2592.99-3664.42) | 2.22 (2.18-2.27) |
| Denmark | 18003 (15388-20869) | 1303.8 (1112.04-1516.01) | 36331 (30929-42488) | 2657.2 (2255.42-3115.6) | 2.32 (2.24-2.39) |
| Djibouti | 786 (655-934) | 933.9 (781.38-1102.68) | 5645 (4772-6636) | 1767.1 (1493.66-2077.28) | 2.04 (2.01-2.08) |
| Dominica | 603 (507-712) | 4070.7 (3430.08-4790.3) | 1179 (986-1394) | 7146 (5977.65-8451.84) | 1.78 (1.69-1.86) |
| Dominican Republic | 42908 (36194-50939) | 2723.4 (2304.08-3228.08) | 162625 (136359-191929) | 5738.9 (4814.3-6770.77) | 2.47 (2.42-2.53) |
| Ecuador | 34389 (28686-41381) | 1623 (1359.02-1948.81) | 162912 (138781-191111) | 3527.1 (3006.29-4135.77) | 2.71 (2.6-2.83) |
| Egypt | 144256 (121232-169687) | 1250.9 (1054.66-1466.87) | 1024642 (854235-1209860) | 4225 (3527.79-4983.66) | 4.09 (4-4.19) |
| El Salvador | 23968 (20296-28046) | 2154.3 (1833.84-2509.78) | 75465 (63640-89273) | 4304.8 (3631.86-5092.24) | 2 (1.89-2.11) |
| Equatorial Guinea | 1503 (1243-1784) | 1726.1 (1432.43-2038.07) | 12110 (10001-14555) | 3748.5 (3107.54-4480.67) | 2.55 (2.53-2.56) |
| Eritrea | 8108 (6694-9652) | 1177.6 (976-1394.89) | 36365 (30398-43109) | 2398.3 (2009.97-2833.06) | 2.29 (2.26-2.32) |
| Estonia | 4832 (4049-5704) | 1180.8 (989.97-1393.43) | 8205 (6810-9809) | 2413 (1996.01-2893.38) | 2.25 (2.16-2.34) |
| Eswatini | 2495 (2078-2961) | 1556.7 (1303.61-1837.1) | 7987 (6665-9408) | 2834.4 (2369.16-3331.44) | 1.94 (1.75-2.13) |
| Ethiopia | 168212 (141605-197103) | 1708.1 (1444.91-1989.29) | 541000 (450744-640079) | 2238.8 (1875.76-2630.98) | 0.64 (0.55-0.73) |
| Fiji | 9405 (7955-11055) | 5338.2 (4530.13-6260.04) | 24995 (21436-29055) | 10949.9 (9388.47-12729.05) | 2.31 (2.24-2.38) |
| Finland | 34055 (29237-39294) | 2478.3 (2127.3-2863.39) | 62975 (53574-73062) | 5092.9 (4324.01-5921.41) | 2.22 (2.12-2.32) |
| France | 142675 (121062-166493) | 965.1 (818.81-1126.7) | 342297 (288571-403841) | 2220 (1862.77-2629.21) | 3.14 (3-3.28) |
| Gabon | 3290 (2724-3950) | 1783.3 (1483.48-2128.22) | 16407 (13655-19728) | 3689.2 (3083.25-4416.12) | 2.31 (2.24-2.38) |
| Gambia | 2865 (2406-3387) | 1502 (1270.06-1759.73) | 17594 (14805-20864) | 3287.7 (2786.34-3872.43) | 2.58 (2.52-2.64) |
| Georgia | 19637 (16371-23146) | 1455.4 (1214.07-1714.11) | 32136 (27136-37911) | 3503 (2947.76-4145.77) | 2.92 (2.79-3.04) |
| Germany | 242498 (208324-280607) | 1182.6 (1014.58-1369.84) | 583002 (494224-680244) | 3058.7 (2582.68-3580.46) | 3.09 (3-3.18) |
| Ghana | 54455 (45425-64628) | 1766.5 (1481.49-2082.09) | 288094 (239634-339724) | 3382.4 (2819.41-3976.28) | 2.19 (2.09-2.29) |
| Greece | 46487 (38886-55011) | 1790.3 (1495.31-2121.92) | 95348 (80390-112145) | 3830.5 (3202.8-4539.28) | 2.53 (2.51-2.56) |
| Greenland | 71 (58-87) | 508.1 (417.87-618.68) | 214 (181-248) | 1679.1 (1418.03-1950.79) | 3.99 (3.81-4.18) |
| Grenada | 694 (580-822) | 4218.5 (3544.76-4979.91) | 1820 (1522-2191) | 6971.3 (5832.32-8391.59) | 1.56 (1.52-1.6) |
| Guam | 1174 (989-1372) | 3627.1 (3067.68-4221.92) | 2568 (2202-2978) | 6852.8 (5863.49-7960.43) | 1.97 (1.92-2.03) |
| Guatemala | 39470 (33259-46668) | 2550.6 (2162.19-3001.15) | 248856 (209789-296454) | 6275.6 (5309.55-7452.86) | 3.13 (3.02-3.24) |
| Guinea | 18158 (15447-21212) | 1459.2 (1243.3-1700.53) | 87098 (74107-102732) | 2963.6 (2532.12-3475.39) | 2.33 (2.1-2.56) |
| Guinea-Bissau | 4255 (3580-5125) | 2093.5 (1769.42-2510.92) | 19654 (16507-23324) | 4186.6 (3536-4943.51) | 2.2 (2.12-2.28) |
| Guyana | 9510 (7837-11443) | 5727.4 (4741.87-6857.9) | 21992 (18402-26092) | 11250.3 (9417.21-13336.43) | 2.32 (2.22-2.41) |
| Haiti | 63858 (52893-75621) | 4862.6 (4040.09-5743.96) | 278673 (232428-329960) | 8343.4 (6971.94-9861.59) | 1.69 (1.65-1.73) |
| Honduras | 23763 (19496-28438) | 2712.1 (2234.04-3229.35) | 134754 (113001-160181) | 5235.6 (4408.11-6208.53) | 2.1 (2.05-2.15) |
| Hungary | 35607 (30009-42052) | 1237.4 (1041.67-1462.33) | 54612 (44723-65322) | 1895.1 (1547.82-2275.4) | 1.4 (1.29-1.5) |
| Iceland | 1023 (859-1206) | 1593.9 (1340.85-1877.58) | 2930 (2471-3414) | 3452.8 (2905.5-4032.06) | 2.53 (2.5-2.56) |
| India | 4011306 (3346396-4726661) | 2195.9 (1838.1-2578.72) | 15332630 (12864361-18015375) | 4161.7 (3496.36-4884.25) | 1.99 (1.96-2.02) |
| Indonesia | 619691 (521382-729512) | 1483.7 (1253.08-1741.26) | 1934887 (1639458-2277519) | 2430.7 (2056.67-2863.27) | -0.32 (-1.1-0.47) |
| Iran (Islamic Republic of) | 152952 (126344-182046) | 1516 (1261.18-1793.77) | 938777 (782977-1112045) | 3623.1 (3017.57-4300.32) | 2.46 (2.3-2.61) |
| Iraq | 143432 (121259-168723) | 4324.8 (3681.34-5051.13) | 928178 (777944-1107534) | 9442.7 (7935.75-11241.05) | 2.7 (2.55-2.85) |
| Ireland | 14057 (11840-16553) | 1627.3 (1371.84-1915.39) | 38169 (32216-44563) | 2924.8 (2461.23-3428.69) | 2 (1.93-2.07) |
| Israel | 21060 (17345-25328) | 1787.8 (1474.52-2147.38) | 60726 (50277-72733) | 2635 (2175.2-3163.2) | 1.27 (1.22-1.32) |
| Italy | 276696 (226216-332982) | 1876.5 (1533.04-2260.09) | 436110 (356870-522994) | 3062 (2487.7-3701) | 1.49 (1.37-1.6) |
| Jamaica | 11521 (9786-13361) | 2365.1 (2015.22-2732.32) | 34797 (29152-40574) | 4542 (3803.76-5294.02) | 2.08 (2.02-2.15) |
| Japan | 558074 (466086-661016) | 1574.3 (1309.02-1870.19) | 886824 (744606-1043035) | 3002.1 (2503.33-3552.08) | 2.02 (1.98-2.07) |
| Jordan | 18371 (15303-21730) | 2970.4 (2487.81-3491.91) | 171845 (144374-202004) | 5992.8 (5043.25-7033.34) | 2.42 (2.31-2.52) |
| Kazakhstan | 73785 (61609-88279) | 1945 (1629.1-2324.09) | 229457 (197816-263979) | 4480.9 (3854.59-5167.07) | 2.91 (2.79-3.03) |
| Kenya | 40955 (34159-48425) | 951.8 (799.35-1114.9) | 150053 (125267-176437) | 1289.4 (1081.49-1507.92) | 0.82 (0.76-0.89) |
| Kiribati | 1037 (876-1224) | 6383.3 (5414.08-7505.66) | 3568 (3016-4181) | 11866.9 (10054.28-13875.02) | 2.11 (2.01-2.22) |
| Kuwait | 12392 (10312-14707) | 3572.5 (2980.56-4214.16) | 129005 (110174-150228) | 7573.8 (6445.7-8845.5) | 2.42 (2.33-2.51) |
| Kyrgyzstan | 11251 (9295-13252) | 1269.9 (1051.24-1486.11) | 46073 (38643-54366) | 2735.7 (2298.58-3223.39) | 2.56 (2.45-2.66) |
| Lao People's Democratic Republic | 13648 (11558-16115) | 1649.7 (1403.52-1942.96) | 62951 (53058-74219) | 3409.5 (2881.4-4014.91) | 2.2 (2.13-2.26) |
| Latvia | 7631 (6313-9094) | 1084.5 (896.31-1293.44) | 11325 (9432-13525) | 2326.9 (1932.61-2787.17) | 2.38 (2.28-2.48) |
| Lebanon | 17795 (14832-21279) | 2592.4 (2164.63-3093.63) | 83319 (70607-97188) | 5270.1 (4460.35-6154.33) | 2.4 (2.35-2.45) |
| Lesotho | 3567 (2975-4241) | 1034.4 (865.96-1227.35) | 10579 (8923-12462) | 2380.6 (2013.96-2790.38) | 2.79 (2.76-2.82) |
| Liberia | 7887 (6637-9315) | 1648.7 (1399.42-1936.87) | 45250 (38480-53581) | 3538.8 (3021.55-4173.31) | 2.55 (2.47-2.63) |
| Libya | 14080 (11696-16797) | 2049.9 (1713.14-2427.92) | 114471 (96322-135238) | 5355.4 (4495.8-6338.82) | 3.4 (3.21-3.6) |
| Lithuania | 9221 (7615-10986) | 962.2 (794.96-1146.14) | 13880 (11592-16404) | 2024.9 (1684.82-2402.97) | 2.32 (2.24-2.4) |
| Luxembourg | 1695 (1428-1991) | 1649.7 (1386.83-1940.72) | 5885 (4930-6904) | 3351.5 (2795.1-3949.81) | 2.31 (2.28-2.33) |
| Madagascar | 25523 (21512-30032) | 1097.6 (931.55-1284.97) | 118374 (99702-140315) | 1850.1 (1566.9-2181.23) | 1.59 (1.54-1.64) |
| Malawi | 16724 (14123-19959) | 866.2 (735.92-1026.91) | 54592 (46345-63725) | 1271.9 (1085.01-1475.93) | 1.06 (0.96-1.16) |
| Malaysia | 81718 (67663-98203) | 2096.7 (1741.12-2515.89) | 284272 (238938-334085) | 3442.3 (2895.44-4041.91) | 1.62 (1.41-1.82) |
| Maldives | 584 (491-691) | 1577.8 (1336.49-1859.77) | 3099 (2591-3651) | 2559.9 (2139.42-3019.85) | 1.43 (1.29-1.57) |
| Mali | 48446 (40267-57978) | 2817.2 (2355.79-3353.08) | 266692 (220795-319537) | 5656.5 (4719.78-6728.94) | 2.5 (2.23-2.77) |
| Malta | 1676 (1416-1961) | 1653.8 (1394.01-1939.65) | 4627 (3926-5411) | 4299.4 (3627.42-5049.93) | 3.22 (3.15-3.29) |
| Marshall Islands | 617 (512-742) | 7822.5 (6560.06-9311.09) | 2808 (2370-3333) | 19554.5 (16529.4-23190.47) | 3.23 (3.06-3.4) |
| Mauritania | 5586 (4697-6608) | 1369.1 (1156.88-1610.87) | 22575 (19503-26177) | 2412.9 (2094.62-2776.8) | 1.58 (1.49-1.67) |
| Mauritius | 6450 (5414-7586) | 2412 (2029.46-2833.18) | 18502 (15399-21832) | 5332.6 (4433.13-6305.43) | 2.76 (2.64-2.87) |
| Mexico | 914823 (765729-1069782) | 5043.2 (4241.25-5870.92) | 2536939 (2154315-2945292) | 7019 (5953.49-8154.39) | 1.05 (0.85-1.26) |
| Micronesia (Federated States of) | 967 (821-1135) | 5038.5 (4306.11-5878.4) | 2682 (2302-3114) | 10962.3 (9425.44-12714.03) | 2.81 (2.53-3.09) |
| Monaco | 120 (102-140) | 1520.6 (1282.78-1778.26) | 268 (227-314) | 3294.9 (2773.6-3886.89) | 2.54 (2.51-2.57) |
| Mongolia | 4994 (4186-5868) | 1194.3 (1008.12-1390.95) | 24224 (20628-28329) | 2681.9 (2280-3141.56) | 2.72 (2.65-2.79) |
| Montenegro | 2178 (1794-2594) | 1427 (1175.76-1699.74) | 3910 (3267-4646) | 2289.1 (1907.14-2729.28) | 1.54 (1.48-1.6) |
| Morocco | 145066 (119958-173383) | 2742.5 (2281.97-3268) | 857378 (712107-1020874) | 8634.3 (7166.94-10287.31) | 3.91 (3.81-4.01) |
| Mozambique | 24418 (20413-29107) | 861.4 (722.78-1023.95) | 118449 (98297-143045) | 1829.9 (1524.99-2198.43) | 2.49 (2.45-2.53) |
| Myanmar | 212825 (179731-251887) | 2425.1 (2056.7-2865.23) | 631162 (544589-724574) | 4169.9 (3597.6-4787.72) | 1.35 (1.22-1.49) |
| Namibia | 3789 (3136-4519) | 1345.5 (1122.28-1594.39) | 13539 (11405-15976) | 2204.8 (1862.73-2594.8) | 1.52 (1.46-1.58) |
| Nauru | 128 (108-152) | 5950.5 (5013.56-7013.59) | 325 (277-380) | 12444.5 (10649.87-14530.22) | 2.18 (2.05-2.31) |
| Nepal | 101000 (84251-120017) | 2497 (2094.41-2952.87) | 464990 (396671-542643) | 5447.4 (4658.2-6342.33) | 2.49 (2.18-2.79) |
| Netherlands | 48416 (40798-56716) | 1183.7 (997.3-1387.19) | 99128 (82690-116245) | 2498.1 (2076.76-2937.94) | 2.43 (2.38-2.48) |
| New Zealand | 13386 (11074-16132) | 1491.2 (1234.85-1796.78) | 33675 (30347-37295) | 2626.7 (2362.99-2913.86) | 2.31 (1.85-2.76) |
| Nicaragua | 20777 (17373-24500) | 2865.3 (2409.62-3357.27) | 87533 (74043-104053) | 5011.2 (4244.7-5952) | 1.72 (1.67-1.77) |
| Niger | 25043 (21135-29614) | 1663 (1412.11-1952.64) | 146369 (122507-173271) | 3291.5 (2772.29-3862.17) | 2.19 (2.15-2.23) |
| Nigeria | 230128 (192309-272949) | 1374 (1156.65-1617.67) | 1050705 (877886-1242994) | 2122.5 (1784.54-2494.56) | 1.42 (1.35-1.48) |
| Niue | 29 (24-34) | 5930.8 (5018.4-6976.55) | 57 (49-67) | 14106.7 (12027.45-16675.19) | 2.9 (2.8-3) |
| North Macedonia | 6684 (5426-8197) | 1319.4 (1071.6-1617.03) | 14326 (11614-17401) | 2223.3 (1797.17-2707.52) | 1.68 (1.62-1.74) |
| Northern Mariana Islands | 475 (403-559) | 3842 (3281.21-4503.89) | 956 (805-1117) | 7421.1 (6212.17-8736.58) | 2.01 (1.95-2.07) |
| Norway | 24020 (20233-28092) | 2197 (1849.05-2572.93) | 41065 (34501-47954) | 3117.9 (2610.43-3652.01) | 1.02 (0.96-1.08) |
| Oman | 7304 (6172-8599) | 2570.5 (2176.16-3027.2) | 48170 (40556-57312) | 4697.4 (3955.05-5591.48) | 1.27 (0.95-1.59) |
| Pakistan | 465554 (386885-551092) | 2289.6 (1910.46-2699.29) | 2724084 (2291036-3221965) | 4915.2 (4143.62-5797.12) | 2.68 (2.5-2.85) |
| Palau | 234 (195-277) | 6224.2 (5211.98-7353.78) | 589 (511-674) | 13269.5 (11449.17-15315.26) | 2.51 (2.46-2.56) |
| Palestine | 6912 (5759-8128) | 2004.4 (1680.62-2342.48) | 46555 (39081-54871) | 4136.6 (3483.92-4862.18) | 2.36 (2.28-2.43) |
| Panama | 13031 (10990-15443) | 2407.5 (2038.74-2843.29) | 48180 (40520-56519) | 4484.6 (3771.1-5260.36) | 1.96 (1.92-2.01) |
| Papua New Guinea | 30979 (26060-36327) | 3723.7 (3146.35-4340.53) | 213715 (181087-250316) | 8689.1 (7378.7-10158.29) | 2.67 (2.62-2.71) |
| Paraguay | 14784 (12301-17660) | 1848.4 (1540.59-2205.26) | 54997 (46269-64705) | 3073.5 (2589.31-3610.89) | 1.71 (1.62-1.79) |
| Peru | 51013 (42493-60316) | 1094.5 (915.18-1286.09) | 173375 (147205-202696) | 1780 (1510.96-2081.78) | 1.66 (1.46-1.86) |
| Philippines | 240512 (204839-277360) | 1745.9 (1489.65-2010.24) | 605661 (513704-705725) | 2180.1 (1851.72-2537.23) | 0.55 (0.3-0.8) |
| Poland | 134678 (110807-160903) | 1373.5 (1132.99-1641.04) | 200816 (165937-240532) | 1783.1 (1469.35-2142.78) | 0.77 (0.7-0.85) |
| Portugal | 56395 (47108-67320) | 2205.8 (1841.19-2634.51) | 132694 (110436-157547) | 4912.9 (4057.29-5879.09) | 2.65 (2.6-2.71) |
| Puerto Rico | 32569 (27629-38976) | 3410.6 (2893.59-4080.88) | 54164 (45480-64515) | 6444.4 (5399.07-7697.43) | 2.24 (2.16-2.31) |
| Qatar | 2005 (1680-2410) | 2875.5 (2411.76-3466.3) | 39236 (32068-48138) | 6669.6 (5428.04-8250.55) | 2.67 (2.63-2.72) |
| Republic of Korea | 241579 (205021-283170) | 2079.5 (1770.21-2429.74) | 858561 (752432-972753) | 6301.8 (5489.4-7185.03) | 4.09 (3.7-4.48) |
| Republic of Moldova | 16247 (13410-19518) | 1437.3 (1188.79-1724.69) | 31943 (26375-37883) | 2903.7 (2388.24-3455.6) | 2.22 (2.06-2.38) |
| Romania | 56262 (46332-68298) | 985.6 (812.26-1196.54) | 79469 (64881-96909) | 1497.3 (1216.9-1831.08) | 1.27 (1.22-1.32) |
| Russian Federation | 417759 (347166-493690) | 1104.4 (919.07-1305.46) | 849131 (706461-1004143) | 2008.3 (1665.44-2382.73) | 1.78 (1.72-1.84) |
| Rwanda | 14141 (11894-16797) | 1016 (859.33-1199.82) | 40726 (34255-47687) | 1266.3 (1066.17-1479.31) | 0.32 (0.16-0.48) |
| Saint Kitts and Nevis | 317 (266-379) | 3924.9 (3297.58-4675.74) | 969 (805-1157) | 5690.9 (4721.93-6798.52) | 1.01 (0.93-1.09) |
| Saint Lucia | 1398 (1157-1705) | 5037.7 (4174.01-6144.23) | 4068 (3511-4745) | 7984.3 (6868.61-9333.68) | 1.56 (1.5-1.62) |
| Saint Vincent and the Grenadines | 973 (810-1166) | 4599.5 (3842.55-5492.07) | 2323 (1941-2757) | 7874.2 (6578.85-9355.96) | 1.69 (1.62-1.76) |
| Samoa | 1637 (1380-1939) | 5413.3 (4594.85-6378.3) | 5717 (4838-6764) | 12907.8 (10963.14-15224.93) | 2.94 (2.75-3.13) |
| San Marino | 100 (84-117) | 1595.7 (1349.61-1876.75) | 275 (231-323) | 3363.4 (2814.19-3973.48) | 2.45 (2.42-2.48) |
| Sao Tome and Principe | 415 (348-493) | 1898.9 (1607.18-2233.78) | 2330 (1983-2719) | 4453.4 (3805.84-5174.52) | 2.83 (2.81-2.84) |
| Saudi Arabia | 62012 (52368-72541) | 2413.5 (2048.25-2810.27) | 642765 (542354-755850) | 5927.9 (4998.3-6976.65) | 2.99 (2.84-3.13) |
| Senegal | 33195 (27640-39323) | 2241.6 (1876.51-2636.79) | 141345 (120946-165480) | 4033.6 (3467.01-4700.63) | 2.19 (2.07-2.3) |
| Serbia | 36648 (30414-44170) | 1486.4 (1232.74-1792.77) | 52885 (43578-63920) | 2152.5 (1768.37-2609.03) | 1.08 (1.04-1.12) |
| Seychelles | 246 (205-293) | 1625.2 (1365.9-1933.86) | 1365 (1130-1639) | 5073.9 (4184.51-6109.2) | 3.72 (3.63-3.8) |
| Sierra Leone | 13683 (11545-16158) | 1561.1 (1324.91-1834.63) | 71864 (60261-85997) | 3603.1 (3036.95-4286.81) | 2.78 (2.69-2.86) |
| Singapore | 24051 (20019-28665) | 2667.3 (2224.09-3173.92) | 82895 (70788-96980) | 4751.9 (4031.86-5588.82) | 1.68 (1.51-1.86) |
| Slovakia | 14117 (11498-17060) | 1029.2 (839.32-1243.81) | 26212 (21337-31408) | 1605.4 (1303.75-1928.87) | 1.4 (1.36-1.45) |
| Slovenia | 5488 (4516-6545) | 1053.1 (866.99-1255.82) | 9094 (7375-10880) | 1655 (1339.88-1986.25) | 1.46 (1.39-1.53) |
| Solomon Islands | 2011 (1708-2362) | 3388.5 (2897.23-3965.73) | 11596 (10023-13378) | 7271.4 (6297.05-8373.02) | 2.55 (2.49-2.61) |
| Somalia | 17358 (14489-20684) | 1129.7 (945.62-1341.82) | 84819 (70607-101525) | 2020.4 (1687-2404.35) | 1.83 (1.8-1.85) |
| South Africa | 175998 (147120-208731) | 2083.9 (1748.83-2459.4) | 471162 (394891-555505) | 3013.5 (2524.17-3555.17) | 1.06 (0.97-1.14) |
| South Sudan | 10223 (8505-12245) | 936.1 (784.06-1113.63) | 36133 (30369-42965) | 1726 (1456.7-2044.04) | 1.94 (1.91-1.98) |
| Spain | 169103 (140998-200741) | 1762.4 (1469.5-2091.64) | 514701 (434772-598255) | 4393.9 (3667.94-5152.72) | 2.95 (2.72-3.18) |
| Sri Lanka | 84140 (69639-100590) | 1992 (1653.66-2376.56) | 288521 (241648-341671) | 4799.1 (4014.78-5689.48) | 2.88 (2.66-3.11) |
| Sudan | 72998 (61194-86651) | 1899.4 (1600.65-2245.17) | 463070 (392281-535702) | 4702.2 (4004.81-5415.99) | 2.87 (2.79-2.95) |
| Suriname | 2988 (2498-3580) | 3480 (2915.5-4160) | 11307 (9627-13190) | 7478.4 (6360.31-8731.88) | 2.64 (2.59-2.7) |
| Sweden | 44060 (36819-52155) | 1984.2 (1652.3-2357.93) | 74829 (61961-89120) | 3100.7 (2558.92-3708.23) | 1.33 (1.23-1.42) |
| Switzerland | 40712 (34025-47663) | 2158.1 (1800.13-2532.01) | 99501 (83693-116415) | 4476.1 (3742.44-5259.31) | 2.46 (2.33-2.59) |
| Syrian Arab Republic | 53731 (45130-63438) | 2470.8 (2091.14-2894.37) | 180630 (151279-212053) | 4628.2 (3871.71-5433.4) | 1.9 (1.86-1.95) |
| Taiwan (Province of China) | 105309 (88976-123071) | 2055.5 (1740.9-2391.28) | 191622 (162512-223455) | 2844.4 (2393.06-3334.05) | 0.84 (0.7-0.98) |
| Tajikistan | 12063 (10180-14197) | 1282.4 (1094.32-1494.45) | 63857 (54023-74750) | 2713 (2299.91-3171.81) | 2.4 (2.33-2.47) |
| Thailand | 195145 (164404-230049) | 1354.5 (1142.49-1593.62) | 485760 (408985-572788) | 2499.9 (2094.34-2953.91) | 1.96 (1.88-2.04) |
| Timor-Leste | 1719 (1455-2019) | 1057.2 (897.6-1237.79) | 7843 (6497-9277) | 2751.8 (2285.55-3244.07) | 3.12 (3.08-3.17) |
| Togo | 9047 (7609-10688) | 1227.2 (1036.66-1443.56) | 45358 (38833-52975) | 2233.6 (1915.47-2601.88) | 1.89 (1.79-1.99) |
| Tokelau | 21 (18-25) | 6580 (5561.46-7790.47) | 41 (35-48) | 12540.4 (10621.57-14789.47) | 2.06 (1.96-2.16) |
| Tonga | 1052 (885-1242) | 5539.1 (4679.73-6509.84) | 2600 (2202-3061) | 10983.7 (9320.72-12907.68) | 2.29 (2.25-2.34) |
| Trinidad and Tobago | 12290 (10319-14344) | 4497.3 (3784.34-5223.89) | 28376 (23764-33506) | 7455.8 (6233.53-8820.08) | 1.49 (1.4-1.57) |
| Tunisia | 32600 (27050-38814) | 1904 (1589.07-2256.03) | 167827 (140375-197907) | 4924.4 (4108.34-5821.44) | 3.03 (2.94-3.11) |
| Turkey | 184920 (159243-211675) | 1472.4 (1270.87-1679.63) | 840166 (714801-974698) | 3579.1 (3040.31-4158.8) | 2.76 (2.45-3.07) |
| Turkmenistan | 7908 (6734-9274) | 1090.7 (935.16-1269.2) | 34028 (29215-39234) | 2737.5 (2351.16-3155.67) | 3.06 (2.94-3.17) |
| Tuvalu | 100 (85-118) | 4208.8 (3580.59-4961.02) | 243 (208-284) | 8987.3 (7722.7-10469.05) | 2.34 (2.29-2.39) |
| Uganda | 26010 (21710-30763) | 815.2 (685.76-954.87) | 126239 (106483-149506) | 1430.7 (1213.94-1683.65) | 1.71 (1.67-1.76) |
| Ukraine | 174225 (145470-206026) | 1306.9 (1089.22-1547.56) | 286922 (235357-342359) | 2268.4 (1854.39-2713.31) | 1.62 (1.48-1.76) |
| United Arab Emirates | 5971 (4939-7145) | 2141.5 (1774.43-2564.41) | 94649 (78718-112256) | 4328.4 (3589.98-5150.6) | 2.2 (2.11-2.29) |
| United Kingdom | 292528 (243702-344656) | 1977.1 (1645.02-2332.32) | 1019867 (873115-1179326) | 6185.9 (5268.57-7180.45) | 3.6 (3.39-3.81) |
| United Republic of Tanzania | 40956 (34603-48258) | 804.5 (684.78-943.08) | 212429 (179195-250870) | 1617.9 (1369.19-1901.25) | 2.24 (2.14-2.33) |
| United States of America | 1153026 (983755-1337187) | 1669.1 (1424.5-1936.34) | 3447060 (3065323-3873515) | 4213.4 (3741.69-4740.39) | 3.09 (3.02-3.16) |
| United States Virgin Islands | 1322 (1093-1580) | 4299.5 (3552.78-5140.87) | 1538 (1292-1813) | 7680.2 (6427.77-9096.66) | 2.05 (1.93-2.18) |
| Uruguay | 7087 (5855-8407) | 938.3 (775.12-1113.18) | 20854 (17430-24865) | 2299.2 (1918.65-2745.18) | 3.16 (3.04-3.28) |
| Uzbekistan | 55915 (47388-65260) | 1401.2 (1197.85-1623.01) | 322514 (277497-378556) | 3522.1 (3029.71-4133.85) | 3.08 (2.96-3.21) |
| Vanuatu | 1038 (873-1219) | 3475.8 (2944.31-4067.7) | 6282 (5391-7285) | 8706.8 (7500.35-10063.14) | 3 (2.97-3.02) |
| Venezuela (Bolivarian Republic of) | 104841 (88127-123019) | 2494.8 (2104.28-2911.2) | 337085 (284645-392785) | 4558 (3843.52-5320.67) | 2.11 (1.99-2.22) |
| Viet Nam | 168736 (141231-197525) | 1212.1 (1019.25-1412.56) | 546964 (465159-629524) | 1968.9 (1670.91-2270.5) | 1.28 (1.08-1.47) |
| Yemen | 35300 (29363-42014) | 1585 (1326.96-1881.6) | 241642 (201824-286979) | 3339.4 (2799.52-3956.72) | 1.48 (0.93-2.02) |
| Zambia | 24614 (20547-29517) | 1612.6 (1353.15-1914.57) | 131563 (110590-155290) | 3044.8 (2573.91-3565.59) | 1.92 (1.87-1.97) |
| Zimbabwe | 24124 (20039-28873) | 1221.3 (1021.99-1450.3) | 84801 (70455-100809) | 2341.4 (1950.71-2776.82) | 2.14 (2.09-2.18) |

Note: ASR is per 100,000 women of childbearing age. EAPC, estimated annual percentage change; ASR, age-standardized rate; UI, uncertainty interval; CI, confidence interval.

**Supplementary Table 4.** Disability-adjusted life years of diabetes in women of childbearing age in 1990 and 2021 for 204 countries or territories, with EAPC from 1990 to 2021.

| Location | Case no. in 1990 (95% UI) | ASR in 1990 (95% UI) | Case no. in 2021 (95% UI) | ASR in 2021 (95% UI) | EAPC (95% CI) (%) |
| --- | --- | --- | --- | --- | --- |
| Afghanistan | 10468 (7418-14726) | 531 (377.37-746.5) | 68235 (48386-92727) | 1129.1 (801.34-1533.14) | 2.59 (2.53-2.66) |
| Albania | 712 (504-979) | 102.4 (71.69-142.31) | 917 (610-1324) | 136.6 (91.06-196.92) | 0.98 (0.91-1.06) |
| Algeria | 10373 (7464-14010) | 230.8 (166.28-311.66) | 58036 (40429-79310) | 489.9 (340.77-669.95) | 2.51 (2.47-2.55) |
| American Samoa | 84 (62-111) | 891.3 (664.31-1165.99) | 247 (182-329) | 1998.6 (1468.52-2668.24) | 2.51 (2.21-2.8) |
| Andorra | 19 (14-27) | 126.8 (89.65-174.91) | 56 (37-79) | 224.5 (148.52-319.82) | 1.9 (1.84-1.95) |
| Angola | 6851 (4906-9248) | 363.1 (260.66-489.4) | 30483 (21853-41338) | 468.7 (337.34-634.8) | 0.84 (0.72-0.96) |
| Antigua and Barbuda | 99 (82-122) | 688.4 (565.4-846.22) | 187 (138-252) | 688.5 (508.93-929.61) | -0.02 (-0.13-0.1) |
| Argentina | 14745 (12186-17834) | 186.3 (153.95-225.33) | 29580 (21973-39592) | 235.5 (174.96-315.03) | 0.84 (0.72-0.96) |
| Armenia | 2071 (1711-2545) | 267.6 (219.63-332.04) | 2495 (1863-3339) | 304.5 (228.73-405.25) | 0.4 (0.14-0.66) |
| Australia | 4866 (3707-6347) | 106.9 (81.47-139.54) | 10097 (7068-13989) | 148.9 (104.2-206.23) | 1.02 (0.93-1.1) |
| Austria | 2088 (1586-2752) | 100.9 (76.59-133.13) | 3578 (2373-5164) | 158.2 (104.67-228.47) | 1.45 (1.38-1.51) |
| Azerbaijan | 3530 (2762-4502) | 226.4 (177.16-289.5) | 10576 (7637-14588) | 365.5 (264.73-503.13) | 1.12 (0.9-1.35) |
| Bahamas | 430 (347-527) | 689.7 (556.97-845.9) | 909 (665-1220) | 791.4 (579.69-1062.25) | 0.2 (0.06-0.34) |
| Bahrain | 387 (293-502) | 435 (330.84-561.57) | 2227 (1637-2974) | 654.7 (481.36-873.69) | 1.34 (1.16-1.51) |
| Bangladesh | 91002 (67297-118149) | 445.1 (330.99-575.71) | 278286 (205629-371695) | 627.2 (463.77-837.58) | 1.45 (1.28-1.63) |
| Barbados | 448 (374-539) | 708.8 (590.77-852.2) | 624 (472-830) | 759.6 (574.51-1008.34) | -0.01 (-0.13-0.12) |
| Belarus | 3134 (2445-4052) | 123 (95.93-159.34) | 4770 (3533-6448) | 186.6 (139.31-250.29) | 0.47 (0.08-0.85) |
| Belgium | 3841 (2741-5215) | 151.7 (108.2-206.32) | 7007 (4599-10232) | 251.4 (164.67-366.81) | 1.64 (1.52-1.76) |
| Belize | 210 (174-254) | 662.2 (550.68-802.21) | 924 (721-1195) | 833.3 (650.65-1077.86) | 0.74 (0.49-0.98) |
| Benin | 2484 (1825-3300) | 274.3 (202.09-363.68) | 13640 (9647-18732) | 493.3 (350.07-677.69) | 1.74 (1.65-1.83) |
| Bermuda | 54 (42-69) | 303.5 (237.93-388.23) | 61 (42-86) | 369.9 (254.58-517.79) | 0.43 (0.26-0.59) |
| Bhutan | 338 (236-465) | 306.9 (217.18-420.57) | 728 (520-996) | 373.9 (267.28-511.21) | 0.4 (0.31-0.5) |
| Bolivia (Plurinational State of) | 4651 (3415-6268) | 362.8 (266.12-488.97) | 11489 (8281-15561) | 389.3 (280.43-527.11) | 0 (-0.1-0.11) |
| Bosnia and Herzegovina | 2017 (1543-2612) | 179.2 (136.97-232.33) | 2100 (1449-2958) | 233.3 (162.03-326.47) | 0.77 (0.65-0.89) |
| Botswana | 630 (433-909) | 262.4 (179.25-382.71) | 2158 (1557-2948) | 332.4 (239.81-454.23) | 1.34 (0.9-1.79) |
| Brazil | 127225 (109426-148625) | 377.4 (323.86-441.32) | 235409 (188380-293704) | 369.2 (295.91-460.43) | -0.24 (-0.41--0.08) |
| Brunei Darussalam | 353 (266-461) | 616.1 (463.1-808.29) | 901 (655-1212) | 681.9 (495.44-917.46) | 0.66 (0.32-1.01) |
| Bulgaria | 4880 (3933-6171) | 215.9 (174.79-271.8) | 4638 (3415-6291) | 248 (184.02-333.78) | 0.35 (0.24-0.46) |
| Burkina Faso | 5300 (3746-7349) | 300.6 (212.63-417.03) | 17062 (12306-23128) | 371.5 (269.81-502.24) | 0.66 (0.55-0.77) |
| Burundi | 4260 (2852-6145) | 419.8 (278.74-609.72) | 9782 (6874-13535) | 383.2 (267.86-533.69) | -0.79 (-0.97--0.61) |
| Cabo Verde | 139 (101-187) | 213.6 (155.62-288.51) | 575 (409-782) | 403.7 (287.47-550.03) | 1.84 (1.4-2.28) |
| Cambodia | 6914 (4917-9731) | 327.8 (232.84-462.78) | 15676 (11173-21362) | 369.8 (263.4-504.01) | 0.17 (0.02-0.32) |
| Cameroon | 6380 (4507-8791) | 324 (229.1-447.09) | 31433 (22054-43328) | 470.2 (330.1-645.57) | 1.09 (0.86-1.32) |
| Canada | 9814 (7972-12093) | 129.1 (104.62-159.45) | 20882 (14832-28278) | 223 (158.75-301.73) | 1.35 (1.18-1.53) |
| Central African Republic | 2585 (1824-3559) | 498.1 (349.39-689.67) | 8302 (5985-11298) | 707.9 (510.97-963.95) | 1.12 (1.01-1.22) |
| Chad | 2798 (2022-3823) | 250.8 (180.95-343.46) | 14026 (10005-19099) | 443.2 (316.12-604.17) | 1.86 (1.63-2.1) |
| Chile | 4687 (3691-5947) | 143.4 (112.72-182.18) | 9802 (6825-13667) | 191.1 (133.2-266.3) | 1.04 (0.93-1.16) |
| China | 585261 (432698-779814) | 205.8 (152.79-272.9) | 1158382 (783613-1623879) | 318 (213.33-448.64) | 1.33 (1.09-1.57) |
| Colombia | 25005 (19389-32513) | 343 (265.7-446.61) | 50741 (36076-69975) | 381.8 (271.5-526.59) | -0.13 (-0.4-0.14) |
| Comoros | 342 (209-504) | 398.4 (249.74-582.86) | 845 (605-1146) | 461.4 (330.39-625.22) | 0.23 (0.01-0.45) |
| Congo | 2049 (1437-2874) | 482.5 (337.29-679.84) | 8274 (5872-11443) | 624.5 (442.87-866.14) | 0.62 (0.38-0.85) |
| Cook Islands | 56 (41-73) | 1426.5 (1050.69-1870.16) | 74 (54-99) | 1607.4 (1175.35-2169.33) | 0.43 (0.37-0.49) |
| Costa Rica | 1922 (1419-2586) | 297.6 (220.95-398.09) | 6339 (4467-8871) | 472.1 (332.67-661.12) | 1.31 (1.18-1.44) |
| Coted'Ivoire | 5630 (4059-7682) | 255.5 (184.83-348.66) | 26031 (18643-35238) | 445.3 (320.12-601.55) | 2.13 (1.92-2.33) |
| Croatia | 1875 (1428-2430) | 146.2 (111.65-189.18) | 1930 (1313-2762) | 173.9 (118.94-247.45) | 0.64 (0.55-0.73) |
| Cuba | 10757 (8365-13937) | 379 (294.64-491.61) | 12306 (8371-17470) | 423.6 (287.44-601.35) | 0.05 (-0.15-0.26) |
| Cyprus | 404 (301-530) | 200.4 (149.44-263.1) | 963 (660-1345) | 228.5 (155.68-320.29) | -0.01 (-0.17-0.16) |
| Czechia | 4364 (3315-5695) | 152.2 (116.35-197.78) | 5782 (4000-8313) | 183.1 (128.14-260.44) | 0.77 (0.63-0.9) |
| Democratic People's Republic of Korea | 12582 (8938-16944) | 230.9 (164.37-310.61) | 25197 (17948-34710) | 351.4 (249.95-484.51) | 1.3 (1.28-1.32) |
| Democratic Republic of the Congo | 24209 (16688-34222) | 349.5 (241.04-496.7) | 82551 (58565-114031) | 464 (330.32-642.26) | 0.85 (0.77-0.94) |
| Denmark | 1639 (1256-2110) | 116.1 (88.54-150.13) | 2287 (1508-3229) | 164.6 (108.17-233.17) | 0.92 (0.78-1.06) |
| Djibouti | 201 (134-289) | 262.3 (175.87-378.86) | 1063 (730-1504) | 340.4 (233.59-482.28) | 0.8 (0.63-0.98) |
| Dominica | 98 (76-126) | 680.3 (524.54-867.1) | 155 (114-209) | 936.4 (692.46-1263.01) | 0.93 (0.86-0.99) |
| Dominican Republic | 6457 (4948-8470) | 417.4 (320.05-548.39) | 19704 (14293-26392) | 698.2 (506.5-935.33) | 1.9 (1.8-1.99) |
| Ecuador | 5417 (4388-6697) | 266.6 (216.1-329.24) | 17847 (13289-23202) | 387.9 (288.87-504.07) | 1.04 (0.79-1.3) |
| Egypt | 30072 (22692-38678) | 264 (199.04-339.15) | 117486 (87775-156761) | 487.2 (363.79-651.3) | 2.3 (2.13-2.47) |
| El Salvador | 3782 (2955-4794) | 355.8 (278.96-450.06) | 10813 (8104-14113) | 617.7 (462.98-806.36) | 1.61 (1.48-1.75) |
| Equatorial Guinea | 339 (236-500) | 409 (284.49-606.52) | 1601 (1089-2244) | 520.8 (354.81-729.28) | 0.71 (0.61-0.81) |
| Eritrea | 2710 (1900-3747) | 428.6 (300.32-591.34) | 7409 (5196-10123) | 511.9 (358.8-700.63) | 0.8 (0.69-0.9) |
| Estonia | 568 (427-749) | 141 (106.67-185.2) | 850 (618-1165) | 260 (191.3-352.13) | 1.53 (1.2-1.86) |
| Eswatini | 489 (349-663) | 331.6 (235.62-451.26) | 1432 (922-2105) | 555 (355.48-822.4) | 2.11 (1.29-2.93) |
| Ethiopia | 60036 (46611-77580) | 657.2 (512.29-849.29) | 83912 (64755-105341) | 364.5 (282.15-455.24) | -2.55 (-2.8--2.3) |
| Fiji | 2636 (1984-3476) | 1595.6 (1204.16-2101.46) | 5424 (4077-7167) | 2380.7 (1789.97-3144.58) | 1.28 (1.1-1.46) |
| Finland | 2607 (1823-3566) | 186.5 (129.99-255.84) | 4102 (2679-5861) | 327.1 (213.13-468.3) | 1.71 (1.59-1.84) |
| France | 12023 (8997-15914) | 81.5 (61.04-107.92) | 21856 (14308-31404) | 139.5 (91-200.91) | 2.02 (1.91-2.12) |
| Gabon | 667 (475-908) | 393.6 (279.24-539.27) | 2226 (1562-3054) | 520.6 (367.05-713.79) | 0.76 (0.51-1.01) |
| Gambia | 439 (303-613) | 245.9 (169.84-343.31) | 2288 (1588-3204) | 446.5 (312.84-625.56) | 1.8 (1.63-1.96) |
| Georgia | 2431 (1902-3171) | 181.9 (142.31-237.4) | 3101 (2237-4290) | 337.5 (244.69-464.59) | 1.94 (1.7-2.19) |
| Germany | 23830 (18371-30622) | 115.6 (89.05-148.7) | 38202 (25697-53626) | 197.5 (132.45-278.05) | 1.84 (1.64-2.04) |
| Ghana | 10398 (7610-13928) | 354.9 (260.08-475.27) | 35440 (25675-47711) | 432.9 (314.06-582.71) | 0.63 (0.5-0.75) |
| Greece | 3699 (2513-5239) | 141.5 (96.09-200.3) | 6956 (4523-9961) | 268.1 (173.69-384.78) | 2 (1.95-2.05) |
| Greenland | 15 (11-20) | 111.9 (82.25-148.71) | 18 (13-24) | 139.7 (99-190.29) | 0.29 (0.05-0.54) |
| Grenada | 170 (142-204) | 1041.9 (871.34-1251.4) | 261 (199-344) | 991.9 (759.39-1307.48) | -0.28 (-0.48--0.08) |
| Guam | 132 (98-172) | 437.1 (327.52-565.93) | 244 (179-329) | 633.4 (461.77-856.45) | 1.14 (0.95-1.32) |
| Guatemala | 6246 (5036-7687) | 407.9 (327.71-504.3) | 42560 (34274-52689) | 1108.4 (893.81-1371.24) | 2.95 (2.74-3.16) |
| Guinea | 3603 (2562-4997) | 300.9 (213.78-417.08) | 12846 (9236-17444) | 453.6 (326.53-613.94) | 1.35 (1.3-1.4) |
| Guinea-Bissau | 902 (650-1223) | 463.1 (332.72-628.87) | 2925 (2070-3970) | 656.7 (463.57-895.16) | 1.09 (0.91-1.26) |
| Guyana | 1872 (1495-2330) | 1154.9 (925.33-1438.22) | 3019 (2235-4002) | 1545.4 (1143.87-2050.15) | 0.81 (0.53-1.08) |
| Haiti | 17961 (13001-24415) | 1366.7 (994.83-1845.13) | 48157 (34638-65685) | 1451.9 (1045.17-1978.47) | 0.31 (0.24-0.38) |
| Honduras | 3112 (2311-4178) | 371.6 (276.57-497.9) | 14330 (10070-19908) | 575.6 (405.01-798.88) | 1.26 (1.15-1.37) |
| Hungary | 5083 (4016-6490) | 179.1 (142.19-227.56) | 5515 (3841-7668) | 191.8 (135.48-264.29) | 0.28 (0.18-0.38) |
| Iceland | 71 (48-101) | 112.5 (76.2-158.96) | 188 (121-269) | 218.8 (140.76-313.53) | 2.19 (2.14-2.24) |
| India | 511568 (415878-628438) | 285.4 (232.45-349.15) | 1471387 (1120117-1899221) | 403.4 (307.55-519.85) | 0.97 (0.85-1.08) |
| Indonesia | 126254 (102473-156856) | 306.2 (249.53-379.62) | 284345 (226869-358573) | 355.3 (283.36-448.55) | -0.34 (-0.59--0.09) |
| Iran (Islamic Republic of) | 16751 (12979-21685) | 170.2 (132.07-219.9) | 83706 (61096-112335) | 323.8 (236.85-434.34) | 1.94 (1.81-2.06) |
| Iraq | 18620 (13980-24187) | 581.4 (435.98-753.12) | 83729 (60137-115202) | 859.4 (618.37-1182.19) | 1.21 (1.1-1.31) |
| Ireland | 1048 (739-1463) | 121.8 (85.91-169.93) | 2510 (1614-3616) | 187.8 (120.36-271.28) | 1.46 (1.38-1.53) |
| Israel | 2066 (1553-2691) | 177.4 (133.58-230.91) | 4425 (3003-6300) | 190.2 (128.83-270.86) | 0.23 (0.17-0.29) |
| Italy | 24706 (18307-32907) | 166 (122.82-221.52) | 31391 (21431-44425) | 210.8 (142.35-301.34) | 0.73 (0.64-0.83) |
| Jamaica | 2687 (2253-3196) | 575.2 (482.82-684.19) | 5505 (4168-7138) | 721.4 (546.11-935.46) | 0.45 (0.21-0.69) |
| Japan | 48545 (36089-64243) | 134.6 (99.91-178.66) | 63327 (42413-89336) | 206 (137.42-292.2) | 1.44 (1.33-1.55) |
| Jordan | 2908 (2224-3761) | 468.3 (360.09-605.56) | 14733 (10383-20150) | 515.3 (363.22-704.9) | 0.1 (-0.09-0.3) |
| Kazakhstan | 7990 (6095-10507) | 214.3 (163.12-282.42) | 19385 (13466-26957) | 378 (263.08-525.05) | 1.31 (1.04-1.57) |
| Kenya | 8275 (6527-10187) | 209.6 (165.96-257.52) | 28517 (21936-36292) | 260.1 (200.49-330.04) | 0.85 (0.71-1) |
| Kiribati | 237 (174-310) | 1574.8 (1149.55-2071.68) | 682 (493-929) | 2345.9 (1693.61-3196.46) | 1.37 (1.28-1.47) |
| Kuwait | 1169 (863-1571) | 351.6 (260.56-470.12) | 9545 (6335-13509) | 558 (370.37-791.98) | 1.2 (0.99-1.41) |
| Kyrgyzstan | 1231 (938-1607) | 141.4 (106.42-185.6) | 4316 (3123-5890) | 258.4 (186.88-352.96) | 1.67 (1.49-1.86) |
| Lao People's Democratic Republic | 3992 (2761-5837) | 495.4 (342.84-722.15) | 9694 (6979-13179) | 530.4 (382.39-719.52) | -0.06 (-0.17-0.05) |
| Latvia | 1155 (918-1468) | 167 (133.09-211.29) | 1367 (1048-1817) | 290.2 (224.54-382.08) | 1.29 (0.76-1.83) |
| Lebanon | 3053 (2238-4095) | 448.8 (329.12-602.43) | 7905 (5555-10925) | 500.9 (351.9-692.57) | 0.44 (0.33-0.55) |
| Lesotho | 579 (405-791) | 174.3 (121.81-238.1) | 1943 (1298-2829) | 471.1 (312.14-690.52) | 4.41 (3.8-5.01) |
| Liberia | 1309 (927-1785) | 287.7 (204.88-392) | 6385 (4546-9006) | 504.8 (360.7-709.86) | 1.81 (1.65-1.97) |
| Libya | 1496 (1091-1981) | 224.1 (164.18-296.14) | 10971 (7719-14965) | 509.5 (358.28-695.49) | 3.1 (2.93-3.28) |
| Lithuania | 1115 (847-1473) | 116.7 (88.74-153.93) | 1465 (1071-2004) | 213.9 (157.74-290.33) | 1.63 (1.29-1.96) |
| Luxembourg | 139 (101-188) | 135.2 (97.49-182.19) | 385 (249-557) | 215.8 (138.84-312.84) | 1.46 (1.4-1.53) |
| Madagascar | 8004 (5896-10709) | 367.9 (270.56-492.61) | 25024 (17641-34061) | 403.3 (283.63-549.16) | 0.24 (0.15-0.33) |
| Malawi | 5660 (4029-7717) | 311.8 (222.36-424.87) | 11954 (8316-16655) | 296.1 (208.2-410.15) | -0.45 (-0.61--0.29) |
| Malaysia | 11621 (8818-15044) | 309.4 (234.85-400.61) | 29955 (22168-39793) | 367.3 (272.15-487.46) | 0.59 (0.49-0.7) |
| Maldives | 159 (115-218) | 452 (326.87-620.62) | 306 (218-422) | 255.2 (181.77-352.59) | -2.07 (-2.46--1.68) |
| Mali | 7558 (5546-10071) | 457.4 (335.87-610.15) | 29662 (21446-40331) | 658.5 (478.65-894.2) | 1.25 (1.07-1.43) |
| Malta | 175 (135-226) | 171.2 (131.96-221.06) | 328 (223-461) | 297.4 (201.04-419.17) | 1.94 (1.79-2.09) |
| Marshall Islands | 104 (77-139) | 1513.7 (1113.34-2010.14) | 490 (335-682) | 3452.8 (2354.78-4798.8) | 2.64 (2.28-3) |
| Mauritania | 1006 (718-1367) | 256 (182.67-348.65) | 3032 (2156-4155) | 332.7 (237.05-455) | 0.57 (0.46-0.68) |
| Mauritius | 1463 (1236-1727) | 564.3 (476.88-665.73) | 3635 (3060-4357) | 1028.6 (865.07-1233.1) | 2.58 (2.22-2.94) |
| Mexico | 139347 (115601-167118) | 807.6 (671.87-964.06) | 338015 (264060-424099) | 924.9 (722.2-1161.75) | 0.3 (0.18-0.43) |
| Micronesia (Federated States of) | 171 (121-242) | 1003.5 (706.03-1419.37) | 413 (297-552) | 1714 (1231.7-2287.2) | 1.88 (1.59-2.17) |
| Monaco | 8 (6-12) | 103.3 (69.71-148.95) | 18 (11-25) | 210.5 (135.34-301.74) | 2.34 (2.31-2.37) |
| Mongolia | 655 (488-842) | 159.4 (118.75-204.6) | 2277 (1646-3135) | 252.4 (182.48-346.99) | 1.31 (1.16-1.46) |
| Montenegro | 281 (207-370) | 183.7 (135.76-242.28) | 401 (279-560) | 236 (164.87-328.56) | 0.86 (0.64-1.08) |
| Morocco | 14207 (10307-19177) | 271.8 (197.33-366.45) | 71325 (48314-99363) | 716.6 (485.22-998.63) | 3.34 (3.23-3.46) |
| Mozambique | 8058 (5608-11337) | 298.2 (207.38-419.7) | 23485 (16186-33438) | 383.2 (264.01-547.33) | 1.13 (0.97-1.3) |
| Myanmar | 86337 (59167-125071) | 1007.8 (687.86-1459.43) | 119561 (88545-159038) | 787.7 (583.64-1047.53) | -1.3 (-1.49--1.12) |
| Namibia | 687 (506-917) | 266.1 (196.22-355.57) | 1886 (1294-2669) | 322.4 (221.07-456.57) | 0.33 (0.04-0.62) |
| Nauru | 27 (19-37) | 1345.3 (965.99-1876.05) | 53 (38-73) | 2119.4 (1505.11-2927.37) | 1.36 (1.15-1.57) |
| Nepal | 12795 (9394-17204) | 325.1 (239.82-436.74) | 42354 (30661-57749) | 508.1 (368.77-692.15) | 1.38 (1.07-1.68) |
| Netherlands | 5032 (3872-6409) | 122.7 (94.37-156.48) | 7119 (4714-10180) | 176.2 (116.66-252.11) | 1.07 (0.95-1.18) |
| New Zealand | 1499 (1178-1904) | 168.1 (132.23-213.44) | 2646 (1941-3562) | 204.1 (149.49-275.08) | 0.68 (0.31-1.04) |
| Nicaragua | 3006 (2280-3859) | 435.9 (331.18-558.63) | 9817 (7134-13230) | 569.3 (414.09-767.27) | 0.75 (0.67-0.84) |
| Niger | 3483 (2476-4829) | 239.8 (171.28-331.19) | 15445 (10778-21331) | 360 (252.41-495.54) | 1.13 (1.02-1.24) |
| Nigeria | 39940 (29580-52235) | 255.6 (190.41-332.38) | 131410 (94734-177677) | 276.8 (201.24-372.54) | 0.09 (0.01-0.17) |
| Niue | 4 (3-6) | 894.5 (637.85-1221.6) | 8 (6-10) | 1815.4 (1337.51-2388.42) | 2 (1.89-2.12) |
| North Macedonia | 1235 (937-1600) | 243.5 (184.68-315.43) | 1648 (1168-2267) | 259.2 (184.3-355.44) | 0.15 (-0.01-0.3) |
| Northern Mariana Islands | 72 (52-98) | 668.7 (477.12-916.38) | 114 (84-150) | 813.9 (597.96-1079.49) | 0.59 (0.48-0.7) |
| Norway | 1913 (1412-2565) | 174.5 (128.77-234.07) | 2776 (1917-3895) | 208.3 (143.48-293.14) | 0.38 (0.29-0.48) |
| Oman | 1078 (783-1448) | 391.3 (283.69-527.83) | 4474 (3196-5994) | 442 (315.83-591.99) | 0.02 (-0.12-0.16) |
| Pakistan | 73902 (56415-93350) | 364.8 (279.37-459.45) | 356512 (265170-463020) | 651.2 (485.07-845.06) | 1.72 (1.48-1.96) |
| Palau | 32 (23-44) | 905.8 (649.99-1222.94) | 72 (53-96) | 1530.4 (1135.86-2038.59) | 1.77 (1.67-1.87) |
| Palestine | 999 (716-1374) | 292.5 (210.1-401.44) | 4519 (3299-5989) | 405.6 (296.26-536.99) | 1.12 (1.01-1.23) |
| Panama | 1894 (1502-2378) | 361.7 (286.98-454.36) | 5415 (4028-7245) | 502.8 (374.16-672.58) | 1.05 (0.9-1.19) |
| Papua New Guinea | 6874 (4454-9896) | 888.2 (578.09-1280.55) | 29906 (21816-39858) | 1247.3 (910.42-1660.67) | 1.05 (1-1.09) |
| Paraguay | 2776 (2120-3610) | 346.1 (264.41-450.31) | 8600 (6430-11276) | 485.7 (363.43-636.9) | 1.18 (1.02-1.35) |
| Peru | 7495 (5683-9687) | 165.9 (126.03-214.05) | 20962 (15391-28021) | 215.7 (158.32-288.32) | 0.81 (0.57-1.06) |
| Philippines | 45837 (39178-53500) | 345.7 (295.91-402.31) | 117959 (95050-146600) | 429.4 (345.85-533.31) | 0.69 (0.53-0.86) |
| Poland | 18197 (14691-22552) | 188.3 (152.46-233.09) | 19281 (14155-25877) | 170.8 (125.8-228.96) | -0.22 (-0.52-0.07) |
| Portugal | 5371 (4066-7028) | 209.3 (158.43-273.92) | 9087 (6036-12916) | 325.9 (215-463.18) | 1.41 (1.31-1.5) |
| Puerto Rico | 5325 (4268-6701) | 557.7 (447.08-701.96) | 6179 (4460-8505) | 717.2 (517.43-987.42) | 0.67 (0.58-0.77) |
| Qatar | 244 (177-326) | 362.2 (263.33-486.06) | 3193 (2168-4497) | 549.7 (372.25-779.38) | 1.13 (0.91-1.35) |
| Republic of Korea | 30464 (23442-38829) | 263.6 (203.07-335.92) | 61799 (41146-89784) | 439.9 (293.15-637.43) | 1.91 (1.5-2.33) |
| Republic of Moldova | 1884 (1422-2487) | 169.2 (127.82-223.23) | 3294 (2393-4453) | 310.2 (228.03-415.58) | 1.38 (1.08-1.68) |
| Romania | 8072 (6326-10483) | 143 (112.24-185.41) | 7483 (5015-10618) | 140.1 (94.82-197.8) | -0.11 (-0.28-0.05) |
| Russian Federation | 49391 (39531-61869) | 133.6 (107.24-166.89) | 88685 (67743-115307) | 213.9 (164.94-276.09) | 0.77 (0.47-1.08) |
| Rwanda | 6347 (3976-9806) | 496 (306.22-776.08) | 8644 (5729-12800) | 281.2 (185.92-416.44) | -2.88 (-3.31--2.45) |
| Saint Kitts and Nevis | 69 (57-84) | 868.8 (720.55-1053.94) | 108 (78-149) | 623.4 (450.41-861.23) | -1.31 (-1.62--1.01) |
| Saint Lucia | 294 (242-358) | 1090.7 (900.24-1331.8) | 507 (379-680) | 979.3 (732.3-1311.38) | -0.36 (-0.52--0.19) |
| Saint Vincent and the Grenadines | 235 (197-282) | 1140.1 (952.16-1370.84) | 349 (272-446) | 1169.8 (910.75-1493.54) | -0.39 (-0.56--0.22) |
| Samoa | 216 (158-289) | 773.6 (569.16-1032.61) | 691 (492-932) | 1614.3 (1150.94-2174.2) | 2.5 (2.41-2.59) |
| San Marino | 7 (5-10) | 113.7 (77.57-158.61) | 18 (12-26) | 215.3 (140.53-310.7) | 2.12 (2.09-2.16) |
| Sao Tome and Principe | 40 (28-56) | 193 (134.44-267.78) | 191 (133-267) | 375.5 (261.72-524.66) | 2.01 (1.84-2.17) |
| Saudi Arabia | 7949 (5828-10682) | 316.2 (232.8-424.98) | 60931 (44959-81185) | 564.2 (415.49-752.19) | 2.05 (1.94-2.16) |
| Senegal | 5176 (3786-6965) | 361.5 (264.69-486.55) | 17856 (12958-24052) | 526.9 (383.51-708.56) | 1.4 (1.21-1.6) |
| Serbia | 5403 (4018-7137) | 221.2 (164.63-292) | 5496 (3837-7595) | 224.4 (157.35-308.86) | -0.18 (-0.3--0.05) |
| Seychelles | 36 (27-47) | 246.3 (186.71-321.15) | 144 (104-195) | 527.4 (382.62-713.68) | 2.89 (2.77-3) |
| Sierra Leone | 1831 (1305-2484) | 222.5 (159.64-300.35) | 8595 (6096-12076) | 448.8 (319.96-629.09) | 2.52 (2.28-2.76) |
| Singapore | 2262 (1695-3011) | 252.5 (189.27-336.29) | 5493 (3549-8006) | 309.1 (199.62-451.77) | 0.47 (0.26-0.69) |
| Slovakia | 1837 (1351-2436) | 134.8 (99.09-178.7) | 2534 (1740-3592) | 155.9 (107.63-219.87) | 0.64 (0.56-0.72) |
| Slovenia | 709 (541-931) | 136.5 (104.11-179.12) | 825 (562-1212) | 149 (102.07-217.54) | 0.18 (0.12-0.25) |
| Solomon Islands | 521 (286-806) | 974.9 (527.81-1518.89) | 2620 (1857-3598) | 1694.2 (1198.69-2327.46) | 1.88 (1.81-1.96) |
| Somalia | 5870 (3806-8639) | 404.5 (260.27-599.66) | 19128 (13014-26980) | 475.1 (321.23-673.29) | 0.45 (0.37-0.52) |
| South Africa | 36917 (31266-43662) | 462.1 (392.24-544.52) | 76687 (62199-93699) | 499 (404.99-608.82) | 0.81 (0.43-1.19) |
| South Sudan | 2958 (1937-4337) | 297 (194.22-436.26) | 8580 (5830-12245) | 414.9 (282.3-591.98) | 1.02 (0.73-1.31) |
| Spain | 15859 (11547-21362) | 165.3 (120.36-222.54) | 37360 (24555-54092) | 305.9 (200.88-443.07) | 2.02 (1.76-2.28) |
| Sri Lanka | 13497 (10106-17612) | 321.4 (240.78-418.55) | 33942 (24062-47612) | 560.2 (397.34-784.86) | 2.11 (1.91-2.32) |
| Sudan | 8355 (5950-11620) | 218.4 (155.9-302.39) | 39461 (27894-54116) | 406 (287.55-557) | 2.1 (2.05-2.15) |
| Suriname | 489 (364-635) | 572.1 (427.51-742.71) | 1414 (1049-1912) | 930.2 (689.76-1257.27) | 1.51 (1.36-1.66) |
| Sweden | 3934 (3018-5082) | 173.6 (132.68-225.52) | 5020 (3481-7000) | 205.8 (142.17-288.05) | 0.53 (0.42-0.64) |
| Switzerland | 3301 (2386-4486) | 173.4 (125.2-235.71) | 6446 (4145-9425) | 285.1 (182.62-417.43) | 1.63 (1.46-1.8) |
| Syrian Arab Republic | 6550 (4860-8591) | 303.5 (225.36-397.77) | 15634 (10935-21535) | 394.7 (275.84-543.94) | 0.64 (0.48-0.8) |
| Taiwan (Province of China) | 14702 (11726-18483) | 296.7 (237.17-372.58) | 19378 (14079-26172) | 281.5 (205.37-378.41) | -0.74 (-0.98--0.49) |
| Tajikistan | 2088 (1596-2658) | 223.7 (171.07-284.65) | 7241 (5305-9707) | 310.5 (227.34-416.48) | 0.52 (0.27-0.77) |
| Thailand | 38654 (28039-51413) | 277.9 (201.28-370.26) | 68705 (50114-90782) | 344.2 (251.75-455.47) | 0.1 (-0.31-0.51) |
| Timor-Leste | 346 (234-508) | 219.1 (148.86-320.79) | 959 (677-1296) | 337.9 (239.26-456.88) | 1.42 (1.07-1.77) |
| Togo | 1758 (1272-2390) | 246.4 (177.98-334.86) | 6654 (4692-9117) | 334.8 (235.99-458.02) | 1.01 (0.94-1.07) |
| Tokelau | 3 (2-5) | 1055.5 (752.65-1474.67) | 5 (4-7) | 1646.7 (1202.44-2201.4) | 1.21 (1.1-1.31) |
| Tonga | 187 (140-241) | 1042.7 (783.99-1341.39) | 344 (251-464) | 1473.8 (1077.11-1989.58) | 1.01 (0.95-1.07) |
| Trinidad and Tobago | 3308 (2825-3878) | 1239.1 (1058.86-1451.58) | 4427 (3401-5793) | 1160.5 (892.03-1517.12) | -0.55 (-0.71--0.38) |
| Tunisia | 2990 (2106-4079) | 178.2 (125.57-243.15) | 13631 (9444-19268) | 396.4 (274.13-560.72) | 2.54 (2.47-2.62) |
| Turkey | 33628 (24816-44894) | 273.4 (202.04-365.06) | 78357 (54066-109475) | 330.5 (228.13-461.34) | 0.81 (0.45-1.18) |
| Turkmenistan | 1409 (1171-1688) | 195.3 (161.7-234.92) | 5260 (4105-6733) | 422.5 (329.66-540.8) | 2.45 (2.17-2.73) |
| Tuvalu | 24 (17-33) | 1035.6 (732.88-1419.2) | 38 (28-52) | 1457.5 (1047.94-1970.22) | 1.08 (1.02-1.15) |
| Uganda | 6176 (4050-9009) | 213.6 (140.33-311.02) | 24922 (17312-34993) | 298.8 (208.32-419.78) | 0.36 (-0.02-0.74) |
| Ukraine | 23302 (18709-28907) | 177.5 (142.85-219.71) | 27185 (19351-36689) | 219.1 (155.63-295.4) | -0.42 (-0.82--0.03) |
| United Arab Emirates | 619 (446-826) | 237.2 (169.14-315.42) | 7275 (5045-10206) | 335.1 (233.02-470) | 1.06 (0.94-1.18) |
| United Kingdom | 23549 (17545-31018) | 159 (118.54-209.48) | 62596 (41806-88427) | 375.4 (249.66-532.2) | 2.74 (2.56-2.92) |
| United Republic of Tanzania | 12055 (8735-16286) | 254.2 (184.21-343) | 39164 (27774-53001) | 309 (220.29-417.38) | 0.56 (0.49-0.62) |
| United States of America | 149989 (126285-180306) | 216.5 (182.15-260.39) | 291833 (222712-378890) | 354.2 (270.31-460.22) | 1.5 (1.4-1.61) |
| United States Virgin Islands | 197 (148-260) | 634.3 (476.53-836.18) | 163 (114-226) | 801.5 (558.13-1109.37) | 1 (0.89-1.1) |
| Uruguay | 1001 (810-1246) | 132.4 (107.07-164.76) | 1877 (1380-2528) | 205.6 (151.17-276.81) | 1.29 (1.18-1.41) |
| Uzbekistan | 8129 (6614-10053) | 206.6 (167.29-257.99) | 37594 (28406-49842) | 414.4 (313.34-549.04) | 1.76 (1.43-2.1) |
| Vanuatu | 193 (129-275) | 722 (479.76-1033.6) | 905 (662-1202) | 1301.2 (951.58-1728.83) | 1.68 (1.59-1.77) |
| Venezuela (Bolivarian Republic of) | 15390 (12369-19189) | 382.9 (307.98-476.98) | 43299 (32376-56669) | 576.3 (431-753.97) | 1.25 (0.95-1.56) |
| Viet Nam | 35745 (25679-49263) | 272.2 (194.83-375.23) | 83865 (62269-112615) | 298.1 (221.15-400.35) | 0.23 (0.01-0.44) |
| Yemen | 3758 (2573-5573) | 178.4 (123.4-262.65) | 21204 (14371-30288) | 301.8 (205-431.38) | 1.04 (0.68-1.4) |
| Zambia | 6284 (4612-8388) | 440.7 (323.24-589.46) | 18537 (13171-25196) | 457.4 (327.13-619.91) | -0.29 (-0.45--0.13) |
| Zimbabwe | 3252 (2372-4364) | 180.8 (132.34-241.54) | 14501 (10142-20104) | 421.6 (293.82-587.57) | 3.72 (2.98-4.47) |

Note: ASR is per 100,000 women of childbearing age. EAPC, estimated annual percentage change; ASR, age-standardized rate; UI, uncertainty interval; CI, confidence interval.

**Supplementary Table 5.** Incidence of diabetes in women of childbearing age in 1990 and 2021 for 204 countries or territories, with EAPC from 1990 to 2021.

| Location | Case no. in 1990 (95% UI) | ASR in 1990 (95% UI) | Case no. in 2021 (95% UI) | ASR in 2021 (95% UI) | EAPC (95% CI) (%) |
| --- | --- | --- | --- | --- | --- |
| Afghanistan | 6228 (4373-8334) | 303.1 (211.96-406.67) | 55398 (39010-74067) | 847.3 (591.85-1136.09) | 3.4 (3.35-3.44) |
| Albania | 636 (456-855) | 89.7 (64.29-120.52) | 1014 (722-1343) | 154.2 (110.01-203.99) | 1.76 (1.7-1.82) |
| Algeria | 8662 (6250-11585) | 184.1 (134.01-244.94) | 58969 (43430-75757) | 504.5 (371.16-649.02) | 3.28 (3.25-3.32) |
| American Samoa | 54 (38-73) | 506.3 (357.18-676.99) | 157 (109-210) | 1290.3 (896.97-1737.45) | 2.93 (2.76-3.11) |
| Andorra | 13 (9-18) | 87.1 (60.21-120.6) | 42 (29-57) | 201.2 (142.44-270.46) | 2.76 (2.73-2.79) |
| Angola | 2394 (1669-3286) | 114.8 (79.8-157.67) | 16017 (11218-21576) | 222.9 (155.59-300.7) | 2.28 (2.19-2.37) |
| Antigua and Barbuda | 44 (32-59) | 303.6 (217.19-406.83) | 135 (97-178) | 506.1 (362.75-670.78) | 1.67 (1.66-1.69) |
| Argentina | 9480 (7012-12376) | 119.9 (88.66-156.52) | 28979 (21119-37321) | 232.8 (169.44-300.28) | 2.31 (2.26-2.37) |
| Armenia | 1088 (737-1505) | 139.5 (93.92-192.9) | 1989 (1382-2693) | 244.6 (169.79-330.99) | 1.79 (1.62-1.97) |
| Australia | 3430 (2472-4523) | 75.5 (54.34-99.72) | 8955 (6244-12309) | 135.8 (94.74-186.58) | 1.88 (1.79-1.97) |
| Austria | 1102 (778-1494) | 54.8 (38.75-74.07) | 2631 (1858-3527) | 130.4 (92.44-174.22) | 2.8 (2.73-2.88) |
| Azerbaijan | 1908 (1353-2560) | 118.5 (84.87-158.58) | 8480 (5974-11196) | 295.3 (208.14-389.72) | 3.05 (2.89-3.2) |
| Bahamas | 186 (136-243) | 291.8 (214.72-378.44) | 555 (397-743) | 491.7 (351.27-659.48) | 1.7 (1.65-1.76) |
| Bahrain | 232 (162-316) | 267.2 (187.09-364.36) | 2033 (1431-2751) | 598.8 (420.69-811.88) | 2.51 (2.42-2.6) |
| Bangladesh | 34489 (24448-46265) | 153 (108.65-204.71) | 170229 (120747-225983) | 374.9 (266.18-497.71) | 2.98 (2.86-3.1) |
| Barbados | 172 (122-225) | 269.5 (192.17-353.27) | 396 (288-522) | 490.2 (355.52-648.76) | 1.79 (1.67-1.91) |
| Belarus | 1909 (1331-2551) | 74.3 (51.82-99.22) | 3197 (2310-4230) | 129.8 (93.65-171.73) | 1.57 (1.45-1.7) |
| Belgium | 2404 (1646-3291) | 98.5 (67.49-134.74) | 5347 (3775-7267) | 211.6 (149.67-286.14) | 2.43 (2.39-2.47) |
| Belize | 85 (62-114) | 259.8 (190.68-346.18) | 570 (420-755) | 504.3 (372.28-667.17) | 2.24 (2.13-2.35) |
| Benin | 1163 (804-1610) | 120.6 (83.43-166.36) | 8664 (6025-11966) | 285.6 (198.02-397.94) | 2.6 (2.41-2.79) |
| Bermuda | 27 (19-35) | 151 (110.04-196.68) | 41 (30-55) | 267.6 (192.5-356.34) | 1.79 (1.71-1.86) |
| Bhutan | 176 (124-238) | 143.1 (101.58-192.5) | 525 (376-700) | 259.6 (185.61-345.88) | 1.94 (1.87-2.02) |
| Bolivia (Plurinational State of) | 1793 (1285-2367) | 136.5 (98.14-179.07) | 7627 (5451-10378) | 255.8 (182.9-348.2) | 2 (1.93-2.07) |
| Bosnia and Herzegovina | 1307 (911-1779) | 116.1 (80.82-158.21) | 2080 (1420-2841) | 233.5 (160.57-318.79) | 2.36 (2.28-2.43) |
| Botswana | 249 (173-340) | 92.1 (64.15-125.98) | 1194 (850-1615) | 180.2 (128.23-243.91) | 2.16 (2.12-2.2) |
| Brazil | 63249 (45924-84236) | 186.7 (136.11-248.58) | 151488 (108696-202962) | 240 (171.88-321.94) | 0.78 (0.68-0.89) |
| Brunei Darussalam | 110 (78-151) | 184.1 (130.37-253.21) | 728 (496-996) | 563.8 (384.46-770.96) | 3.87 (3.73-4) |
| Bulgaria | 2872 (2039-3851) | 126.3 (89.44-169.4) | 4202 (3178-5467) | 227.6 (171.17-297.6) | 2.02 (1.93-2.12) |
| Burkina Faso | 1498 (1054-2029) | 79 (55.67-107.08) | 8780 (6236-11902) | 174.1 (123.54-236.73) | 2.59 (2.49-2.7) |
| Burundi | 811 (556-1115) | 70.6 (48.38-97.15) | 3169 (2213-4308) | 109.7 (76.3-149.81) | 1.27 (1.19-1.34) |
| Cabo Verde | 80 (55-111) | 112.6 (76.73-156.31) | 348 (237-480) | 241.1 (164.89-332.02) | 2.71 (2.49-2.93) |
| Cambodia | 2074 (1497-2726) | 94.9 (68.51-124.48) | 8568 (6164-11498) | 197.5 (142.37-265.45) | 2.27 (2.19-2.35) |
| Cameroon | 1989 (1403-2675) | 94.1 (66.61-126.34) | 14367 (10133-19346) | 197.9 (139.39-267.45) | 2.47 (2.37-2.57) |
| Canada | 5127 (3689-6827) | 68.2 (49.21-90.69) | 19979 (14258-26826) | 215.3 (153.86-288.84) | 3.35 (3.22-3.47) |
| Central African Republic | 886 (605-1229) | 151.5 (103.53-210.2) | 4192 (2854-5812) | 319.5 (216.85-444.36) | 2.51 (2.42-2.59) |
| Chad | 1186 (826-1621) | 99 (69.07-135.05) | 7325 (5107-10031) | 209.6 (145.48-287.71) | 2.47 (2.32-2.61) |
| Chile | 3933 (2818-5367) | 119.4 (86-162.97) | 12045 (8136-16559) | 237.6 (160.92-326.09) | 2.31 (2.18-2.45) |
| China | 509613 (332055-729398) | 170.9 (110.95-245.04) | 856155 (585043-1179997) | 281.5 (197.37-380.01) | 1.44 (1.3-1.57) |
| Colombia | 15112 (10657-19884) | 196.3 (138.48-257.19) | 37309 (26517-49398) | 282.1 (200.54-373.42) | 0.68 (0.47-0.9) |
| Comoros | 80 (54-111) | 82.5 (56.23-114.99) | 317 (212-441) | 166 (111.11-231.26) | 2.39 (2.25-2.52) |
| Congo | 572 (399-786) | 115.1 (80.14-158.26) | 3193 (2210-4425) | 230.7 (159.47-320.26) | 2.24 (2.13-2.34) |
| Cook Islands | 25 (17-33) | 584.7 (405.65-783.48) | 50 (36-66) | 1123.6 (808.74-1472.06) | 2.07 (1.98-2.16) |
| Costa Rica | 1310 (933-1745) | 187.2 (134.25-248.63) | 4682 (3284-6285) | 354.6 (248.59-476.22) | 1.76 (1.64-1.87) |
| Coted'Ivoire | 2561 (1791-3479) | 107.3 (75.23-145.68) | 13671 (9532-18755) | 220.5 (153.59-303.01) | 2.42 (2.35-2.48) |
| Croatia | 1426 (984-1908) | 110.6 (76.29-148.16) | 2031 (1452-2747) | 188.5 (134.83-254.31) | 1.72 (1.68-1.77) |
| Cuba | 5720 (4124-7428) | 197.4 (142.6-255.6) | 8583 (6126-11494) | 313.2 (224.21-418.67) | 1.36 (1.27-1.45) |
| Cyprus | 203 (133-289) | 102.4 (67.38-145.81) | 691 (465-979) | 193.1 (130.99-270.12) | 1.99 (1.96-2.03) |
| Czechia | 3394 (2440-4403) | 118.1 (84.61-153.82) | 5991 (4316-8188) | 197.7 (142.47-267.99) | 1.66 (1.6-1.73) |
| Democratic People's Republic of Korea | 7556 (5338-10294) | 134.1 (94.71-182.93) | 18080 (12697-24352) | 264.2 (185.99-355.05) | 2.14 (2.12-2.16) |
| Democratic Republic of the Congo | 7356 (5178-9951) | 95 (66.92-128.27) | 38340 (26925-51567) | 192.9 (135.58-259.23) | 2.21 (2.17-2.25) |
| Denmark | 994 (726-1335) | 74.1 (53.74-99.66) | 2184 (1568-2911) | 166.3 (119.37-221.49) | 2.61 (2.53-2.7) |
| Djibouti | 49 (34-67) | 55.2 (38.36-75.82) | 337 (236-458) | 105.4 (73.55-143.2) | 2.05 (2.01-2.1) |
| Dominica | 48 (34-67) | 319.3 (225.17-444.84) | 89 (62-120) | 542.2 (377.16-727.52) | 1.68 (1.64-1.73) |
| Dominican Republic | 3298 (2383-4406) | 206.7 (150.41-274.86) | 12631 (9062-16900) | 444.2 (318.92-593.98) | 2.58 (2.52-2.64) |
| Ecuador | 2891 (2043-3910) | 134.8 (96.12-181.19) | 14528 (10382-19046) | 314.5 (224.71-412) | 2.92 (2.83-3.02) |
| Egypt | 12675 (9397-16449) | 108.8 (81.01-140.62) | 99767 (73183-131275) | 411.3 (302.46-541.25) | 4.5 (4.4-4.59) |
| El Salvador | 1791 (1300-2339) | 155.3 (113.29-201.48) | 6075 (4404-8160) | 345.3 (250.38-463.95) | 2.21 (2.06-2.36) |
| Equatorial Guinea | 102 (71-140) | 112.2 (77.81-153.9) | 825 (570-1141) | 243.7 (167.66-337.57) | 2.56 (2.53-2.58) |
| Eritrea | 527 (361-722) | 73.6 (50.63-100.5) | 2287 (1565-3136) | 144.7 (98.78-198.32) | 2.12 (2.09-2.15) |
| Estonia | 361 (258-492) | 89.5 (63.95-122.01) | 645 (467-856) | 201.5 (145.68-267.57) | 2.58 (2.47-2.7) |
| Eswatini | 192 (134-262) | 118.1 (83-160.98) | 599 (415-819) | 213.9 (147.95-293.19) | 2 (1.73-2.27) |
| Ethiopia | 10497 (7352-14254) | 100.8 (70.59-137.47) | 33488 (23267-45338) | 127.9 (88.49-173.9) | 0.52 (0.4-0.64) |
| Fiji | 777 (567-1014) | 438.4 (321.69-569.12) | 2176 (1624-2900) | 952.9 (711.26-1270.78) | 2.34 (2.26-2.42) |
| Finland | 1789 (1249-2441) | 138.6 (96.78-188.57) | 3490 (2464-4643) | 303.9 (215.18-403.37) | 2.42 (2.32-2.52) |
| France | 9034 (6338-12363) | 62.6 (43.91-85.7) | 19460 (13553-26368) | 134.3 (93.78-181.76) | 2.8 (2.71-2.9) |
| Gabon | 230 (159-318) | 117.6 (81.99-162.11) | 1113 (762-1540) | 241.2 (164.53-333.94) | 2.26 (2.16-2.35) |
| Gambia | 184 (129-248) | 94.2 (66.41-126.22) | 1150 (803-1557) | 207.1 (144.51-281.22) | 2.61 (2.54-2.68) |
| Georgia | 1426 (998-1936) | 105.4 (73.93-143.1) | 2537 (1804-3410) | 286.6 (203.48-384.96) | 3.37 (3.2-3.53) |
| Germany | 13819 (10047-18265) | 69.6 (50.61-91.9) | 34091 (24390-45665) | 193 (138.23-257.91) | 3.28 (3.17-3.39) |
| Ghana | 3548 (2471-4846) | 111.5 (78.19-152.01) | 17891 (12495-24307) | 207 (144.68-281.65) | 2.06 (2-2.12) |
| Greece | 2930 (1959-4040) | 114.3 (76.57-157.61) | 4744 (3240-6704) | 219.7 (151.47-307.2) | 2.09 (2.04-2.14) |
| Greenland | 5 (4-7) | 38.8 (28.13-53.76) | 16 (12-21) | 128.8 (92.62-166.44) | 3.96 (3.8-4.12) |
| Grenada | 58 (41-77) | 352.7 (255.46-467.48) | 157 (110-210) | 594.7 (416.93-797.27) | 1.67 (1.63-1.71) |
| Guam | 80 (57-106) | 244.1 (174.36-324.72) | 169 (124-220) | 456.3 (333.56-592.82) | 1.92 (1.85-1.99) |
| Guatemala | 3038 (2197-4043) | 188.2 (136.99-249.2) | 21013 (15113-28034) | 518.7 (373.38-689.39) | 3.5 (3.32-3.67) |
| Guinea | 1205 (849-1626) | 96.1 (67.84-129.91) | 5600 (3940-7621) | 184.6 (129.52-250.99) | 2.12 (1.87-2.37) |
| Guinea-Bissau | 282 (192-387) | 133.5 (90.99-183.35) | 1263 (869-1753) | 260.3 (178.61-361.83) | 2.15 (2.07-2.22) |
| Guyana | 893 (623-1205) | 539.2 (380.03-722.04) | 1881 (1325-2504) | 951.3 (669.63-1265.95) | 1.94 (1.85-2.03) |
| Haiti | 5666 (4022-7556) | 432.3 (309.89-573.23) | 23437 (16344-31385) | 698.5 (487.25-935.43) | 1.56 (1.53-1.59) |
| Honduras | 1836 (1305-2476) | 201 (143.79-269.73) | 10864 (7708-14407) | 415 (295.12-549.1) | 2.29 (2.22-2.36) |
| Hungary | 3485 (2591-4650) | 122.4 (90.69-163.3) | 5661 (4225-7405) | 203.1 (150.51-265.87) | 1.66 (1.52-1.8) |
| Iceland | 56 (39-77) | 87.8 (61.26-119.55) | 163 (116-218) | 203.2 (144.1-271) | 2.73 (2.68-2.77) |
| India | 284377 (202021-386740) | 150.7 (107.35-205.14) | 1059949 (756792-1409040) | 284.7 (203.42-378.5) | 1.93 (1.86-2.01) |
| Indonesia | 51179 (36797-68533) | 119.2 (86.04-159.6) | 171484 (125374-225644) | 217.5 (158.84-286.2) | 0.27 (-0.42-0.96) |
| Iran (Islamic Republic of) | 13289 (9545-17987) | 130.1 (94.2-176.11) | 86901 (62743-114789) | 340.8 (245.94-450.85) | 2.73 (2.62-2.85) |
| Iraq | 12149 (8557-16085) | 356.6 (251.69-469.31) | 80449 (57332-107958) | 807.9 (576.35-1082.45) | 2.77 (2.67-2.88) |
| Ireland | 823 (577-1117) | 94 (65.9-127.67) | 2142 (1530-2856) | 177.8 (126.88-237.17) | 2.12 (2.04-2.2) |
| Israel | 1317 (868-1884) | 111.5 (73.04-160.19) | 4221 (2852-5924) | 185.8 (125.66-260.31) | 1.65 (1.53-1.77) |
| Italy | 19078 (12624-27313) | 130.1 (86.04-186.13) | 24829 (15961-36143) | 190.9 (124.15-274.43) | 0.94 (0.81-1.08) |
| Jamaica | 1048 (774-1356) | 218.6 (162.54-281.51) | 2900 (2081-3798) | 378.7 (271.86-495.29) | 1.66 (1.59-1.74) |
| Japan | 42514 (29885-58552) | 120.4 (84.25-165.87) | 55122 (37916-77052) | 195.2 (134.72-270.41) | 1.31 (1.23-1.39) |
| Jordan | 1861 (1337-2476) | 300 (216.99-397.3) | 17320 (12515-22616) | 600.5 (434.25-783.29) | 2.43 (2.31-2.56) |
| Kazakhstan | 5559 (3891-7747) | 144.7 (101.63-202.57) | 15712 (11156-20645) | 318.2 (225.95-418.4) | 2.63 (2.55-2.72) |
| Kenya | 2550 (1781-3466) | 55 (38.39-75.03) | 9862 (6955-13311) | 81 (57.11-109.4) | 1.14 (1.07-1.2) |
| Kiribati | 84 (61-112) | 502.7 (363.17-663.61) | 287 (213-373) | 940 (698.71-1218.66) | 2.21 (2.08-2.35) |
| Kuwait | 1081 (767-1444) | 322.1 (231.01-426.95) | 11266 (8077-14887) | 684.5 (490.32-904.55) | 2.47 (2.4-2.54) |
| Kyrgyzstan | 831 (581-1110) | 90.3 (63.66-119.36) | 3677 (2611-4820) | 215.7 (153.14-282.47) | 2.97 (2.84-3.11) |
| Lao People's Democratic Republic | 1130 (818-1496) | 133.8 (97.34-176.56) | 5381 (3896-7032) | 288.6 (209.33-376.43) | 2.4 (2.36-2.45) |
| Latvia | 603 (416-821) | 87.4 (60.39-118.98) | 894 (622-1196) | 197.1 (138.23-263.43) | 2.56 (2.43-2.7) |
| Lebanon | 1638 (1196-2156) | 238.8 (175.62-312.85) | 7201 (5344-9420) | 467.2 (346.85-610.32) | 2.32 (2.23-2.4) |
| Lesotho | 258 (184-348) | 74 (52.63-99.85) | 841 (605-1112) | 189.5 (136.78-249.91) | 3.17 (3.11-3.23) |
| Liberia | 515 (360-703) | 106.4 (74.91-144.93) | 2905 (2028-3970) | 218.6 (152.4-298.89) | 2.4 (2.34-2.46) |
| Libya | 1280 (911-1726) | 183.4 (132.01-246.24) | 10622 (7659-14358) | 499 (358.79-675) | 3.57 (3.36-3.79) |
| Lithuania | 726 (498-991) | 76 (52.2-103.65) | 1082 (768-1461) | 167.6 (119.26-226.21) | 2.46 (2.36-2.56) |
| Luxembourg | 94 (65-130) | 95.8 (66.16-132.12) | 320 (224-436) | 202.1 (142.49-274.55) | 2.4 (2.38-2.43) |
| Madagascar | 1560 (1087-2122) | 62.9 (43.86-85.45) | 7494 (5225-10225) | 110.5 (76.94-150.9) | 1.75 (1.71-1.78) |
| Malawi | 1057 (743-1436) | 52.4 (36.85-71.24) | 3353 (2393-4491) | 71.8 (51.18-96.8) | 0.74 (0.6-0.88) |
| Malaysia | 7025 (4937-9396) | 178.9 (126.34-238.49) | 23079 (16541-30637) | 280.5 (201.26-371.66) | 1.39 (1.17-1.62) |
| Maldives | 51 (36-69) | 132.3 (94.93-179.2) | 239 (170-322) | 201.5 (143.06-271.2) | 1.28 (1.12-1.44) |
| Mali | 3047 (2056-4308) | 172.6 (116.1-244.88) | 16721 (11231-23491) | 327.6 (218.15-466.36) | 2.2 (2.01-2.38) |
| Malta | 96 (68-131) | 99 (69.56-134.41) | 262 (181-358) | 269.5 (186.86-365.7) | 3.35 (3.27-3.43) |
| Marshall Islands | 49 (34-66) | 573.9 (398.99-779.46) | 203 (143-269) | 1377.9 (972.95-1828.85) | 3 (2.83-3.17) |
| Mauritania | 383 (269-520) | 92.8 (65.28-126.02) | 1670 (1226-2165) | 176.2 (129.78-227.08) | 1.75 (1.6-1.9) |
| Mauritius | 618 (450-815) | 232.4 (169.65-305.73) | 1528 (1078-2007) | 449.4 (316.61-591.63) | 2.12 (2.01-2.24) |
| Mexico | 74170 (52942-99675) | 392.7 (281.31-528.57) | 193123 (139854-256599) | 538.7 (389.82-715.22) | 0.88 (0.71-1.06) |
| Micronesia (Federated States of) | 75 (54-99) | 367 (266.6-479.35) | 217 (160-280) | 859.3 (633.42-1113.42) | 3 (2.76-3.24) |
| Monaco | 6 (4-8) | 82.3 (56.92-113.22) | 14 (10-19) | 192.9 (136.98-259.52) | 2.8 (2.76-2.83) |
| Mongolia | 359 (252-484) | 81 (57.22-108.56) | 1688 (1237-2196) | 191.2 (140.06-249.11) | 2.83 (2.75-2.91) |
| Montenegro | 211 (150-284) | 137.6 (97.64-185.74) | 398 (290-532) | 238 (173.49-317.59) | 1.83 (1.77-1.9) |
| Morocco | 13033 (9172-17544) | 244.9 (173.46-328.77) | 77772 (55827-103396) | 785.7 (563.32-1045.68) | 3.91 (3.83-3.99) |
| Mozambique | 1518 (1075-2063) | 51.8 (36.79-70.47) | 7652 (5332-10707) | 110.1 (76.53-154.48) | 2.44 (2.39-2.48) |
| Myanmar | 18153 (12796-24181) | 199.3 (140.61-265.5) | 54907 (40971-70356) | 363.3 (271.3-465.57) | 1.53 (1.39-1.66) |
| Namibia | 283 (200-385) | 99.1 (70.76-134.28) | 967 (685-1312) | 156.9 (111.48-213.59) | 1.35 (1.28-1.43) |
| Nauru | 10 (7-14) | 455.6 (322.68-607.03) | 26 (18-34) | 956.1 (684.54-1255.19) | 2.07 (1.89-2.26) |
| Nepal | 6559 (4523-9027) | 154.3 (106.49-211.9) | 28886 (20440-39452) | 327.3 (231.22-447.95) | 2.3 (2.04-2.57) |
| Netherlands | 2948 (2048-4033) | 73.5 (50.98-100.44) | 5793 (4026-7968) | 153.5 (106.87-210.51) | 2.37 (2.31-2.42) |
| New Zealand | 1234 (870-1693) | 138.1 (97.27-189.66) | 2319 (1753-2935) | 182.9 (138.02-231.43) | 1.35 (0.91-1.8) |
| Nicaragua | 1658 (1177-2199) | 220.9 (157.68-291.12) | 7305 (5239-9916) | 418.1 (300.24-566.99) | 1.91 (1.82-1.99) |
| Niger | 1650 (1154-2255) | 106.3 (74.12-144.99) | 9814 (6901-13462) | 203.8 (142.22-280.02) | 2.12 (2.07-2.16) |
| Nigeria | 16213 (11400-22134) | 93.1 (65.72-127.49) | 72583 (51097-98853) | 139.3 (97.94-190.2) | 1.22 (1.16-1.28) |
| Niue | 2 (2-3) | 444.4 (315.08-591.77) | 4 (3-6) | 1040.3 (736.78-1373.84) | 2.8 (2.72-2.89) |
| North Macedonia | 724 (510-987) | 142.7 (100.39-194.72) | 1650 (1192-2250) | 257.9 (185.82-352.13) | 1.9 (1.81-2) |
| Northern Mariana Islands | 33 (24-44) | 261.8 (191.03-346.09) | 65 (47-85) | 514.2 (370.88-675.31) | 2.05 (1.99-2.11) |
| Norway | 1429 (1002-1952) | 133 (93.15-181.8) | 2253 (1560-3098) | 181.4 (125.89-248.51) | 0.82 (0.73-0.91) |
| Oman | 639 (461-845) | 226.4 (164.6-297.87) | 4029 (2888-5362) | 407.5 (292.11-540.76) | 1.18 (0.86-1.51) |
| Pakistan | 35098 (25090-47077) | 164.7 (118.14-220.72) | 203855 (145382-276120) | 355.6 (253.33-482.74) | 2.68 (2.46-2.9) |
| Palau | 18 (13-24) | 472.1 (334.95-627.47) | 43 (31-55) | 1003 (730.73-1295.08) | 2.49 (2.39-2.59) |
| Palestine | 614 (442-819) | 175.6 (126.97-232.98) | 4409 (3196-5765) | 389.8 (283.25-508.79) | 2.72 (2.6-2.85) |
| Panama | 949 (685-1290) | 171 (124.07-231.81) | 3781 (2716-5031) | 351.1 (252.15-467.35) | 2.19 (2.09-2.28) |
| Papua New Guinea | 2291 (1657-3017) | 260 (188.86-341.52) | 15702 (11371-20433) | 622.9 (451.02-810.17) | 2.91 (2.86-2.95) |
| Paraguay | 1348 (960-1864) | 169.3 (121.03-234.9) | 5337 (3961-6852) | 299.7 (223.34-383.75) | 1.92 (1.75-2.1) |
| Peru | 3992 (2862-5344) | 84.8 (61.03-112.77) | 13227 (9604-17374) | 136.4 (99.05-179.25) | 1.58 (1.35-1.8) |
| Philippines | 14746 (10395-20021) | 107.1 (75.48-145.63) | 48351 (34979-64423) | 173 (125.18-230.63) | 1.63 (1.45-1.82) |
| Poland | 14697 (10797-19372) | 148.7 (108.66-197.44) | 20405 (14605-27303) | 185.9 (132.89-248.86) | 0.7 (0.61-0.79) |
| Portugal | 3560 (2389-4952) | 139.1 (93.29-193.68) | 7353 (4925-10441) | 301.5 (203.05-424.11) | 2.47 (2.28-2.66) |
| Puerto Rico | 2678 (1935-3546) | 280.2 (202.3-371.11) | 4393 (3155-5792) | 527.3 (377.08-697.87) | 2.25 (2.08-2.42) |
| Qatar | 199 (143-273) | 290.6 (207.63-401.45) | 4025 (2750-5905) | 719.1 (488.26-1060.72) | 2.72 (2.63-2.81) |
| Republic of Korea | 19810 (14245-26512) | 171.1 (123.45-228.59) | 53002 (37417-70807) | 413.3 (293.2-549.51) | 3.23 (2.92-3.55) |
| Republic of Moldova | 1365 (936-1863) | 120.2 (82.6-163.64) | 2558 (1802-3387) | 246.8 (173.39-327.46) | 2.31 (2.11-2.51) |
| Romania | 5482 (3753-7449) | 96.8 (66.17-131.45) | 7758 (5653-10345) | 153.5 (111.61-205.22) | 1.46 (1.38-1.54) |
| Russian Federation | 31666 (22343-43078) | 83.6 (58.88-114.18) | 70941 (50485-94784) | 176.4 (125.42-235.65) | 2.29 (2.24-2.35) |
| Rwanda | 921 (642-1260) | 63.1 (43.92-86.03) | 2458 (1728-3308) | 73.6 (51.52-99.2) | 0.06 (-0.14-0.25) |
| Saint Kitts and Nevis | 27 (19-36) | 327.1 (232.09-442.34) | 83 (59-111) | 488.7 (348.69-655.95) | 1.24 (1.18-1.29) |
| Saint Lucia | 123 (85-168) | 441.1 (304.51-597.91) | 323 (236-419) | 649.4 (473.92-843.54) | 1.32 (1.26-1.39) |
| Saint Vincent and the Grenadines | 83 (58-113) | 385.8 (274-523.05) | 193 (135-256) | 656.8 (460.02-872.9) | 1.82 (1.78-1.87) |
| Samoa | 129 (93-171) | 401.6 (290.13-528.24) | 435 (311-575) | 940.4 (674.14-1240.97) | 2.79 (2.64-2.95) |
| San Marino | 6 (4-8) | 88.2 (61.62-120.69) | 15 (10-20) | 200.7 (141.33-272.62) | 2.71 (2.68-2.74) |
| Sao Tome and Principe | 26 (18-36) | 112.4 (77.27-155.27) | 133 (93-182) | 243.6 (169.2-334.21) | 2.51 (2.49-2.52) |
| Saudi Arabia | 5658 (4150-7368) | 216.7 (160.16-280.94) | 53498 (38881-70728) | 503.9 (366.17-665.21) | 2.7 (2.57-2.82) |
| Senegal | 2148 (1481-2946) | 140.1 (96.43-191.87) | 9315 (6451-12582) | 259.8 (179.13-352.39) | 2.26 (2.12-2.41) |
| Serbia | 3705 (2613-4996) | 150.5 (106.26-202.88) | 5883 (4237-7703) | 242.8 (174.84-319.03) | 1.5 (1.45-1.55) |
| Seychelles | 20 (14-27) | 127.1 (89.33-172.15) | 115 (79-159) | 432.8 (295.54-600.25) | 3.93 (3.76-4.09) |
| Sierra Leone | 875 (611-1191) | 98.5 (69.21-134.05) | 4589 (3182-6444) | 220.4 (152.32-309.89) | 2.7 (2.61-2.79) |
| Singapore | 2361 (1704-3104) | 264.7 (192.23-346.6) | 5176 (3696-6975) | 319.4 (227.06-431.22) | 0.36 (0.24-0.47) |
| Slovakia | 1448 (1011-1985) | 105.7 (73.7-144.95) | 2645 (1913-3524) | 166.9 (120.4-222.5) | 1.46 (1.38-1.53) |
| Slovenia | 530 (374-707) | 101.6 (71.92-135.61) | 859 (617-1142) | 163.4 (117.21-217.55) | 1.53 (1.43-1.63) |
| Solomon Islands | 169 (125-219) | 265.4 (197.31-342.95) | 907 (688-1153) | 551 (417.91-699.77) | 2.4 (2.33-2.46) |
| Somalia | 1108 (765-1529) | 69.6 (47.92-96.27) | 5568 (3862-7661) | 124.7 (85.94-172.6) | 1.79 (1.76-1.82) |
| South Africa | 12426 (8711-16884) | 141.6 (99.22-193.09) | 32682 (22574-44528) | 212.8 (147.05-290.06) | 1.19 (1.13-1.25) |
| South Sudan | 640 (441-890) | 55.5 (38.16-77.61) | 2242 (1559-3077) | 101.5 (70.26-139.84) | 1.91 (1.87-1.95) |
| Spain | 11990 (8209-16651) | 123.8 (84.7-172.15) | 28145 (18928-39430) | 271.8 (184.28-376.42) | 2.28 (2.17-2.39) |
| Sri Lanka | 6655 (4603-9017) | 155.3 (107.52-209.99) | 24290 (17246-32335) | 407.9 (289.03-544.18) | 3.14 (2.95-3.33) |
| Sudan | 6444 (4682-8497) | 166 (121.48-217.72) | 40273 (29488-51777) | 401.8 (295.32-515.22) | 2.81 (2.73-2.9) |
| Suriname | 250 (179-337) | 288.6 (206.97-387.2) | 938 (683-1241) | 623.9 (454.01-825.26) | 2.76 (2.68-2.84) |
| Sweden | 2453 (1659-3474) | 114.9 (77.63-162.04) | 4404 (2961-6233) | 193.9 (130.98-272.84) | 1.63 (1.54-1.73) |
| Switzerland | 2352 (1634-3179) | 129.7 (90.24-175.04) | 5617 (3918-7696) | 278.5 (194.88-379.45) | 2.57 (2.44-2.71) |
| Syrian Arab Republic | 4500 (3149-6068) | 200.3 (140.86-268.61) | 15951 (11588-21292) | 394.9 (285.59-527.94) | 2.15 (2.1-2.19) |
| Taiwan (Province of China) | 8410 (6167-10980) | 169.2 (125.59-219.36) | 15702 (11501-20431) | 235.2 (171.82-306.8) | 1 (0.86-1.15) |
| Tajikistan | 926 (669-1215) | 92.6 (67.77-120.64) | 5534 (3986-7333) | 230.9 (166.34-305.85) | 3.03 (2.93-3.12) |
| Thailand | 16128 (11702-21498) | 110.5 (80.3-147.25) | 38058 (27536-50509) | 200.8 (144.55-267.15) | 1.9 (1.83-1.97) |
| Timor-Leste | 143 (104-187) | 87 (63.89-113.57) | 684 (485-908) | 231.8 (164.41-307.43) | 3.19 (3.12-3.25) |
| Togo | 593 (421-804) | 78.3 (55.6-106.21) | 2808 (2017-3740) | 136.5 (98.03-182.15) | 1.75 (1.64-1.85) |
| Tokelau | 2 (1-2) | 492.5 (353.07-666.28) | 3 (2-4) | 977.9 (715.73-1284.56) | 2.19 (2.03-2.36) |
| Tonga | 88 (62-117) | 442.7 (314.26-590.77) | 213 (156-278) | 878.4 (642.17-1143.42) | 2.37 (2.29-2.44) |
| Trinidad and Tobago | 1293 (984-1601) | 477.2 (366.15-586.96) | 2513 (1781-3264) | 668 (472.63-869.55) | 0.94 (0.9-0.98) |
| Tunisia | 2780 (1968-3727) | 160.2 (114.14-214.3) | 14483 (10449-19059) | 431.4 (310.15-568.69) | 3 (2.8-3.2) |
| Turkey | 14174 (10592-18221) | 114.3 (85.95-146.28) | 71491 (53041-90873) | 306.9 (227.09-391.19) | 3.21 (2.82-3.6) |
| Turkmenistan | 587 (423-778) | 77.9 (56.33-102.59) | 2466 (1810-3177) | 197.7 (145.21-254.65) | 3.02 (2.91-3.13) |
| Tuvalu | 8 (6-10) | 323.5 (233.11-424.28) | 19 (14-25) | 703.2 (513.96-915.3) | 2.41 (2.36-2.46) |
| Uganda | 1637 (1133-2216) | 48.5 (33.56-65.76) | 7983 (5657-10782) | 83.6 (59.43-113.05) | 1.61 (1.54-1.68) |
| Ukraine | 12114 (8426-16554) | 92.7 (64.53-126.65) | 20802 (14401-28401) | 178.3 (123.63-242.71) | 1.95 (1.8-2.1) |
| United Arab Emirates | 557 (396-757) | 208.9 (149.05-285.3) | 8904 (6462-11624) | 412.5 (296.58-540.47) | 2.11 (2.03-2.19) |
| United Kingdom | 17815 (12167-24802) | 122.6 (83.6-170.7) | 50158 (36224-66379) | 336.9 (244.06-442.99) | 3.67 (3.42-3.92) |
| United Republic of Tanzania | 2505 (1767-3408) | 46.8 (33.22-63.65) | 13574 (9489-18489) | 99.3 (69.32-135.26) | 2.43 (2.35-2.52) |
| United States of America | 106105 (78134-139322) | 154.7 (113.79-203.53) | 279692 (214787-354593) | 342.9 (262.89-434.9) | 2.67 (2.63-2.72) |
| United States Virgin Islands | 118 (83-161) | 382.2 (267.92-522.24) | 131 (92-175) | 659.1 (463.25-885.28) | 1.95 (1.85-2.06) |
| Uruguay | 685 (495-890) | 90.6 (65.5-117.86) | 1963 (1419-2588) | 218 (157.19-288) | 3.15 (3.02-3.27) |
| Uzbekistan | 4183 (2980-5575) | 99.9 (71.85-131.95) | 26550 (19106-35585) | 291.9 (209.97-391.29) | 3.69 (3.49-3.89) |
| Vanuatu | 79 (57-104) | 252.4 (183.16-331.55) | 475 (347-620) | 636.3 (466.56-829.46) | 3.03 (2.97-3.08) |
| Venezuela (Bolivarian Republic of) | 8097 (5746-10644) | 189.7 (135.18-248.98) | 23866 (17041-31941) | 328.2 (234.05-439.81) | 1.77 (1.66-1.88) |
| Viet Nam | 13726 (9808-18089) | 94.9 (68.28-124.84) | 41988 (31355-53950) | 154 (114.78-198.09) | 1.24 (1.02-1.45) |
| Yemen | 3054 (2219-4051) | 134.5 (98.65-177.8) | 21299 (15468-28296) | 288.4 (209.83-383.27) | 1.72 (1.26-2.18) |
| Zambia | 1517 (1021-2137) | 91.2 (61.07-128.28) | 7784 (5262-10604) | 163.7 (110.38-224.75) | 1.67 (1.59-1.75) |
| Zimbabwe | 1718 (1214-2352) | 84.5 (60.09-115.58) | 6433 (4457-8802) | 177.2 (122.86-242.65) | 2.51 (2.47-2.55) |

Note: ASR is per 100,000 women of childbearing age. EAPC, estimated annual percentage change; ASR, age-standardized rate; UI, uncertainty interval; CI, confidence interval.

**Supplementary Table 6.** Deaths of diabetes in women of childbearing age in 1990 and 2021 for 204 countries or territories, with EAPC from 1990 to 2021.

| Location | Case no. in 1990 (95% UI) | ASR in 1990 (95% UI) | Case no. in 2021 (95% UI) | ASR in 2021 (95% UI) | EAPC (95% CI) (%) |
| --- | --- | --- | --- | --- | --- |
| Afghanistan | 116 (70-193) | 5.8 (3.5-9.7) | 522 (307-869) | 8.7 (5.1-14.56) | 1.38 (1.32-1.43) |
| Albania | 4 (3-6) | 0.6 (0.4-0.75) | 2 (1-3) | 0.3 (0.22-0.48) | -1.27 (-1.53--1.02) |
| Algeria | 71 (46-110) | 1.7 (1.06-2.55) | 242 (159-356) | 2 (1.35-3.02) | 1.07 (0.91-1.22) |
| American Samoa | 1 (1-1) | 9.1 (6.18-12.69) | 2 (2-3) | 17.6 (11.83-25.34) | 2.02 (1.58-2.46) |
| Andorra | 0 (0-0) | 0.6 (0.35-0.94) | 0 (0-0) | 0.4 (0.22-0.6) | -1.26 (-1.44--1.08) |
| Angola | 84 (53-124) | 4.7 (2.96-6.86) | 256 (159-391) | 4.1 (2.55-6.21) | -0.51 (-0.68--0.35) |
| Antigua and Barbuda | 1 (1-1) | 7.7 (6.55-9.02) | 1 (1-1) | 4 (3.46-4.66) | -1.98 (-2.19--1.77) |
| Argentina | 172 (150-197) | 2.2 (1.89-2.49) | 180 (154-208) | 1.4 (1.23-1.65) | -1.27 (-1.45--1.09) |
| Armenia | 21 (19-22) | 2.7 (2.52-2.9) | 15 (13-18) | 1.9 (1.6-2.26) | -0.88 (-1.43--0.32) |
| Australia | 33 (28-38) | 0.7 (0.62-0.83) | 41 (35-48) | 0.6 (0.51-0.7) | -1.06 (-1.35--0.77) |
| Austria | 16 (14-18) | 0.8 (0.65-0.88) | 6 (5-7) | 0.3 (0.22-0.3) | -3.23 (-3.4--3.06) |
| Azerbaijan | 34 (26-43) | 2.2 (1.69-2.78) | 54 (39-70) | 1.9 (1.4-2.49) | -1.15 (-1.61--0.68) |
| Bahamas | 5 (4-6) | 8.2 (6.88-9.57) | 6 (5-9) | 5.5 (4.03-7.36) | -1.64 (-1.91--1.37) |
| Bahrain | 4 (3-6) | 5 (3.54-6.86) | 16 (12-22) | 4.8 (3.47-6.42) | -0.07 (-0.4-0.27) |
| Bangladesh | 1070 (711-1504) | 5.4 (3.62-7.62) | 2266 (1486-3365) | 5.1 (3.38-7.65) | 0.46 (0.16-0.75) |
| Barbados | 5 (5-6) | 8.8 (7.58-10.25) | 5 (3-6) | 5.6 (4.17-7.54) | -1.57 (-1.77--1.37) |
| Belarus | 25 (21-28) | 1 (0.84-1.11) | 32 (24-41) | 1.3 (0.98-1.7) | -0.92 (-1.97-0.14) |
| Belgium | 20 (17-23) | 0.8 (0.67-0.9) | 8 (7-10) | 0.3 (0.24-0.33) | -3.6 (-3.86--3.33) |
| Belize | 2 (2-3) | 8.3 (7.11-9.69) | 8 (7-10) | 7.3 (5.99-8.76) | -0.34 (-0.77-0.09) |
| Benin | 24 (16-36) | 2.8 (1.83-4.21) | 87 (54-133) | 3.3 (2.03-5.01) | 0.29 (0.06-0.52) |
| Bermuda | 0 (0-1) | 2.7 (2.23-3.21) | 0 (0-0) | 1.1 (0.83-1.36) | -3.6 (-3.85--3.35) |
| Bhutan | 4 (2-6) | 3.4 (2.03-5.3) | 5 (3-8) | 2.8 (1.62-4.26) | -1.11 (-1.26--0.96) |
| Bolivia (Plurinational State of) | 64 (44-93) | 5.2 (3.51-7.5) | 115 (71-178) | 3.9 (2.43-6.11) | -1.2 (-1.35--1.05) |
| Bosnia and Herzegovina | 20 (15-27) | 1.8 (1.35-2.39) | 12 (8-17) | 1.3 (0.9-1.93) | -0.97 (-1.23--0.72) |
| Botswana | 8 (5-13) | 3.5 (2.01-5.74) | 19 (11-29) | 3 (1.8-4.64) | 0.59 (-0.23-1.41) |
| Brazil | 1444 (1369-1518) | 4.4 (4.13-4.58) | 1976 (1860-2101) | 3.1 (2.91-3.29) | -1.48 (-1.73--1.23) |
| Brunei Darussalam | 5 (3-7) | 8.9 (6.21-12.38) | 6 (4-8) | 4.5 (3.09-6.23) | -1.71 (-2.06--1.36) |
| Bulgaria | 50 (43-58) | 2.2 (1.91-2.59) | 30 (24-38) | 1.7 (1.31-2.08) | -1.13 (-1.38--0.89) |
| Burkina Faso | 73 (46-111) | 4.3 (2.71-6.55) | 149 (92-222) | 3.4 (2.14-5.06) | -0.77 (-0.9--0.64) |
| Burundi | 63 (37-97) | 6.5 (3.82-10.21) | 112 (65-176) | 4.6 (2.65-7.38) | -1.72 (-1.95--1.49) |
| Cabo Verde | 1 (1-1) | 1.4 (0.96-2.08) | 3 (2-5) | 2.3 (1.5-3.49) | 0.92 (0.06-1.78) |
| Cambodia | 98 (65-151) | 4.8 (3.18-7.45) | 165 (100-259) | 4 (2.41-6.23) | -0.88 (-1.06--0.7) |
| Cameroon | 83 (51-126) | 4.4 (2.71-6.7) | 308 (173-495) | 4.8 (2.74-7.75) | 0.21 (-0.12-0.54) |
| Canada | 101 (88-117) | 1.3 (1.14-1.52) | 90 (78-104) | 1 (0.83-1.1) | -1.5 (-1.85--1.16) |
| Central African Republic | 33 (20-51) | 6.7 (3.92-10.32) | 75 (44-118) | 6.6 (3.86-10.49) | -0.1 (-0.26-0.06) |
| Chad | 30 (19-47) | 2.9 (1.83-4.42) | 126 (75-198) | 4.2 (2.5-6.61) | 1.27 (0.93-1.62) |
| Chile | 44 (38-51) | 1.4 (1.18-1.58) | 35 (30-40) | 0.7 (0.58-0.79) | -2.05 (-2.34--1.75) |
| China | 3782 (2987-4651) | 1.4 (1.1-1.71) | 2625 (1965-3428) | 0.7 (0.5-0.88) | -2.75 (-3.04--2.46) |
| Colombia | 196 (169-226) | 2.8 (2.39-3.21) | 218 (173-270) | 1.6 (1.3-2.03) | -2.5 (-2.96--2.05) |
| Comoros | 5 (2-8) | 5.7 (2.87-9.11) | 8 (5-13) | 4.6 (2.69-7.3) | -1.13 (-1.49--0.76) |
| Congo | 29 (18-45) | 7.3 (4.57-11.27) | 96 (58-153) | 7.4 (4.48-11.8) | -0.22 (-0.53-0.1) |
| Cook Islands | 1 (0-1) | 19 (12.72-26.31) | 1 (0-1) | 12.4 (8.26-17.9) | -1.33 (-1.48--1.19) |
| Costa Rica | 12 (10-14) | 2 (1.73-2.34) | 23 (19-27) | 1.7 (1.43-2.03) | -1.14 (-1.84--0.42) |
| Coted'Ivoire | 57 (36-86) | 2.7 (1.73-4.14) | 213 (124-332) | 3.8 (2.23-5.92) | 1.68 (1.33-2.03) |
| Croatia | 15 (13-17) | 1.2 (1.05-1.37) | 7 (6-9) | 0.7 (0.56-0.83) | -1.58 (-1.84--1.32) |
| Cuba | 89 (77-103) | 3.2 (2.72-3.65) | 33 (26-40) | 1.1 (0.86-1.32) | -4.2 (-4.73--3.67) |
| Cyprus | 4 (3-6) | 2 (1.36-2.78) | 3 (2-4) | 0.7 (0.46-0.95) | -4.19 (-4.61--3.77) |
| Czechia | 34 (30-39) | 1.2 (1.06-1.38) | 21 (17-26) | 0.7 (0.6-0.88) | -1.15 (-1.54--0.76) |
| Democratic People's Republic of Korea | 115 (71-182) | 2.1 (1.31-3.35) | 166 (97-270) | 2.2 (1.32-3.63) | 0.14 (0.07-0.2) |
| Democratic Republic of the Congo | 319 (190-499) | 4.8 (2.88-7.58) | 802 (473-1258) | 4.7 (2.77-7.42) | -0.1 (-0.21-0.01) |
| Denmark | 13 (11-15) | 0.9 (0.74-1) | 4 (4-5) | 0.3 (0.25-0.34) | -4.05 (-4.51--3.58) |
| Djibouti | 3 (2-4) | 3.8 (2.25-5.98) | 12 (7-21) | 4 (2.25-6.81) | 0.14 (-0.1-0.38) |
| Dominica | 1 (1-1) | 7.1 (5.23-9.39) | 1 (1-2) | 6.9 (4.71-9.74) | -0.28 (-0.5--0.07) |
| Dominican Republic | 57 (41-78) | 3.8 (2.69-5.2) | 127 (86-181) | 4.5 (3.04-6.44) | 1 (0.76-1.25) |
| Ecuador | 60 (52-69) | 3.1 (2.65-3.53) | 129 (94-172) | 2.8 (2.05-3.75) | -0.53 (-1.1-0.03) |
| Egypt | 398 (280-549) | 3.6 (2.51-4.93) | 919 (607-1312) | 3.9 (2.54-5.51) | 0.69 (0.46-0.91) |
| El Salvador | 40 (30-51) | 3.9 (2.9-5.01) | 103 (70-144) | 5.9 (4-8.25) | 1.26 (1.04-1.47) |
| Equatorial Guinea | 5 (3-8) | 5.7 (3.44-9.39) | 14 (7-24) | 4.9 (2.52-8.2) | -0.66 (-0.83--0.48) |
| Eritrea | 42 (27-62) | 6.9 (4.41-10.15) | 91 (54-141) | 6.5 (3.88-10.08) | 0.11 (-0.03-0.24) |
| Estonia | 4 (3-5) | 1 (0.88-1.2) | 5 (4-6) | 1.5 (1.23-1.83) | -0.19 (-1.47-1.1) |
| Eswatini | 6 (4-9) | 4.4 (2.8-6.61) | 17 (9-30) | 7.2 (3.55-12.51) | 2.25 (1.13-3.37) |
| Ethiopia | 905 (674-1223) | 10.4 (7.79-14.11) | 805 (588-1069) | 3.7 (2.69-4.84) | -4.23 (-4.56--3.91) |
| Fiji | 42 (30-57) | 26 (18.62-35.71) | 77 (53-108) | 34 (23.3-47.5) | 0.9 (0.63-1.17) |
| Finland | 10 (8-11) | 0.7 (0.58-0.79) | 4 (3-5) | 0.3 (0.26-0.36) | -2.7 (-3.02--2.39) |
| France | 79 (69-91) | 0.5 (0.47-0.62) | 45 (38-52) | 0.3 (0.23-0.31) | -2.21 (-2.58--1.85) |
| Gabon | 9 (5-13) | 5.3 (3.3-8.01) | 21 (12-34) | 5 (2.86-8.19) | -0.35 (-0.73-0.05) |
| Gambia | 5 (3-7) | 2.8 (1.61-4.48) | 21 (12-34) | 4.3 (2.51-6.98) | 1.12 (0.82-1.42) |
| Georgia | 20 (18-23) | 1.5 (1.34-1.7) | 15 (13-17) | 1.6 (1.4-1.88) | 0.29 (-0.27-0.87) |
| Germany | 193 (167-223) | 0.9 (0.8-1.07) | 82 (70-95) | 0.4 (0.35-0.47) | -2.58 (-2.77--2.39) |
| Ghana | 128 (82-187) | 4.6 (2.94-6.69) | 315 (197-470) | 4 (2.51-5.99) | -0.43 (-0.68--0.18) |
| Greece | 11 (9-12) | 0.4 (0.35-0.47) | 8 (7-9) | 0.3 (0.23-0.31) | -1.73 (-1.89--1.57) |
| Greenland | 0 (0-0) | 1.6 (1.11-2.29) | 0 (0-0) | 0.8 (0.48-1.11) | -3.08 (-3.42--2.74) |
| Grenada | 2 (2-3) | 13.6 (11.39-15.95) | 2 (2-3) | 8.3 (6.52-10.4) | -1.78 (-2.1--1.45) |
| Guam | 1 (1-2) | 4.1 (2.98-5.44) | 2 (1-2) | 3.8 (2.74-5.07) | -0.09 (-0.6-0.42) |
| Guatemala | 63 (54-73) | 4.2 (3.61-4.84) | 486 (395-593) | 13 (10.54-15.84) | 2.96 (2.62-3.31) |
| Guinea | 46 (28-70) | 4 (2.41-6.04) | 131 (81-202) | 4.9 (3-7.46) | 0.74 (0.57-0.9) |
| Guinea-Bissau | 12 (7-17) | 6.3 (3.99-9.42) | 31 (18-47) | 7.3 (4.32-11.27) | 0.48 (0.24-0.72) |
| Guyana | 21 (17-27) | 13.7 (10.75-17.09) | 24 (16-34) | 12.5 (8.39-17.63) | -0.62 (-1.13--0.1) |
| Haiti | 243 (160-358) | 19 (12.49-27.88) | 489 (295-763) | 14.9 (8.99-23.29) | -0.59 (-0.69--0.49) |
| Honduras | 27 (18-38) | 3.3 (2.26-4.72) | 87 (50-139) | 3.6 (2.1-5.82) | -0.03 (-0.29-0.23) |
| Hungary | 47 (41-53) | 1.7 (1.45-1.9) | 24 (20-29) | 0.9 (0.75-1.09) | -1.98 (-2.32--1.64) |
| Iceland | 0 (0-0) | 0.3 (0.26-0.35) | 0 (0-0) | 0.1 (0.11-0.16) | -3.17 (-3.49--2.85) |
| India | 5052 (4325-5886) | 2.9 (2.48-3.36) | 10215 (8543-12122) | 2.8 (2.38-3.37) | -0.2 (-0.35--0.05) |
| Indonesia | 1598 (1276-2132) | 4 (3.19-5.3) | 2920 (2156-4035) | 3.6 (2.68-5.01) | -0.36 (-0.5--0.21) |
| Iran (Islamic Republic of) | 119 (95-148) | 1.3 (1.01-1.56) | 337 (295-389) | 1.3 (1.17-1.54) | 0.56 (0.41-0.7) |
| Iraq | 180 (119-258) | 5.9 (3.91-8.51) | 451 (280-691) | 4.7 (2.91-7.19) | -0.96 (-1.16--0.75) |
| Ireland | 4 (3-4) | 0.4 (0.38-0.51) | 2 (2-3) | 0.2 (0.15-0.21) | -3.19 (-3.52--2.87) |
| Israel | 15 (13-18) | 1.3 (1.14-1.54) | 12 (11-14) | 0.5 (0.44-0.6) | -2.97 (-3.28--2.66) |
| Italy | 123 (117-129) | 0.8 (0.77-0.85) | 45 (42-47) | 0.3 (0.25-0.28) | -3.5 (-3.65--3.35) |
| Jamaica | 35 (30-40) | 7.7 (6.74-8.86) | 53 (37-74) | 6.9 (4.82-9.71) | -0.69 (-1.06--0.32) |
| Japan | 239 (231-247) | 0.7 (0.63-0.67) | 60 (58-63) | 0.2 (0.18-0.2) | -3.96 (-4.56--3.35) |
| Jordan | 33 (23-45) | 5.4 (3.77-7.56) | 66 (45-94) | 2.3 (1.57-3.29) | -3.59 (-4.16--3.01) |
| Kazakhstan | 54 (48-61) | 1.5 (1.29-1.66) | 60 (51-70) | 1.2 (1.01-1.38) | -2.09 (-2.79--1.4) |
| Kenya | 98 (74-126) | 2.7 (2.02-3.41) | 328 (230-457) | 3.1 (2.2-4.35) | 0.85 (0.6-1.09) |
| Kiribati | 3 (2-5) | 23.9 (16.08-33.07) | 9 (6-14) | 32.2 (20.44-48.22) | 1.04 (0.94-1.15) |
| Kuwait | 7 (6-8) | 2.3 (1.91-2.63) | 16 (13-19) | 1 (0.77-1.16) | -3.6 (-4.38--2.82) |
| Kyrgyzstan | 9 (7-10) | 1 (0.83-1.16) | 20 (16-25) | 1.2 (0.93-1.53) | -0.15 (-0.59-0.29) |
| Lao People's Democratic Republic | 59 (37-95) | 7.6 (4.82-12.1) | 102 (63-153) | 5.7 (3.49-8.47) | -1.26 (-1.42--1.1) |
| Latvia | 11 (10-13) | 1.6 (1.42-1.91) | 10 (8-13) | 2.3 (1.86-2.75) | -0.13 (-1.57-1.34) |
| Lebanon | 35 (22-51) | 5.1 (3.31-7.62) | 35 (23-49) | 2.2 (1.48-3.12) | -2.97 (-3.23--2.71) |
| Lesotho | 6 (4-10) | 2 (1.2-3.11) | 24 (13-40) | 6.2 (3.23-10.29) | 5.59 (4.57-6.63) |
| Liberia | 15 (9-23) | 3.5 (2.09-5.34) | 63 (37-102) | 5.1 (3-8.27) | 1.2 (0.94-1.46) |
| Libya | 11 (7-16) | 1.7 (1.12-2.51) | 65 (39-103) | 3 (1.8-4.74) | 2.53 (2.29-2.77) |
| Lithuania | 8 (7-10) | 0.9 (0.77-1.03) | 9 (7-10) | 1.3 (1.04-1.55) | 0.29 (-0.88-1.47) |
| Luxembourg | 1 (1-1) | 0.7 (0.58-0.8) | 0 (0-0) | 0.2 (0.16-0.23) | -4.26 (-4.49--4.03) |
| Madagascar | 115 (77-163) | 5.5 (3.73-7.88) | 301 (185-461) | 5 (3.07-7.69) | -0.37 (-0.47--0.27) |
| Malawi | 83 (54-121) | 4.8 (3.13-7.05) | 148 (89-230) | 3.9 (2.35-5.97) | -1.01 (-1.24--0.79) |
| Malaysia | 122 (85-168) | 3.4 (2.36-4.68) | 217 (154-298) | 2.7 (1.92-3.72) | -0.64 (-0.93--0.34) |
| Maldives | 2 (2-3) | 7 (4.62-10.32) | 2 (1-3) | 1.5 (0.99-2.16) | -5.3 (-5.68--4.91) |
| Mali | 82 (54-121) | 5.2 (3.44-7.63) | 227 (146-339) | 5.3 (3.43-7.94) | -0.03 (-0.19-0.13) |
| Malta | 1 (1-2) | 1.3 (1.14-1.58) | 1 (1-1) | 0.7 (0.56-0.79) | -2.16 (-2.38--1.94) |
| Marshall Islands | 1 (1-2) | 20.8 (13.78-29.94) | 6 (3-10) | 44.7 (24.62-70.57) | 2.31 (1.83-2.81) |
| Mauritania | 12 (8-18) | 3.2 (2.01-4.8) | 29 (17-46) | 3.3 (1.91-5.17) | -0.22 (-0.44-0) |
| Mauritius | 20 (17-23) | 8 (6.84-9.16) | 47 (40-54) | 13.1 (11.16-15.08) | 2.49 (1.9-3.08) |
| Mexico | 1415 (1359-1472) | 8.6 (8.23-8.91) | 2916 (2410-3465) | 7.9 (6.52-9.37) | -0.61 (-1.11--0.11) |
| Micronesia (Federated States of) | 2 (1-3) | 14 (8.64-21.65) | 5 (3-7) | 20.5 (12.78-30.68) | 1.28 (0.94-1.63) |
| Monaco | 0 (0-0) | 0.2 (0.13-0.32) | 0 (0-0) | 0.2 (0.1-0.27) | -0.55 (-0.7--0.4) |
| Mongolia | 6 (4-8) | 1.5 (1.04-2) | 11 (8-15) | 1.2 (0.89-1.67) | -0.87 (-1.14--0.6) |
| Montenegro | 2 (2-3) | 1.5 (1.03-1.99) | 2 (1-3) | 1.2 (0.83-1.67) | -0.4 (-0.9-0.1) |
| Morocco | 90 (57-133) | 1.8 (1.12-2.61) | 287 (169-464) | 2.9 (1.69-4.65) | 1.89 (1.65-2.14) |
| Mozambique | 120 (75-181) | 4.6 (2.87-6.92) | 280 (160-447) | 4.8 (2.73-7.7) | 0.57 (0.35-0.8) |
| Myanmar | 1381 (881-2117) | 16.7 (10.58-25.52) | 1502 (973-2171) | 9.9 (6.4-14.26) | -2.21 (-2.43--2) |
| Namibia | 8 (5-12) | 3.4 (2.26-4.9) | 19 (10-31) | 3.3 (1.87-5.5) | -0.54 (-1.01--0.07) |
| Nauru | 0 (0-1) | 19.9 (12.5-29.8) | 1 (0-1) | 26.9 (16.54-41.64) | 0.93 (0.67-1.19) |
| Nepal | 123 (76-189) | 3.2 (2.02-4.96) | 255 (156-391) | 3.2 (1.94-4.87) | -0.17 (-0.47-0.12) |
| Netherlands | 41 (35-47) | 1 (0.85-1.14) | 14 (12-17) | 0.3 (0.29-0.4) | -3.78 (-4.04--3.53) |
| New Zealand | 13 (11-15) | 1.5 (1.28-1.65) | 9 (8-10) | 0.7 (0.62-0.78) | -2.87 (-3.23--2.51) |
| Nicaragua | 29 (21-38) | 4.3 (3.15-5.8) | 63 (42-88) | 3.7 (2.49-5.19) | -0.55 (-0.73--0.37) |
| Niger | 34 (20-54) | 2.5 (1.47-3.9) | 107 (62-173) | 2.6 (1.53-4.19) | -0.16 (-0.37-0.05) |
| Nigeria | 475 (319-684) | 3.2 (2.2-4.64) | 1184 (715-1840) | 2.6 (1.6-4.02) | -0.95 (-1.07--0.82) |
| Niue | 0 (0-0) | 10.4 (6.46-16.11) | 0 (0-0) | 17.5 (10.86-25.59) | 1.13 (0.95-1.31) |
| North Macedonia | 14 (10-18) | 2.7 (1.97-3.63) | 10 (7-15) | 1.7 (1.13-2.44) | -1.5 (-1.82--1.18) |
| Northern Mariana Islands | 1 (1-1) | 8.8 (5.51-13.51) | 1 (1-1) | 6.7 (4.62-9.33) | -0.87 (-1.13--0.61) |
| Norway | 8 (8-9) | 0.8 (0.71-0.8) | 4 (4-5) | 0.3 (0.31-0.35) | -2.95 (-3.59--2.3) |
| Oman | 12 (7-18) | 4.4 (2.8-6.81) | 25 (16-36) | 2.5 (1.64-3.63) | -1.66 (-1.9--1.41) |
| Pakistan | 818 (585-1086) | 4.1 (2.94-5.43) | 3313 (2208-4786) | 6.2 (4.11-8.89) | 0.87 (0.53-1.21) |
| Palau | 0 (0-1) | 10.1 (6.42-15.41) | 1 (0-1) | 12.8 (8.28-18.51) | 0.83 (0.63-1.03) |
| Palestine | 10 (6-16) | 3.1 (1.9-4.94) | 27 (19-38) | 2.5 (1.69-3.45) | -0.62 (-0.76--0.48) |
| Panama | 18 (15-21) | 3.6 (3.03-4.13) | 34 (26-43) | 3.1 (2.43-3.97) | -0.35 (-0.63--0.06) |
| Papua New Guinea | 97 (53-154) | 13.2 (7.24-20.79) | 317 (208-465) | 13.6 (8.88-19.87) | 0.08 (0-0.15) |
| Paraguay | 31 (22-43) | 4 (2.82-5.39) | 86 (57-123) | 4.9 (3.26-7.03) | 0.85 (0.6-1.1) |
| Peru | 77 (54-105) | 1.8 (1.24-2.41) | 177 (114-262) | 1.8 (1.17-2.7) | -0.03 (-0.36-0.3) |
| Philippines | 565 (499-638) | 4.4 (3.91-5) | 1509 (1168-1911) | 5.6 (4.3-7.03) | 0.79 (0.65-0.92) |
| Poland | 156 (150-164) | 1.6 (1.56-1.71) | 74 (64-84) | 0.7 (0.59-0.77) | -2.48 (-3.21--1.76) |
| Portugal | 37 (32-43) | 1.4 (1.22-1.65) | 14 (12-17) | 0.5 (0.41-0.57) | -3.81 (-4.15--3.47) |
| Puerto Rico | 51 (44-59) | 5.4 (4.65-6.18) | 33 (26-41) | 3.7 (2.89-4.66) | -1.75 (-2.02--1.48) |
| Qatar | 2 (1-3) | 3.4 (2.16-5.05) | 11 (7-16) | 1.9 (1.24-2.79) | -1.8 (-2.34--1.25) |
| Republic of Korea | 280 (213-359) | 2.4 (1.86-3.13) | 88 (65-118) | 0.6 (0.45-0.82) | -4.67 (-4.86--4.48) |
| Republic of Moldova | 13 (11-15) | 1.2 (1.04-1.4) | 18 (15-21) | 1.8 (1.5-2.16) | -0.46 (-1.7-0.8) |
| Romania | 73 (63-84) | 1.3 (1.13-1.51) | 26 (21-30) | 0.5 (0.42-0.61) | -3.24 (-3.66--2.82) |
| Russian Federation | 369 (358-380) | 1 (0.99-1.04) | 515 (451-575) | 1.3 (1.15-1.46) | -0.78 (-1.53--0.01) |
| Rwanda | 100 (56-166) | 8.3 (4.61-13.88) | 107 (57-182) | 3.6 (1.92-6.15) | -3.9 (-4.4--3.4) |
| Saint Kitts and Nevis | 1 (1-1) | 10.7 (9.03-12.7) | 1 (0-1) | 3.1 (2.23-4.09) | -4.48 (-4.95--4.02) |
| Saint Lucia | 4 (3-4) | 13.6 (11.73-15.72) | 3 (3-4) | 6.4 (4.95-8.19) | -2.56 (-2.86--2.27) |
| Saint Vincent and the Grenadines | 3 (3-3) | 15.1 (12.91-17.68) | 3 (3-4) | 10.4 (8.37-12.81) | -1.88 (-2.14--1.62) |
| Samoa | 2 (1-3) | 8.6 (5.56-12.8) | 7 (4-10) | 15.8 (9.75-24.09) | 2.06 (1.98-2.15) |
| San Marino | 0 (0-0) | 0.3 (0.21-0.46) | 0 (0-0) | 0.2 (0.09-0.3) | -1.17 (-1.49--0.84) |
| Sao Tome and Principe | 0 (0-0) | 1.2 (0.73-1.86) | 1 (0-1) | 1.4 (0.86-2.3) | 0.01 (-0.51-0.54) |
| Saudi Arabia | 82 (51-124) | 3.4 (2.1-5.21) | 443 (282-660) | 4.1 (2.64-6.18) | 0.83 (0.7-0.95) |
| Senegal | 55 (36-81) | 4 (2.63-5.93) | 159 (99-248) | 4.9 (3.03-7.61) | 0.69 (0.41-0.98) |
| Serbia | 50 (37-66) | 2.1 (1.51-2.71) | 27 (20-36) | 1.2 (0.84-1.53) | -2.13 (-2.41--1.86) |
| Seychelles | 0 (0-0) | 2.6 (1.9-3.55) | 1 (1-1) | 3.2 (2.29-4.32) | 1.64 (1.33-1.95) |
| Sierra Leone | 17 (10-27) | 2.3 (1.36-3.44) | 71 (42-113) | 3.9 (2.34-6.21) | 2.19 (1.79-2.6) |
| Singapore | 14 (12-16) | 1.6 (1.36-1.84) | 3 (3-4) | 0.2 (0.18-0.24) | -6.6 (-7.2--6) |
| Slovakia | 15 (11-20) | 1.1 (0.82-1.47) | 10 (7-14) | 0.6 (0.45-0.91) | -1.17 (-1.37--0.97) |
| Slovenia | 6 (5-7) | 1.1 (0.95-1.29) | 2 (2-3) | 0.5 (0.36-0.58) | -2.93 (-3.12--2.74) |
| Solomon Islands | 8 (3-14) | 15.7 (6.49-27.07) | 38 (24-58) | 25.2 (16.02-38.05) | 1.63 (1.54-1.72) |
| Somalia | 89 (51-144) | 6.4 (3.63-10.36) | 243 (138-384) | 6.3 (3.54-9.98) | -0.12 (-0.23--0.01) |
| South Africa | 473 (402-555) | 6.2 (5.24-7.22) | 875 (706-1060) | 5.8 (4.66-7) | 0.92 (0.28-1.56) |
| South Sudan | 42 (24-67) | 4.5 (2.62-7.19) | 111 (65-178) | 5.5 (3.19-8.79) | 0.58 (0.19-0.98) |
| Spain | 84 (72-96) | 0.9 (0.75-1) | 33 (28-38) | 0.3 (0.22-0.3) | -4.06 (-4.21--3.9) |
| Sri Lanka | 148 (103-208) | 3.6 (2.48-5.04) | 240 (134-381) | 3.9 (2.21-6.23) | 0.83 (0.62-1.04) |
| Sudan | 78 (46-131) | 2.1 (1.25-3.54) | 228 (120-379) | 2.4 (1.29-4.03) | 0.62 (0.5-0.73) |
| Suriname | 5 (3-6) | 5.6 (3.84-7.63) | 10 (6-13) | 6.2 (4.16-8.74) | -0.01 (-0.34-0.31) |
| Sweden | 28 (24-31) | 1.2 (1.04-1.33) | 12 (10-15) | 0.5 (0.41-0.59) | -2.39 (-2.6--2.18) |
| Switzerland | 16 (13-18) | 0.8 (0.69-0.93) | 5 (4-6) | 0.2 (0.18-0.25) | -4.46 (-4.66--4.25) |
| Syrian Arab Republic | 57 (38-81) | 2.7 (1.82-3.85) | 69 (43-105) | 1.7 (1.06-2.58) | -1.97 (-2.38--1.57) |
| Taiwan (Province of China) | 143 (124-163) | 3 (2.61-3.41) | 105 (88-122) | 1.5 (1.27-1.76) | -3.04 (-3.51--2.57) |
| Tajikistan | 23 (17-32) | 2.6 (1.86-3.53) | 52 (33-73) | 2.2 (1.41-3.12) | -1.31 (-1.71--0.9) |
| Thailand | 499 (329-718) | 3.7 (2.45-5.32) | 685 (442-998) | 3.3 (2.15-4.84) | -1.24 (-1.83--0.65) |
| Timor-Leste | 4 (3-7) | 2.9 (1.67-4.8) | 8 (5-12) | 2.8 (1.7-4.33) | -0.13 (-0.72-0.46) |
| Togo | 21 (14-32) | 3.2 (2.03-4.7) | 68 (40-107) | 3.5 (2.08-5.53) | 0.44 (0.3-0.59) |
| Tokelau | 0 (0-0) | 12.9 (7.82-19.56) | 0 (0-0) | 16.2 (10.68-24.12) | 0.35 (0.21-0.5) |
| Tonga | 2 (2-3) | 14.1 (9.7-19.54) | 4 (2-5) | 15.4 (9.99-22.59) | 0.05 (-0.07-0.18) |
| Trinidad and Tobago | 46 (41-52) | 17.7 (15.64-20.09) | 41 (30-56) | 10.8 (7.81-14.58) | -2.04 (-2.33--1.74) |
| Tunisia | 16 (11-23) | 1 (0.67-1.42) | 47 (29-71) | 1.3 (0.84-2.06) | 0.94 (0.84-1.03) |
| Turkey | 388 (264-558) | 3.2 (2.21-4.67) | 293 (195-426) | 1.2 (0.81-1.78) | -2.91 (-3.24--2.58) |
| Turkmenistan | 16 (14-18) | 2.3 (2-2.57) | 54 (40-72) | 4.3 (3.21-5.81) | 1.98 (1.51-2.45) |
| Tuvalu | 0 (0-1) | 15.8 (10.06-23.57) | 0 (0-1) | 17.8 (11.39-27.16) | 0.42 (0.33-0.5) |
| Uganda | 81 (44-131) | 3 (1.63-4.83) | 288 (166-455) | 3.6 (2.1-5.75) | -0.32 (-0.84-0.21) |
| Ukraine | 202 (176-231) | 1.6 (1.36-1.77) | 118 (65-186) | 1 (0.59-1.63) | -3.27 (-4.03--2.49) |
| United Arab Emirates | 5 (3-7) | 2 (1.25-3.08) | 23 (15-34) | 1.1 (0.74-1.66) | -1.75 (-2.13--1.37) |
| United Kingdom | 130 (127-134) | 0.9 (0.86-0.9) | 77 (75-80) | 0.4 (0.43-0.46) | -1.89 (-2.1--1.67) |
| United Republic of Tanzania | 170 (113-244) | 3.8 (2.53-5.45) | 435 (269-656) | 3.6 (2.21-5.34) | -0.28 (-0.39--0.17) |
| United States of America | 1581 (1526-1639) | 2.3 (2.2-2.36) | 1600 (1525-1681) | 1.9 (1.84-2.03) | -0.81 (-1.06--0.56) |
| United States Virgin Islands | 2 (1-2) | 5.5 (3.81-7.89) | 1 (0-1) | 3.2 (1.86-4.89) | -1.08 (-1.37--0.8) |
| Uruguay | 11 (9-13) | 1.4 (1.22-1.66) | 11 (9-12) | 1.2 (0.98-1.34) | -1.29 (-1.52--1.05) |
| Uzbekistan | 80 (68-92) | 2.1 (1.78-2.41) | 278 (212-354) | 3.1 (2.36-3.93) | 0.4 (-0.19-0.99) |
| Vanuatu | 3 (1-4) | 10.4 (5.92-16.7) | 10 (6-15) | 15.1 (9.58-22.24) | 0.9 (0.77-1.03) |
| Venezuela (Bolivarian Republic of) | 148 (129-170) | 3.9 (3.35-4.41) | 346 (249-466) | 4.5 (3.27-6.12) | 0.2 (-0.38-0.78) |
| Viet Nam | 467 (293-713) | 3.7 (2.34-5.73) | 903 (579-1375) | 3.2 (2.04-4.83) | -0.41 (-0.62--0.21) |
| Yemen | 30 (15-61) | 1.5 (0.8-3.03) | 110 (57-214) | 1.7 (0.87-3.22) | 0.19 (0.03-0.34) |
| Zambia | 83 (55-121) | 6.2 (4.08-9) | 166 (97-261) | 4.4 (2.55-6.87) | -1.63 (-1.84--1.42) |
| Zimbabwe | 30 (20-44) | 1.8 (1.19-2.68) | 166 (101-262) | 5.1 (3.06-8.04) | 5.08 (3.76-6.41) |

Note: ASR is per 100,000 women of childbearing age. EAPC, estimated annual percentage change; ASR, age-standardized rate; UI, uncertainty interval; CI, confidence interval.


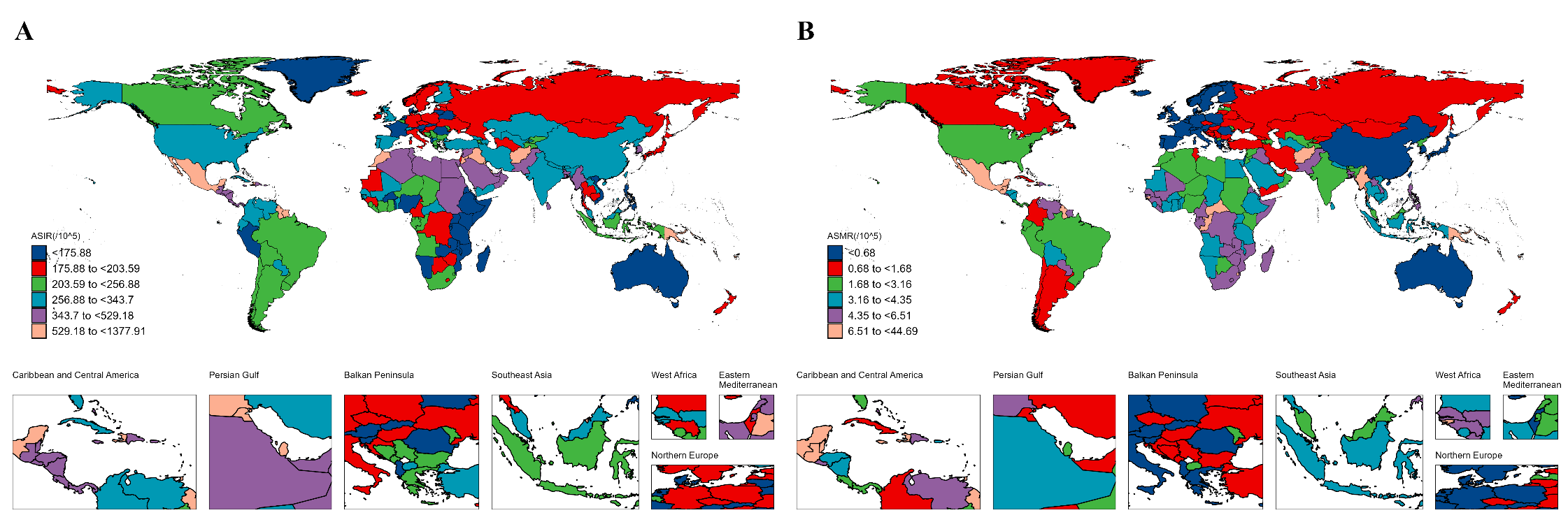


**Supplementary Figure 1.** Age-standardized rates of diabetes in women of childbearing age in 204 countries or territories in 2021. (A) Age-standardized incidence rate. (B) Age-standardized mortality rate.


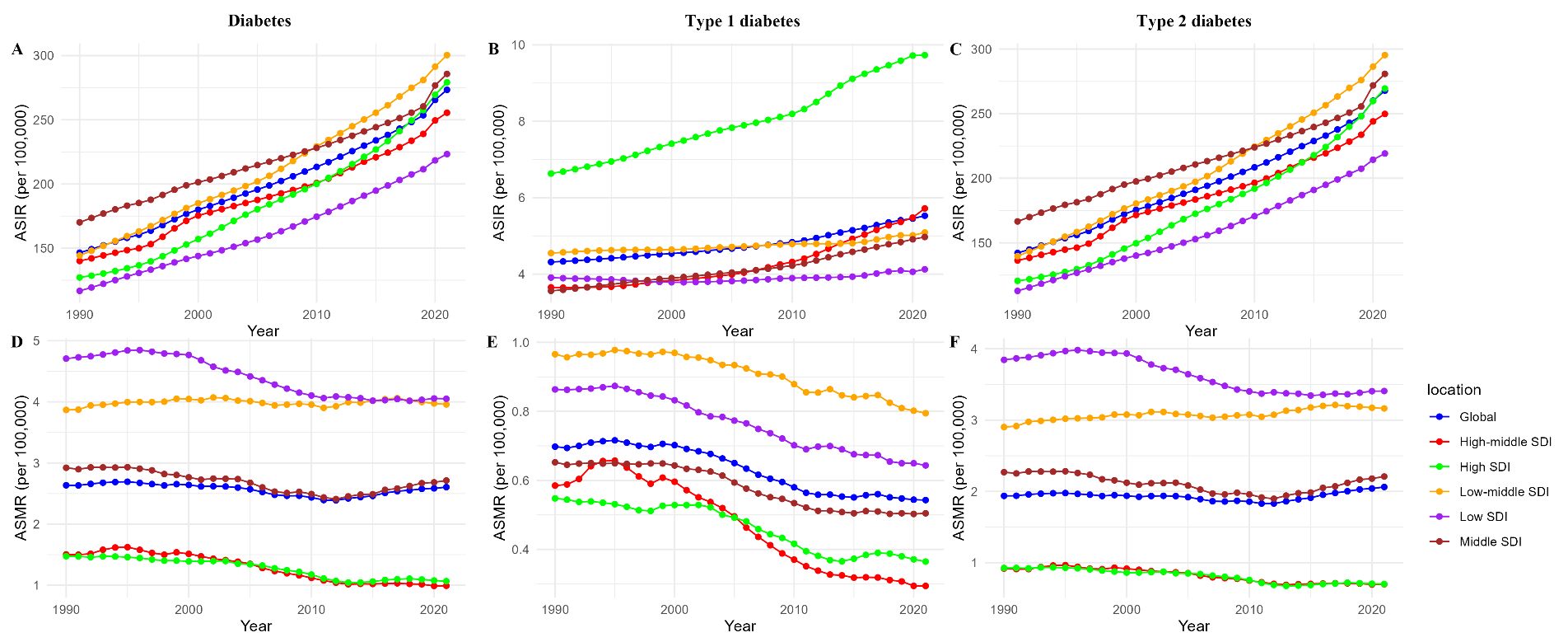


**Supplementary Figure 2.** Global and regional trends in age-standardized rates of diabetes among women of childbearing age, 1990-2021. (A) ASIR of diabetes. (B) ASIR of type 1 diabetes. (C) ASIR of type 2 diabetes. (D) ASMR of diabetes. (E) ASMR of type 1 diabetes. (F) ASMR of type 2 diabetes. ASIR, age-standardized incidence rate; ASMR, age-standardized mortality rate.


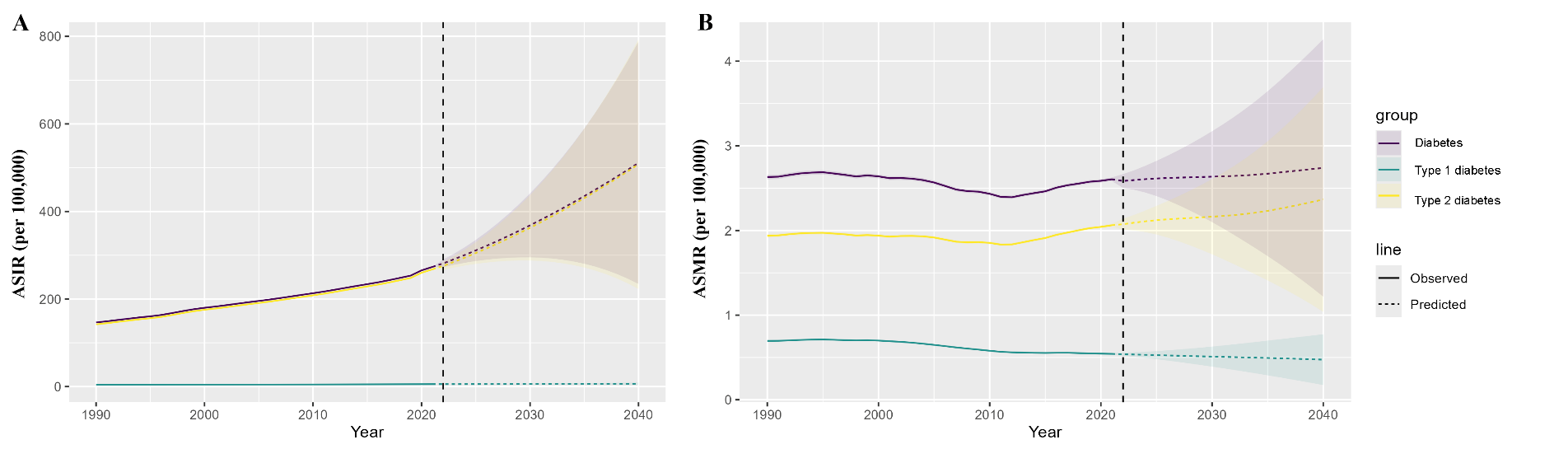


**Supplementary Figure 3.** Prediction of the global age-standardized rates of diabetes in women of childbearing age, 2022-2040. (A) Prediction of global ASIR. (B) Prediction of global ASMR. ASIR, age-standardized incidence rate; ASMR, age-standardized mortality rate.


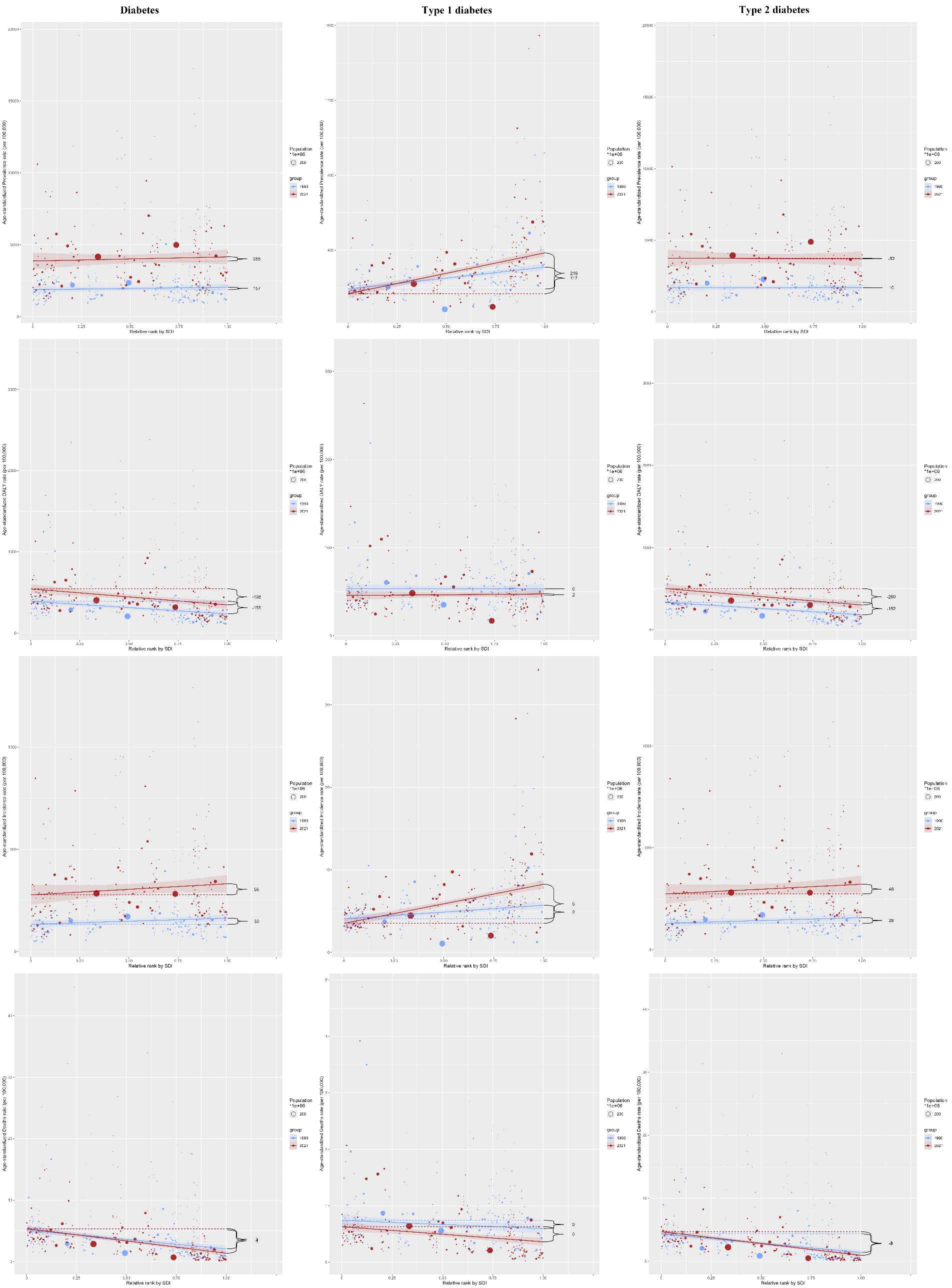


**Supplementary Figure 4.** Slope index analysis of diabetes in women of childbearing age in 204 countries or territories, 1990 and 2021. DALY, disability-adjusted life year.


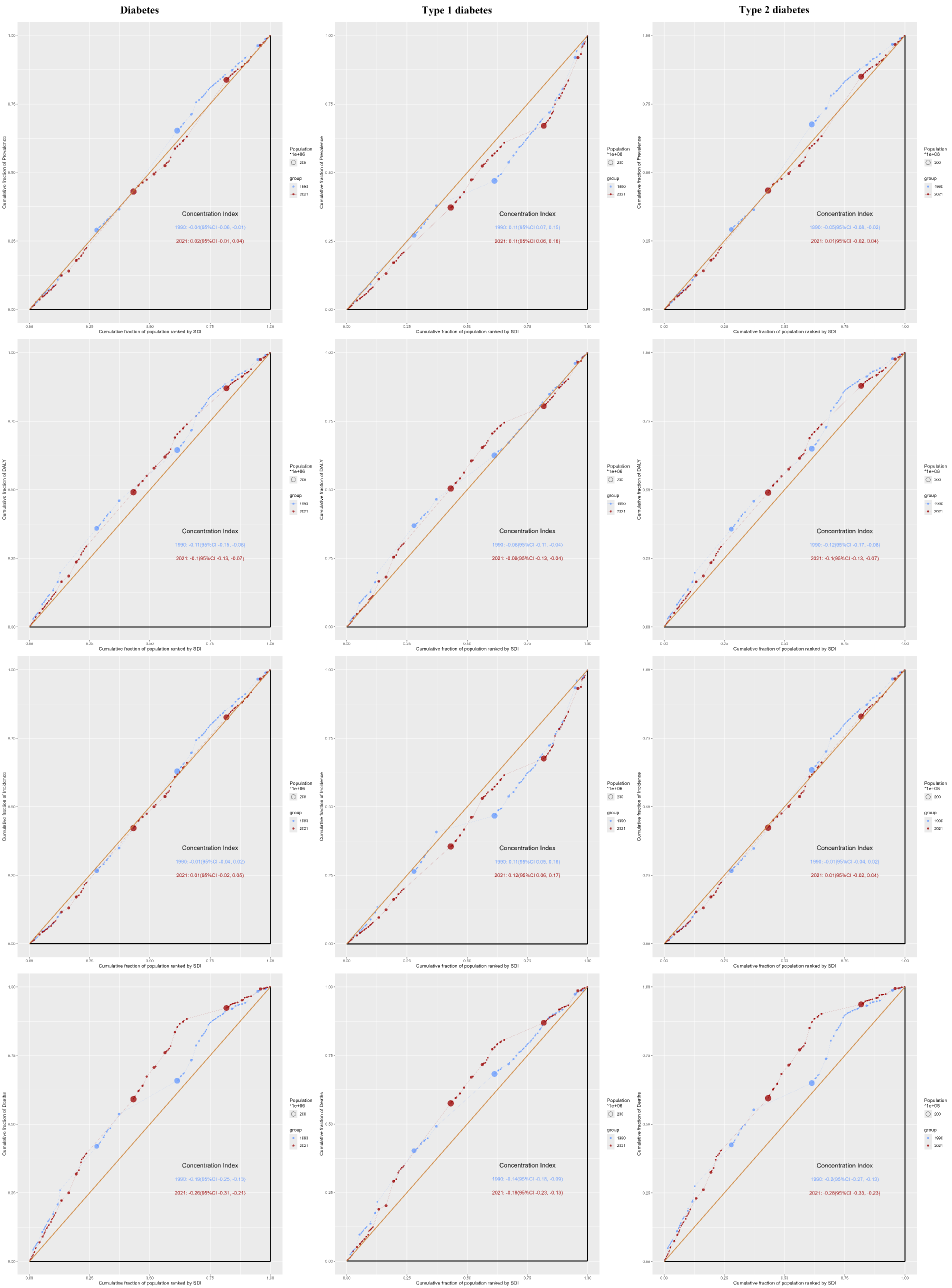


**Supplementary Figure 5.** Concentration index analysis of diabetes in women of childbearing age in 204 countries or territories, 1990 and 2021. DALY, disability-adjusted life year.
